# In Vitro Antibacterial Susceptibility of Different Pathogens to Thirty Nano-Polyoxometalates

**DOI:** 10.3390/ph15010033

**Published:** 2021-12-27

**Authors:** Ștefana Bâlici, Dan Rusu, Emőke Páll, Miuța Filip, Flore Chirilă, Gheorghe Zsolt Nicula, Mihaela Laura Vică, Rodica Ungur, Horea Vladi Matei, Nicodim Iosif Fiț

**Affiliations:** 1 Department of Cell and Molecular Biology, Faculty of Medicine, “Iuliu Hațieganu” University of Medicine and Pharmacy, 400349 Cluj-Napoca, Romania; sbalici@umfcluj.ro (Ș.B.); gnicula@umfcluj.ro (G.Z.N.); hmatei@umfcluj.ro (H.V.M.); 2 Department of Physical-Chemistry, Faculty of Pharmacy, “Iuliu Hațieganu” University of Medicine and Pharmacy, 400349 Cluj-Napoca, Romania; drusu@umfcluj.ro; 3 Department of Reproduction, Obstetrics and Veterinary Gynecology, Faculty of Veterinary Medicine, University of Agricultural Science and Veterinary Medicine, 400372 Cluj-Napoca, Romania; emoke.pall@usamvcluj.ro; 4 Analytical and Environmental Chemistry Laboratory, “Raluca Ripan” Institute for Research in Chemistry, “Babeș-Bolyai” University, 400294 Cluj-Napoca, Romania; filip_miuta@yahoo.com; 5 Department of Microbiology and Immunology, Faculty of Veterinary Medicine, University of Agricultural Science and Veterinary Medicine, 400372 Cluj-Napoca, Romania; flore.chirila@usamvcluj.ro (F.C.); nfit@usamvcluj.ro (N.I.F.); 6 Department of Medical Rehabilitation, Faculty of Medicine, “Iuliu Hațieganu” University of Medicine and Pharmacy, 400347 Cluj-Napoca, Romania; ungurmed@yahoo.com

**Keywords:** nano-polyoxometalates, UV, FTIR and NMR spectroscopy, drug designs, antibacterial activity, Gram-positive bacteria, Gram-negative bacteria

## Abstract

Due to their unique properties, nano-polyoxometalates (POMs) can be alternative chemotherapeutic agents instrumental in designing new antibiotics. In this research, we synthesized and characterized “smart” nanocompounds and validated their antibacterial effects in order to formulate and implement potential new drugs. We characterized thirty POMs in terms of antibacterial activity–structure relationship. The antibacterial effects of these compounds are directly dependent upon their structure and the type of bacterial strain tested. We identified three POMs that presented sound antibacterial activity against *S. aureus*, *B. cereus*, *E. coli*, *S. enteritidis* and *P. aeruginosa* strains. A newly synthesized compound K_6_[(VO)SiMo_2_W_9_O_39_]·11H_2_O (POM 7) presented antibacterial activity only against *S. aureus* (ATCC 6538P). Twelve POMs exerted antibacterial effects against both Gram-positive and Gram-negative strains. Only one POM (a cluster derivatized with organometallic fragments) exhibited a stronger effect compared to amoxicillin. New studies in terms of selectivity and specificity are required to clarify these extremely important aspects needed to be considered in drug design.

## 1. Introduction

Polyoxometalates (POMs) are a class of anionic polynuclear metal-oxo compounds of early transition metals synthesized by the “all in one pot” method [1,2], based on self-assembling mechanisms [3,4], or by two-stage methods involving ligand synthesis followed by cluster formation. They have multiple applications in fields such as catalysis [5,6], magnetism [7,8,9,10], electrochemistry [3,11,12,13], materials science [1,5,7,12,13,14], biology [15,16] and medicine [16,17,18] (presenting antidiabetic [19,20,21,22], antitumor [23,24], antiviral [25,26,27], or antibacterial [28,29,30,31] activities), due to their particular properties: high negative charge, redox behavior, shape, size, high solubility in water, etc. [12,14,16,18].

Metals (M) most often used in the synthesis of this class of compounds are vanadium (V), molybdenum (Mo) and tungsten (W), less frequent compounds involving tantalum (Ta) and niobium (Nb). POMs can be divided into two major classes: isopolyanions with the general formula [M_m_O_y_]^p−^ and heteropolyanions with the general formula [X_x_M_m_O_y_]^q−^, the latter category including heteroatoms (X) such as Si, P, Ge, Sb, As, Bi [1,2,7,14,16]. These compounds self-assemble into complete (saturated) archetypal structures such as the Anderson-Evans, Keggin or Wells-Dawson ones, or incomplete structures presenting 1–3 lacunes that could be occupied by identical or different metal ions [1,7,12,16,18]. Several parameters need to be strictly controlled during the syntheses. The process is influenced by temperature, pH, reducing agents, reactant concentrations, heteroatom nature and concentration, type of metal oxide anion involved, presence/absence of mixed addenda atoms and of additional ligands [1,3,12]. In order to design new biocompatible compounds with medical applications, POMs need to be derivatized/functionalized as these inorganic nanocompounds are not highly compatible with living organisms. This must be the goal of future research [14,16,18]. For over two decades POMs have been intensively studied for their antibacterial activity against both Gram-positive and Gram-negative reference strains, bacterial strains resistant to various antibiotics-including those in the β-lactam class (such as penicillins and cephalosporins) [16,18,28,29,30,31]. The finding of new natural [32,33,34] or chemically synthesized compounds [16,18,22,30] with antibacterial activity is a continuous challenge. As alternative chemotherapeutic agents, POMs have shown high antibacterial activity, being instrumental in creating new drugs to combat the antibiotic resistance of the bacteria.

Here we characterize the antibacterial activity of thirty nanoPOMs we synthesized in relation to their chemical structure. They include several Keggin-type nanocompounds presenting saturated (with/without mixed addenda atoms), mono-/tri-lacunary (with/without mixed addenda atoms, either or not sandwich type) structures, as well as one mono-lacunary Wells-Dawson polyoxometalate with mixed addenda atoms and also six clusters. The synthesized POMs were characterized using elemental and thermal analysis, UV and FTIR spectroscopy, while their potential antibacterial activity was evaluated against five bacterial species, two Gram-positive bacteria: *Staphylococcus aureus* (ATCC 6538P), *Bacillus cereus* (ATCC 14579) and three Gram-negative bacteria: *Escherichia coli* (ATCC 10536), *Salmonella enteritidis* (ATCC 13076), *Pseudomonas aeruginosa* (ATCC 27853), compared to amoxicillin (25 μg; Oxoid Ltd., Basingstoke, UK), a broad-spectrum antibiotic. 

## 2. Results 

### 2.1. Chemistry of Polyoxometalates

All thirty POMs we synthesized and characterized are presented in Table 1. One compound, a mono-lacunary Keggin with mixed addenda atoms, with a new formula proposed here, K_6_[(VO)SiMo_2_W_9_O_39_]·11H_2_O (POM 7), was first synthesized by us according to the methodology described in the Supplementary Material 1. Its characterization can be seen there.

The results of the chemical elemental analysis, thermal analysis, along with UV and FTIR spectroscopy data (in the Supplementary Material 2), are illustrated in Table 2. 

### 2.2. Antimicrobial Activity of Polyoxometalates

To compare the antimicrobial activity of all synthesized POMs versus amoxicillin, we measured the diameters of the inhibition zones (Halo Zone in mm) employing the disk diffusion method. Results are presented in Table 3. 

Of the thirty POMs whose antibacterial activity was tested with the disk diffusion method, nine nanocompounds exhibited no effects against the five bacterial strains: POMs 3, 5, 6, 9, 10, 12, 16a, 16b, 21 and 22.

**Figure 1:**
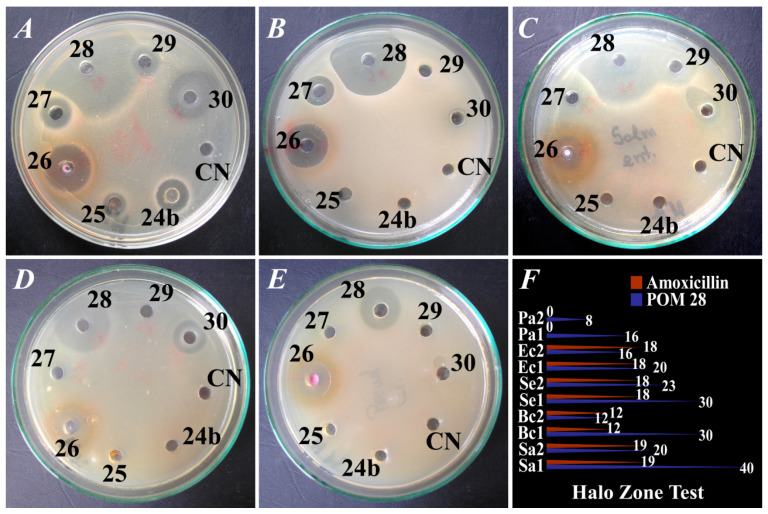
Antibacterial effects (assessed by the disk diffusion method) of various POMs (in black Arabic numerals) against (**A**). *Staphylococcus aureus* ATCC 6538P (abbreviated as Sa in panel F); (**B**). *Bacillus cereus* ATCC 14,579 (abbreviated as Bc in panel F); (**C**). *Salmonella enteritidis* ATCC 13,076 (abbreviated as Se in panel F); (**D**). *Escherichia coli* ATCC 10,536 (abbreviated as Ec in panel F); (**E**). *Pseudomonas aeruginosa* ATCC 27,853 (abbreviated as Pa in panel F); (**F**). Diameters of the inhibition zone (mm, values marked in white) of POM 28 (blue line), (NH_4_)_4_(NBu_4_)_5_[Na(BuSn)_3_Sb_9_W_21_O_86_]·17H_2_O compared to Amoxicillin (red line); Sa1, Bc1, Se1, Ec1, Pa1–initial testing; Sa2, Bc2, Se2, Ec2, Pa2–retesting after 6 months. The values of the Halo Zone Test (mm) are highlighted in white on the right side of panel F.

POMs which initially presented no activity against one strain, or another were not retested after 6 months against the respective bacterial strains. POMs 20, 26 and 28 were the only ones to present sound antibacterial activity against all five bacterial strains tested by the disk diffusion method, but only the latter constantly exhibited a stronger effect compared to amoxicillin. The antibacterial activity of POM 28 against all bacterial strains is illustrated in Figure 1.

The sensitivity of several Gram-positive and Gram-negative bacterial strains to the twenty-one POMs that exhibited antibacterial effects (via the disk diffusion method) was established by determining their minimum inhibitory concentration (MIC), our results being presented in Table 4 and in the Supplementary Material 2.

We also determined the sensitivity of several Gram-positive and Gram-negative bacterial strains to the twenty-one POMs that exerted antibacterial effects, their minimum bactericidal concentration (MBC) being presented in Table 5. In terms of the MBC, POMs that demonstrated antibacterial effects (via the disk diffusion method) were selectively tested against those bacterial strains on which they exerted bactericidal action.

## 3. Discussion

### 3.1. Chemistry of Polyoxometalates

The synthesis of polyoxometalates is a complex process involving molecular reorganization, the reaction mechanisms leading to the formation of new compounds being rather difficult to establish [14,26]. However, several synthesis possibilities are already well documented, such as the indirect synthesis replacing one polyoxoanion with another, or the “all in one pot” synthesis. All parameters need to be strictly controlled, including pH and temperature (essential in triggering the self-assembling mechanisms), the POMs composition and organic/inorganic nature of the solvent(s) or the presence of reducing agents, ionic strength, reflux, hydrothermal or ambient conditions, the type and concentration of oxoanions, the presence/absence and concentration of certain heteroatoms or addenda atoms [26]. For example, the pH of an aqueous solution of POMs needs to be increased (for V) or decreased (for W or Mo) during the more efficient “all in one pot” synthesis in order to increase the nuclearity of the oxoanion fragments [1,3,14,16,26,35]. Incorporation of heteroatoms, lacunary fragments, organic/organo-metallic fragments, transition-metal cations, or ligands significantly contributes to controllable structural changes in POMs’ size, shape and architecture, explaining their chemical variability [12,13,22,30].

The results of the **chemical elemental analysis** data are in agreement with the proposed chemical formula (see Table 1), and with the theoretical compositions (see Table 2).

**POMs’ thermogravimetric analysis** revealed the presence of two types of water molecules: coordinated water molecules (in our nanocompounds 1, 2, 4, 11, 21–27) and crystalization water molecules (in a specific number for each POM). The latter are eliminated first, in the second stage the removal of the coordinated water molecules paralleling the POMs’ decomposition [12,16]. POM 20 (a butyltin salt cluster) presented neither type, while in POM 2 only coordinated water molecules were observed. 

**UV electronic spectra** of all POMs (in 5 × 10^−5^ M aqueous solutions) exhibited two characteristic charge-transfer bands of high intensity [22,30] in the region of interest for polyoxoanions. Their contributions (as shown in Table 2) were attributed to specific POM bonds. The very small spectral displacements of the more intense bands, centered at ν_2_ ~ 200 ± 10 nm, corresponding to the p_π_(O_t_)→d_π_(M) transition, were attributed to the M=O_t_ double bonds, explaining the non-involvement of terminal oxygen atoms in the coordination structure for saturated/lacunary Keggin or *pseudo*-Keggin compounds, as well as their involvement in the coordination structure of all clusters (e.g., in MO_6_ octahedra). The broader spectral band displacements, generally centered at ν_1_~250 ± 20 nm (except for POM 1 and POM 19–at 228 nm, POM 5 and POM 6–at 305 nm), corresponding to the charge transfer transition p_π_(O_c,e_)→d_π*(_M), were attributed to the tri-centric bonds M-O_c,e_-M (bridge oxygen atoms connecting MO_6_ octahedra via their corners–O_c_, and via their edges–O_e_, respectively). They also explain the involvement of different oxygen atoms in coordination, as well as the spatial arrangement in each POM structure (lacunary/non-lacunary Keggin/*pseudo*-Keggin compounds, sandwich type or clusters). The second band was generally shifted towards lower energy levels in all cluster structures compared to their ligands because distortion of the MO_6_ octahedra due to an intensified inequivalence in these bonds decreases their original spatial symmetry. Our results are similar to other literature data [22,26,30].

The recorded **FTIR vibrational spectra** of POM salts fixed in KBr pellets as potassium salts for the (VO)^2+^ ions, and as sodium, butyltin, ammonium or tetrabutyl-ammonium salts for other POMs, exhibited characteristic bands for their structures [3,14,16,21,22,30]. For each POM, FTIR data were recorded for the 4000–400 cm^−1^ domain, and the polyoxoanion fingerprint region was found to be 1200–400 cm^−1^ [12,36,37,38,39]. Their contributions were assigned to specific POM bonds in correlation with their structures, as shown in Table 2. FTIR spectra similarities were observed between POMs of the same class, the shifting and splitting variations in our studied nanocompounds being explained by the influence exerted by addenda atoms, various heteroatoms or transitional metal cations coordination via different oxygen atoms. Our results are in agreement with literature data [14,16,22,30,35,36,37,38,39].

### 3.2. POM Pharmacology and Antimicrobial Activity

We found that the initial antibacterial effect of compounds 28, 15, 20, 19 against *S. aureus* was higher than that of amoxicillin. However, another test from the same solutions six months later revealed a drastic decrease of the antibacterial effect, only compound 28 maintaining a stronger activity than that of amoxicillin, although its effect was halved. 

This proves that the antibacterial effect of such compounds dissolved in saline buffer (0.15 M NaCl) is severely decreasing in time. Of the other POMs whose activity was lower than that of amoxicillin, compounds 13, 25, and 29 maintained or increased their antibacterial effect over the 6 months interval. 

The initial antimicrobial activity upon *B. cereus* was stronger (in compounds 28, 20, 15, 26, 27 and 19, in descending order) or equal (POM 2) to that of amoxicillin. After six months, only POM 20 exerted a greater effect than the reference, POMs 28, 26, 15 and 2 presenting a similar influence. Compounds 1, 13 and 30 maintained their initially lower than the reference antibacterial activity after the follow-up interval, while POMs 4, 8, 17 completely lost their action. 

Concerning Gram-negative bacteria, we found that POMs 28, 15, 19, 20 initially exhibited stronger antimicrobial activity against *S. enteritidis* than amoxicillin. Retesting 6 months later evidenced compounds 28 and 19 as stronger than amoxicillin in respect of their antimicrobial activity. In contrast, compound 29 completely lost its action. 

The only POM that initially exerted higher antibacterial activity than amoxicillin against *E. coli* was compound 28, but its effect fell below the reference level after six months. The compounds that maintained their antibacterial activity against *E. coli* after the six months interval were POMs 26, 30, 14, 20, 13, however to a much lesser extent than amoxicillin. 

We found that five compounds, 28, 26, 14, 20 and 1 (listed in descending order of their antibacterial effects), were the most effective upon *P. aeruginosa*, a bacterium resistant to amoxicillin. Six months after preparing the POMs solutions, compound 28 maintained its activity (even if its effect was halved), compounds 26 and 20 exhibited greater antibacterial effect than in the initial stage, while compound 14 diminished its activity and compound 1 completely lost its action. 

POMs 26 and 20 presented higher activity than amoxicillin upon all tested strains except for *E. coli*. Compounds 19 and 15 exerted a better antibacterial action than amoxicillin against strains of *S. aureus*, *B. cereus* and *S. enteritidis*, but *E. coli* and *P. aeruginosa* were found to be resistant to their action. In one-on-one comparisons, compound 2 presented similar activity to amoxicillin against *B. cereus*, as did compound 30 against *S. aureus* and compound 17 against *S. enteritidis*. Presumably higher concentrations of these compounds, closer to amoxicillin levels, would enhance their antibacterial action. Only nine POMs of the thirty tested presented no antibacterial activity in concentrations of 5 μg/well, i.e., compounds 3, 12, 16a, 16b, 21 and 22 (with Si as heteroatom), compounds 5, 6 (with P as heteroatom in their structures), and 9 and 10 (with As and Sb), respectively.

We found out that the strongest inhibitory concentration against *S. aureus* strains was exerted by compounds 20, 11, 23a, 24a and 30. Concerning cultures of *B. cereus*, the most powerful effect was observed in compounds 28, 20, 26 and 27, respectively. Good inhibitory concentrations against *B. cereus* strains were also found for compounds 15, 30, 17 and 19. The most powerful effect on *S. enteritidis* strains was once again exhibited by POM 28, followed by POMs 11, 20, 26. On cultures of *E. coli*, the best effect was validated for compounds 13, 28, and for 14, 20, 30, respectively. Of the five bacterial strains, POMs fared worst against *P. aeruginosa*. Still, a good inhibitory concentration was exhibited by compounds 28, 14, 1, 20, 26.

We found out that the minimum bactericidal concentration against *S. aureus* strains was rather high. The most pronounced bactericidal effect was induced by compounds 13, 14, 28 and 30, seconded by the group comprising compounds 1, 7, 11, 19, 20, 23a, 23b, 24a, 24b and 27. Against *B. cereus*, the minimum bactericidal effect was observed in POMs 28, 15, 20, 26, 27 and 30. Against *S. enteritidis,* the most effective compound proved to be no. 30, 15, 19 and 29. The most pronounced bactericidal effect against *E. coli* was noted in six compounds, 13, 14, 20, 26, 28 and 30. Only five POMs exhibited antibacterial activity against *P. aeruginosa* strain, four of which exerted bactericidal action, and one presented only bacteriostatic effect. On this Gram-negative strain, POMs 14, 28, 26 and 20 were the most efficient.

To conclude, in the initial testing three of the thirty analyzed “smart” nanocompounds (28, 20 and 26) presented antibacterial activity against all five bacterial strains tested, while POMs 1, 13, 14 and 30 missed one target (*E. coli* and *B. cereus*, respectively). POMs 2, 4, 7, 8, 23a, 23b, 24a, 24b, 25 and 27 exerted strong antibacterial effects against one or two Gram-positive strains, while POMs 18 presented antibacterial effects against one Gram-negative strain. All POMs exerting antibacterial effects on Gram-negative strains (1, 11, 13, 14, 15, 17, 18, 19, 20, 26, 28, 29, 30) were active against Gram-positive strains as well. When retesting after six months, we found out that some soluted POMs completely lost their antibacterial activity.

The new compound synthesized and characterized by us, K_6_[(VO)SiMo_2_W_9_O_39_]·11H_2_O (POM 7) presented antibacterial action only against *S. aureus*, with relatively high MIC and MBC (both 1.25 mg/L).

All results obtained using the disk diffusion method were concordant to the results obtained in terms of MIC and MBC and were in close interdependence with the POMs’ structures and the bacterial strain on which they were tested. In these terms, the selectivity and specificity of new antibacterial agents (POMs) is extremely important in drugs design. 

### 3.3. POM Structure-Antibacterial Activity Relationship

We managed to characterize the thirty tested “smart” nanocompounds (POMs) in terms of their antibacterial activity–structure relationship. The antibacterial effects of these compounds are directly dependent on their structure and the type of bacterial strain tested. In several compounds presenting monolacunary, trilacunary or trilacunary/sandwich Keggin structures (POMs 3, 5, 6, 9, 10, 16a, 16b) bacterial growth was not inhibited, as bacteria proved to be resistant to their action. Moreover, the monolacunary Keggin (POM 21) and the monolacunary Wells-Dawson (POM 22), both with mixed addenda atoms, did not exhibit antibacterial activity.

In small amounts (5 μg) cluster structures (POMs 20, 28) and trilacunary/sandwich *pseudo*-Keggin structures (POM 26) exhibited the strongest antibacterial effect on all bacterial strains tested, proving to be more efficient than the tested antibiotic (Amoxicillin, 25 μg). Other polyoxometalates such as nos. 1, 2, 4, 7, 8, 11, 13, 14, 17, 18, 23a, 23b, 24a, 24b, 25, 27, 29 or 30 presented selective and specific antibacterial effect against the bacterial strains tested. They may have had some bacteriostatic effect but are not bactericidal. 

Compared to uncomplexed salts, POM 20 (as tributyltin salt), POM 26 (as natrium salt), and POM 28 (cluster structure incorporating organo-metallic fragments, crystalized as tetrabutyl-ammonium and ammonium salt) presented an enhanced antibacterial effect against all five bacterial strains tested, including the Gram-negative *P. aeruginosa* strain against which amoxicillin had no effect.

Our results indicate that suppression of bacterial cell proliferation was initially inoculation-dependent and decreased in parallel with the progressive decrease of the POMs’ concentration, according to literature data [18,30,31,40]. 

Although mono-lacunary Keggin species were presumed to be more efficient than their saturated structures, our mono-lacunary Keggin (POM 21) and mono-lacunary Wells-Dawson (POM 22), both with mixed addenda atoms, did not present antibacterial effects. However, the antibacterial activity is not conditioned by the existence of the lacuna [31,40], because it was found that substituted lacunary Keggin structures present a higher antibacterial action than the original mono-lacunary structures, the effect being due to the transitional metal MT^n+^ occupying the lacuna [30]. 

The antibacterial activity of POMs was demonstrated on several Gram-positive and Gram-negative bacterial strains resistant to β-lactam antibiotics (penicillin, cephalosporins) against some reference bacterial strains. Yamase and co-workers noted that, in combination with β-lactamase inhibitors, POMs restore antibiotic effectiveness. They concluded that POMs with Keggin complete or lacunary structures, Wells-Dawson structures, or double sandwich Keggin structures enhance antibacterial activity against methicillin- (MRSA) and vancomycin-resistant *S. aureus* (VRSA) [31]. 

Similarly, polyoxoanions such as [KAs_4_W_40_O_140_]^27−^ or [KSb_9_W_21_O_86_]^18−^ demonstrated antibacterial activity against the *Helicobacter pylori* strain, which is resistant to metronidazole and clarithromycin. Ultrastructural changes into coccoid forms under the action of K_27_[KAs_4_W_40_O_140_]·H_2_O were evidenced by scanning electron microscopy [28]. *Helicobacter pylori* (ID3023) is a major pathogen associated with the development of duodenal and gastric cancer, duodenal ulcers, gastritis, and gastric ulcers, affecting about half of the world’s population [28]. World Health Organization 2018 data ranked this pathogen on top of the list in terms of incidence, mortality and prevalence of cancers attributable to infections [41]. Several possible mechanisms of POMs antibacterial actions have been described. For instance, these compounds may cross the peptidoglycans layer [30,40], or may penetrate the bacterial membrane via WtpABC and TupABC transporters [42], thus leading to the disintegration of the peptidoglycan layer and the dissolution of their membranes [28]. Inhibition of the DNA to RNA transcription by directly interacting with DNA molecules was also suggested for low-molecular-weight compounds able to electrostatically disrupt the cell envelope and penetrate into the bacterial cell [43]. We will address this problem in further studies.

As Yamase and co-workers postulated, some properties and characteristics of POMs, such as their redox behaviors, strong negative charges and chemical stability, are responsible for their strong antibacterial activity [18,30,44].

These nanocompounds destined to be the active ingredient of the potential pharmaceutical product are considered “smart” because of the way the huge clusters are formed (self-assembling of “block” units which then combine in “wheel” or “ball” structures) [12,45,46,47,48,49] and how they are modeled to specifically recognize targeted biological substrates. As a result of such properties, about 100 research studies relating POMs to cancer were published in the last decade [50]. These syntheses generate low amounts of chemical residues, being environmentally friendly, as literature data point out that the nanoPOMs’ maximal efficiency concentrations are often in the nanomolar range [22,23,51].

We studied the toxicity of two of our nanoPOMs (10, 28) in previous works. Based on our in vivo studies [21], we concluded that POM 28 and particularly POM 10 presented significant hypoglycemiant activity following oral treatment of rats with streptozotocin-induced diabetes. The main cause seems to be the prevention of pancreatic β-cells apoptosis, as observed by transmission electron microscopy (TEM), but our data also revealed stimulation of insulin synthesis by pancreatic β-cells in diabetic rats. Our TEM ultrastructural studies demonstrated the ability of POM 10 and POM 28 to prevent the hepatotoxicity of streptozotocin. Our in vitro studies revealed the significant biological activity of POM 10 and POM 28 as active stimuli for the differentiation of stem cells into insulin-producing cells [22]. MTT assay on human umbilical vein endothelial cells and human bone marrow adult mesenchymal stem cells proved the low, dose-dependent toxicity of POM 10 and POM 28 [22]. In view of the design of future drugs, identifying new compounds with strong antibacterial effects is an ongoing challenge addressing today’s technological advances. POMs have a real potential towards such a goal and our study opens new directions in future research. The limitations of our study are due to the scarcity of data on POMs’ stability in the culture media of different bacterial strains and at physiological pH (a prerequisite for drugs to be administered to humans), and of toxicity studies (a shortcoming that needs to be addressed taking into account that they are inorganic compounds).

## 4. Materials and Methods

### 4.1. Synthesis and Physico-Chemical Characterizations of Polyoxometalates

#### 4.1.1. Reagents and Chemical Materials

All solvents of analytical purity and all chemical substances used in the synthesis of polyoxometalates were purchased from Sigma-Aldrich (N.V./S.A., Bornem, Belgium or Co. LLC., St. Louis, MO, USA) and Merck (KGaA, Darmstadt, Germany), respectively. 

The list of analytically pure reagents used in the syntheses included acetone, acetonitrile, glacial acetic acid, HCl (37%), H_3_PO_4_ (85%), HClO_4_ (70%), sodium acetate trihydrate, trisodium citrate dihydrate, n-butyltin trichloride, tributyltin chloride, tetrabutylammonium bromide, KCl, NaCl, Na_3_VO_4_, VOSO_4_·5H_2_O, H_3_[PMo_12_O40]·13H_2_O, H_3_[PW_12_O_40_]·12H_2_O, H_4_[SiW_12_O_40_]·14H_2_O, Na_2_HAsO_4_·7H_2_O, Na_2_MoO_4_·2H_2_O, Na_2_HPO_4_·12H_2_O, Na_2_GeO_3_·H_2_O, Na_2_WO_4_·2H_2_O, Bi(NO_3_)_3_·5H_2_O, Sb_2_O_3_, CoCl_2_·6H_2_O, MnCl_2_·4H_2_O, FeCl_3_·6H_2_O, FeCl_2_·4H_2_O etc. 

Bi-distilled and deionized water produced with a FI-Streem III Cyclon Glass Still Bi-Distiller (Sanyo/Gallenkamp PLC, Cambridge, UK) was used for all solutions prepared during the POMs syntheses. All POMs were recrystallized from a minimum volume of bi-distilled water, crystals of different shapes and various colors were obtained. Only three POMs (16, 23 and 24) required double recrystallization. 

#### 4.1.2. Synthesis of Polyoxometalates

POMs are generally difficult to be synthesized, involving either solvothermal or hydrothermal methods requiring high pressure and temperature (implying high energy consumption) or ones using toxic organic solvents (incompatible with the development of green chemistry) [35,39,52,53,54]. We carefully selected the method based on each POM’s specifics, sometimes modifying protocols mentioned in literature [3,12,14,16,18,22,30,53]. 

Initially we employed the **two-step method**, involving the ligand synthesis followed by the cluster formation, for synthesizing ten POMs, i.e., the Keggin (saturated/mono-lacunary) structures with mixed addenda atoms, the mono-lacunary Wells-Dawson polyoxometalate with mixed addenda atoms, and the six clusters-type structures. Certain molar ratios of organic and organometallic fragments or transition-metal cations were carefully calculated according to the reactions’ stoichiometry [12,22,30,36,54]. An important aspect in the first stage was to obtain a ligand precipitate of high purity. It took up to five days for some POM crystals to precipitate, the process yields being below 70%. The reaction products were then recrystallized to achieve a desirable purity. The new compound, K_6_[(VO)SiMo_2_W_9_O_39_]·11H_2_O (POM 7), was also synthesized via this method.

For twenty POMs we employed the **“all in one pot” method** based on self-assembling mechanisms. Crystalline powders of different metal ions, e.g., germanium(IV) oxide, vanadyl sulfate, molybdates, vanadates, tungstates, sodium germanate, antimony(III) oxide, were stoichiometricaly mixed with HCl (6 M), the salt of a transition metal cation (TM) if needed, and NaCl (for most POMs, excepting those with vanadyl ions where KCl was added) to produce POM in aqueous solutions [1,14,15,16,22,30] which precipitated in up to two days. The precipitates were then filtered and desiccated, beautifully colored POMs crystals being obtained with reaction yields of 71–84%. Some POMs required recrystallization (in order) to reach the desired purity.

All syntheses of nanoPOMs were detailed in Supplementary Material 3. 

#### 4.1.3. Physico-Chemical Characterizations of Polyoxometalates

The instrumental methods employed in the physico-chemical characterization of the synthesized POMs were elemental chemical analysis, thermogravimetric analysis, electronic spectroscopy in the UV range, FTIR and NMR spectroscopy.

**Elemental chemical analysis** served to determine the composition of the various elements. We determined the presence of P, As, Sb and Si by atomic absorption spectrometry using a Perkin-Elmer 3030 AA spectrophotometer (Perkin-Elmer, Norwalk, CT, USA). For the C, N and H atoms from organic and organometallic fragments, a Vario EL analyzer (Elementar Analysensysysteme GmbH, Hanau, Germany) was employed. The contents of Sn, Mo, V, Sb and W were determined by inductively coupled plasma atomic emission spectroscopy using a RigaKu Spectro CIROS^CCD^ spectrometer (RigaKu Co., Tokyo, Japan). Finally, Na and K were determined by flame photometry with an Eppendorf FEP flame photometer (Eppendorf GmbH, Hamburg, Germany). 

**The thermogravimetric analysis** was conducted to determine the water content (crystallization/lattice water and coordination water molecules) for each POM, using a Mettler-Toledo TG/S DTA 851 thermogravimeter (Mettler-Toledo GmbH, Greifensee, Switzerland) with a platinum crucible, 20 mL/min N_2_ flow, and 5 °C/min heating rate. 

**Electronic spectra in the UV range** were recorded on a Shimadzu Specord UV-VIS-75 (Shimadzu Europe GmbH, Duisburg, Germany), using quartz cells, with a path length of 1 cm. All POM samples were used in 5 × 10^−5^ M aqueous solutions. 

**Vibrational spectroscopy** served to establish the presence of certain bond types in the POMs’ structures. A Jasco 610 FTIR spectrophotometer (Jasco Int. Co Ltd., Gross-Umstadt, Germany) set at a resolution of 0.5 cm^−1^, in the wavenumber range between 7800–350 cm^−1^, was used in the process. The FTIR absorption spectra were recorded in KBr pellets, and all FTIR spectra were analyzed using a Jasco Spectra Manager Version 2.05.03 software (Jasco Int. Co. Ltd., Gross-Umstadt, Germany). 

**NMR spectra** for all POMs with organic or organometallic fragments have been published in our previous work [22]. They were recorded at room temperature, using a Varian Gemini-300 spectrophotometer (Varian Inc. NMR Systems, Palo Alto USA) operating at 300 MHz for ^1^H spectra and at 75.47 MHz for ^13^C spectra, respectively. To record the spectra, each POM was dissolved in CDCl_3_ (solvent), while TMS (Si(CH_3_)_4_) and n-butyltin trichloride (n-C_4_H_9_SnCl_3_) served as standard reference. 

### 4.2. Antimicrobial Activity of Polyoxometalates

#### 4.2.1. Reagents and Materials

Five reference microbial strains, two Gram-positive (*S. aureus* ATCC 6538P, *B. cereus* ATCC 14579) and three Gram-negative (*E. coli* ATCC 10536, *S. enteritidis* ATCC 13076, *P. aeruginosa* ATCC 27853), were used for in vitro susceptibility testing. These reference microbial strains were obtained from the American Type Culture Collection (ATCC, Manassas, VA, USA). In this experiment we used ultrapure water produced by a Millipore Milli-Q50 Ultra-Pure Water System, with 18.00 MΩ·cm (Millipore S.A., Molsheim, France). Sterile media and various consumables were also needed for the antimicrobial characterization of the POMs.

#### 4.2.2. Disk Diffusion Method

The antimicrobial activity of all the synthesized POMs was qualitatively determined using the **disk diffusion susceptibility method**, according to the standards developed by the Clinical and Laboratory Standards Institute [55,56], as previously described in literature [57,58,59], that have been adapted for the purposes of this screening.

For each of the five species an initial suspension of bacterial cultures was inoculated on nutrient agar plates (Merck KGaA, Darmstadt, Germany), incubated for 24 h at 37 ± 2 °C and resuspended in a physiological saline buffer to a 10^6^ CFU/mL concentration (on a 0.5 McFarland scale) and was further inoculated on Muëller Hinton agar plates (Merck KGaA, Darmstadt, Germany). The initial inoculum was similar to that prepared for the classical antibiotic susceptibility test, so POMs’ effects (in sensitivity terms) were comparable to those of the antibiotic tested, i.e., Amoxicillin (25 μg; Oxoid Ltd., Basingstoke, UK).

After inoculation, the medium surface was dried and a number of eight wells were radially drilled 1.5 cm from the outer edge, 3 cm apart. From aliquot samples of 1 mg/mL of each nanocompound dissolved in physiological solution (NaCl 0.15 M) 5 μL were placed in each of the eight wells and let 30 min to diffuse into the agar plates. The plates were then incubated for 24 h at 37 ± 2 °C. Amoxicillin served as a positive control, while the physiological solution (NaCl 0.15 M) was used as a negative control. Readings were conducted by measuring the diameter of the inhibition zone (Halo Zone Test, in mm). All tests were triplicated, and the measured diameters of the inhibition zone were expressed (in mm) as mean ± standard deviation.

In order to observe their stability in physiological solutions and to check the evolution of their antimicrobial activity, the POM solutions were retested six months after their preparation. The diameters of the inhibition zone determined during retesting are highlighted below the initial values in Table 3.

#### 4.2.3. Minimum Inhibitory Concentration (MIC)

In order to quantify their effectiveness, the **Minimum Inhibitory Concentration** of these POMs on bacterial activity was determined using the broth microdilution method described by Quinn et al. [60], Markey et al. [57], the Clinical and Laboratory Standards Institute [61,62], adapted for this experiment.

Testing was conducted on the same Gram-positive and Gram-negative bacteria. Microorganisms’ suspensions in saline buffer (NaCl solution 0.15 M) obtained according to Section 4.2.2 were inoculated. Ten successive dilutions of the POM solutions (1/2 to 1/1024) in nutrient broth (Merck KGaA, Darmstadt, Germany) were performed. As a result, amounts of 2.5 to 0.0048 mg/well of each active POM were placed in sterile microplates (one each for every nanocompound). The microplates thus prepared were incubated at 37 ± 2 °C for 24 h.

Comparisons of the amount of bacterial growth in each well containing POM solutions with the amount of growth in the growth-control wells were performed and the maximal dilution for which the tested POMs inhibited bacterial growth was established.

#### 4.2.4. Minimum Bactericidal Concentration (MBC)

The minimum bactericidal activity (**Minimum Bactericidal Concentration**) of POMs for the five bacterial species described above was established using the microdilution method. 5 μL of POMs solutions from each of the wells where the inhibitory effect was observed were introduced in the same nutrient broth as mentioned above and inoculations on nutrient agar sterile plates (Merck KGaA, Darmstadt, Germany) were performed for similar dilutions. The plates thus prepared were incubated for 24 h at 37 ± 2 °C and bacterial growth was observed. The reading of the results was performed at 24 h by observing the evolution of the bacterial growth on the solid medium. 

Polyoxometalates were classified as bactericidal if they prevented growth on this medium. The minimum bactericidal concentration was determined for the lowest dilution at which bacterial growth was blocked.

## 5. Conclusions

This research achieved its goals to synthesize “smart” nanocompounds based on different structures, to characterize them in terms of structure-property relations, to investigate their molecular mechanisms and to test in vitro their antibacterial effects, in order to formulate and implement potential drugs meant to replace similar products obtained via organic syntheses. Herein, we characterized in terms of chemical structure-antimicrobial activity relationship the following types: a. the Keggin series–polyoxometalates with structures such as saturated/mono- and trilacunary Keggin, mono- and trilacunary *pseudo*-Keggin, saturated/monolacunary Keggin with mixed addenda atoms, trilacunary Keggin/*pseudo*-Keggin by sandwich type; b. one monolacunary Wells-Dawson structure with mixed addenda atoms; c. six clusters. For all active compounds the stability of their antimicrobial effects was also investigated. 

We identified three POMs that presented sound antibacterial activity against all five bacterial strains tested: POMs 26 (a tri-lacunary *pseudo*-Keggin sandwich type), 20 (a butyltin salt cluster) and 28 (a cluster derivatized with organometallic fragments). Of these, only POM 28 has constantly exhibited a stronger effect compared to amoxicillin (including after 6 months retesting, even if its effects were diminished by half). POMs 1, 13, 14 and 30 were effective against four bacterial strains. POMs 2, 4, 7, 8, 23a, 23b, 24a, 24b, 25 and 27 exhibited antibacterial effects against Gram-positive strains, while POMs 18 was effective against one Gram-negative strain, *S. enteritidis*. All other POMs exerting antibacterial effects on Gram-negative strains (1, 11, 13, 14, 15, 17, 19, 20, 26, 28, 29, 30) were active against Gram-positive strains as well. In contrast, nine compounds (3, 5, 6, 9, 10, 12, 16a, 16b, 21 and 22) had no antibacterial actions at all. Presumably higher concentrations of these compounds, closer to the amoxicillin level, would enhance their antibacterial action. New studies in terms of selectivity and specificity of these potential antibacterial agents are required to clarify these extremely important aspects needed to be considered in drug designs. The new compound synthesized and characterized by us, K_6_[(VO)SiMo_2_W_9_O_39_]·11H_2_O (POM 7), a mono-lacunary Keggin with mixed addenda atoms, presented antibacterial activity only against *S. aureus*. These nanocompounds present the disadvantage of being essentially inorganic substances and their toxicity is a matter of concern. Nevertheless, the nano-revolution will inevitably transfer spectacular new technological advances into life sciences. All these novel steps leading to functionalized biocompatible, non-toxic, inorganic compounds, stable in physiological conditions, modelled for exerting maximal antimicrobial effects, can open an alternative approach to the classical treatment of infectious diseases.

A true success in POMs chemistry and pharmacology would lead to the synthesis of pharmaceutical nanocompounds mimicking the behavior of biomacromolecules, with remarkable antibacterial activity effective against certain pathogens with acquired antibiotic resistance, able to regenerate animal and human tissue while annihilating the infection. 

## Figures and Tables

**Table 1:** The structure of all the synthesized polyoxometalates.

POM No.	Chemical Formula of POMs	Structure Types of POMs
**1.**	Na_4_[Fe^III^(H_2_O)PMo_11_O_39_]·18H_2_O	mono-lacunary Keggin
**2.**	Na_9_[Fe_3_(H_2_O)_3_(PMo_9_O_34_)_2_]	tri-lacunary Keggin/sandwich type
**3.**	Na_8_[SiW_11_O_39_]·12H_2_O	mono-lacunary Keggin
**4.**	Na_11_[Fe_3_(H_2_O)_3_(SiW_9_O_34_)_2_]·25H_2_O	tri-lacunary Keggin/sandwich type
**5.**	K_3_[(VO)_3_PMo_9_O_34_]·14H_2_O	tri-lacunary Keggin
**6.**	Na_6_[PMo_9_^VI^V_3_^V^O_40_]·16H_2_O	Keggin with mixed addenda atoms
**7.**	K_6_[(VO)SiMo_2_W_9_O_39_]·11H_2_O	mono-lacunary Keggin with mixed addenda atoms
**8.**	K_10_[(VO)_4_(PW_9_O_34_)_2_]·26H_2_O	tri-lacunary Keggin/sandwich type
**9.**	K_10_[(VO)_4_(AsW_9_O_34_)_2_]·21H_2_O	tri-lacunary *pseudo*-Keggin/sandwich type
**10.**	K_11_H[(VO)_3_(Sb^III^W_9_O_33_)_2_]·27H_2_O	tri-lacunary *pseudo*-Keggin/sandwich type
**11.**	Na_12_[Sb_2_W_22_O_74_(OH)_2_]⋅38H_2_O	cluster
**12.**	H_4_[SiW_12_O_40_]·14H_2_O	saturated Keggin
**13.**	H_3_[PW_12_O_40_]·12H_2_O	saturated Keggin
**14.**	H_3_[PMo_12_O_40_]·13H_2_O	saturated Keggin
**15.**	Na_9_[SbW_9_O_33_]·19,5H_2_O	tri-lacunary *pseudo*-Keggin
**16a.**	Na_10_[SiW_9_O_34_]·24H_2_O	tri-lacunary Keggin
**16b.**	Na_10_[SiW_9_O_34_]·24H_2_O–recryst.	tri-lacunary Keggin
**17.**	Na_27_[NaAs_4_W_40_O_140_]·42H_2_O	cluster
**18.**	Na_8_H[PW_9_O_34_]·20H_2_O	tri-lacunary Keggin
**19.**	(NBu_4_)_27_[NaAs_4_Mo_40_O_140_] ·12H_2_O	cluster
**20.**	(Bu_3_Sn)_18_[NaSb_9_W_21_O_86_]	cluster
**21.**	K_6_[Co(H_2_O)SiMo_2_W_9_O_39_]·14H_2_O	mono-lacunary Keggin with mixed addenda atoms
**22.**	K_10_[Co(H_2_O)Si_2_MoW_16_O_61_]·18H_2_O	mono-lacunary Wells-Dawson with mixed addenda atoms
**23a.**	Na_5_[Fe^III^(H_2_O)SiW_11_O_39_]·24H_2_O	mono-lacunary Keggin
**23b.**	Na_5_[Fe^III^(H_2_O)SiW_11_O_39_]·24H_2_O–recryst.	mono-lacunary Keggin
**24a.**	Na_5_[Fe^III^(H_2_O)GeW_11_O_39_]·26H_2_O	mono-lacunary Keggin
**24b.**	Na_5_[Fe^III^(H_2_O)GeW_11_O_39_]·26H_2_O–recryst.	mono-lacunary Keggin
**25.**	Na_10_[Mn_4_(H_2_O)_2_(AsW_9_O_34_)_2_]·27H_2_O	tri-lacunary *pseudo*-Keggin/sandwich type
**26.**	Na_12_[Co_3_(H_2_O)_3_(BiW_9_O_33_)_2_]·37H_2_O	tri-lacunary *pseudo*-Keggin/sandwich type
**27.**	Na_14_[Mn_3_(H_2_O)_3_(SiW_9_O_34_)_2_]·28H_2_O	tri-lacunary Keggin/sandwich type
**28.**	(NH_4_)_4_(NBu_4_)_5_[Na(BuSn)_3_Sb_9_W_21_O_86_]·17H_2_O	cluster
**29.**	K_27_[NaAs_4_W_40_O_140_]·52H_2_O	cluster
**30.**	K_6_[SiV^IV^W_11_O_40_]·12H_2_O	mono-lacunary Keggin

**Table 2:** Physico-chemical data of polyoxometalates.

POM No.	Elemental Analysis and TG Data (Found (Calcd.))	UV (H_2_O) Data (nm/cm^−1^): ν_2_(M=O_t_) and ν_1_(M-O_c,e_-M)	FTIR Spectral Data (ν_max_ (cm^−1^) and Their Contribution in the POMs’ Structure)
**1.**	M = 2200.38; Na (4.20 (4.18)); Fe (2.58 (2.54)); P (1.39 (1.41)); Mo (47.98 (47.96)); H_2_O (15.62 (15.55)).	ν_2_ = 210/47,619 and ν_1_ = 228/43,859.	1128 (w, ν_as_(P-O_i_)); 1049 (sh, ν_as_(P-O_i_)); 924 (vs, sh, ν_as_(Mo=O_t_)); 887 (vs, ν_as_(Mo-O_c_-Mo)); 847 (s, sp ν_as_(Mo-O_e_-Mo)); 658 (s, br, ν_as_(Mo-O_e_-Mo)); 621 (s, br, δ(P-O_i_)); 577 (s, br, δ(P-O_i_)); 546 (m, sh, δ(Mo-O-Mo)); 486 (m, ν(Fe-O)).
**2.**	M = 3737.68; Na (5.57 (5.54)); Fe (4.50 (4.48)); P (1.63 (1.66)); Mo (46.24 (46.20)); H_2_O (13.10 (13.01)).	ν_2_ = 219/45,662 and ν_1_ = 271/36,900.	1180–1044 (s, sp, ν_as_(P-O_i_)); 997 (vs, sp, ν_as_(Mo=O_t_)); 978 (vs, sp, ν_as_(Mo-O_c_-Mo)); 775 (m, b ν_as_(Mo-O_e_-Mo)); 667 (w, ν_as_(Mo-O_b_-Mo)/sandwich); 514 (m, sp, δ(Mo-O-Mo)).
**3.**	M = 3074.40; Na (6.04 (5.98)); Si (0.88 (0.91)); W (63.58 (65.78)); H_2_O (7.10 (7.03)).	ν_2_ = 206/48,544 and ν_1_ = 258/38,759.	3446 (vs, br, ν_as_(O-H)); 1635 (w, δ(O-H)); 1005 (vw, sh, ν_as_(Si-O_i_)); 962 (s, sp, ν_as_(W=O_t_)); 910 (vs, sp, ν_as_(W-O_c_-W)); 798 (vs, br, ν_as_(W-O_e_-W)); 517 (vs, br, δ(W-O_c,e_-W)).
**4.**	M = 5378.10; Na (4.72 (4.70)); Fe (3.15 (3.12)); Si (1.02 (1.04)); W (61.58 (61.53)); H_2_O (9.41 (9.38)).	ν_2_ = 200/50,000 and ν_1_ = 257/38,911.	1190–1063 (w, sp, ν_as_(Si-O_i_)); 964 (vs, sp, ν_as_(W=O_t_)); 910 (vs, sp, ν_as_(W-O_c_-W)); 879 (m, sh, ν_as_(W-O_c_-W)); 787 (vs, vbr, ν_as_(W-O_e_-W)); 708 (m, sh, ν(W-O_b_-W)/sandwich); 542 (vw, br, δ( W-O_c,e_-W)); 499 (m, sp, ν(Fe-O)); 403 (m, sp, ν(Fe-O)).
**5.**	M = 2008.74; K (5.87 (5.84)); V (7.64 (7.61)); P (1.51 (1.54)); Mo (43.05 (42.99)); H_2_O (12.62 (12.56)).	ν_2_ = 218/45,871 and ν_1_ = 305/32,787.	1180–1088 (s, sp, ν_as_(P-O_i_)); 989 (vs, sp, ν_as_(V=O_t_)); 941 (vs, sp, ν_as_(Mo=O_t_)); 879 (s, br, ν_as_(Mo-O_c_-Mo)); 796 (m, br, ν_as_(Mo-O_e_-Mo)); 726 (m, ν_as_(Mo-O_e_-Mo)); 625 (s, sp, δ(M-O-M)); 513 (w, sp, δ(Mo-O_c,e_-Mo)).
**6.**	M = 2113.42; Na (6.56 (6.53)); P (1.45 (1.47)); Mo (40.92 (40.86)); V (7.26 (7.23)); H_2_O (13.70 (13.64)).	ν_2_ = 221/45,249 and ν_1_ = 305/32,786.	1190–1063 (vs, sp, ν_as_(P-O_i_)); 989 (m, sh, ν_as_(V=O_t_)); 962 (vs, sp, ν_as_(Mo=O_t_)); 866 (vs, br, ν_as_(V-O_c_-V)+ ν_as_(Mo-O_c_-Mo)); 785 (vs, vbr, ν_as_(V-O_e_-V)+ ν_as_(Mo-O_e_-Mo)); 619 (vs, sp, δ(P-O_i_); 519 (vw, br, δ(V-O_c,e_-V) + δ(Mo-O_c,e_-Mo).
**7.**	M = 2998.20; K (7.84 (7.82)); V (1.73 (1.70)); Si (0.91 (0.94)); Mo (6.44 (6.40)); W (55.26 (55.19)); H_2_O (6.62 (6.61)).	ν_2_ = 199/50,251 and ν_1_ = 258/38,759.	1109 (w, ν_as_(Si-O_i_)); 968 (s, ν_as_(W=O_t_) + ν_as_(Mo=O_t_)); 906 (vs, ν_as_(W-O_c_-W) + ν_as_(Mo-O_c_-Mo)); 783 (vs, ν_as_(W-O_e_-W) + (Mo-O_e_-Mo)); 669 (m, ν_as_(W-O_e_-W)+ ν_as_(Mo-O_e_-Mo)).
**8.**	M = 5586.17; K (7.03 (6.99)); V (3.68 (3.65)); P (1.08 (1.11)); W (59.28 (59.24)); H_2_O (8.45 (8.38)).	ν_2_ = 201/49,751 and ν_1_ = 248/40,323.	3437 (vs, br, ν_as_(O-H)); 1624 (m, sp, δ(O-H)); 1186 (vs, sp, ν_as_(P-O_i_)); 1103 (vs, sp, ν_as_(P-O_i_)); 987 (sh, ν_as_(W=O_t_)); 968 (vs, br, ν_as_(W=O_t_)); 891 (s, br, ν_as_(W-O_c_-W)); 850 (sh, ν_as_(W-O_c_-W)); 791 (vs, vbr, ν_as_(W-O_e_-W)); 719 (s, sp, ν(V-O_b_-W)); 619 (vs, sp, ν_s_(P-O_i_)); 514 (m, br, δ(W-O-W)); 463 (m, br, δ(W-O-W)).
**9.**	M = 5583.99; K (7.05 (7.00)); V (3.66 (3.65)); As (2.65 (2.68)); W (59.29 (59.26)); H_2_O (6.81 (6.78)).	ν_2_ = 201/49,751 and ν_1_ = 256/39,062.	3419 (vs, br, ν_as_(O-H)); 1626 (m, sp, δ(O-H)); 1045 (sh, ν_as_(As-O_i_)); 931 (vs, br, ν_as_(W=O_t_)); 874 (s, sp, ν_as_(W-O_c_-W)); 831 (m, ν_as_(W-O_c_-W)); 796 (m, sp, ν_as_(W-O_e_-W)); 712 (s, sh, ν(V-O_b_-W)/sandwich); 621 (m, br, δ(W-O-W)); 553 (m, br, δ(W-O-W)).
**10.**	M = 5726.92; K (7.55 (7.51)); V (2.70 (2.67)); Sb (4.22 (4.25)); W (57.83 (57.78)); H_2_O (8.55 (8.49)).	ν_2_ = 202/49,505 and ν_1_ = 251/39,841.	3423 (vs, br, ν_as_(O-H)); 1697 (m, δ(H-O-H)); 1667 (m, br, δ(H-O-H)); 995 (m, sp, ν_as_(V=O_t_)); 930 (m, sp, ν_as_(W=O_t_)); 857 (s, sp, ν_as_(W-O_c_-W)); 833 (vs, ν_as_(W-O_c_-W)); 743 (m, sp, ν_as_(Sb-O_i_)); 697 (s, ν_as_(W-O_e_-W)); 553 (m, br, δ(W-O_c,e_-W)).
**11.**	M = 6466.43; Na (4.31 (4.27)); Sb (3.75 (3.77)); W (62.59 (62.55)); H_2_O (10.65 (10.59)).	ν_2_ = 200/50,000 and ν_1_ = 255/39,216.	3332 (vs, br, ν_as_(O-H)); 1619 (m, sp, δ(H-O-H)); 1385 (s, sp ν_as_(NO_3_^−^)); 943 (vs, sp, ν_as_(W=O_t_)); 887 (vs, ν_as_(W-O_c_-W)); 864 (s, sh ν_as_(Sb-O_i_)); 837 (vs, ν_as_(W-O_c_-W)); 800 (s, sh ν_as_(W-O_e_-W)); 771 (vs, br, ν_as_(W-O_e_-W)); 673 (s, br, ν_as_(W-O_b_-W) +δ(O-Sb-O)); 507 (w, br, δ(W-O_c,e_-W)).
**12.**	M = 3130.39; Si (0.88 (0.90)); W (70.52 (70.47)); H_2_O (8.11 (8.06)).	ν_2_ = 207/48,309 and ν_1_ = 263/38,023.	1020 (m, sh, ν_as_(Si-O_i_)); 982 (s, ν_as_(W=O_t_)); 926 (vs, sp, ν_s_(Si-O_i_)); 883 (m, sp, ν_as_(W-O_c_-W)); 787 (vs, br, ν_as_(W-O_e_-W)); 538 (m, δ(W-O-W)).
**13.**	M = 3096.24; P (0.98 (1.00)); W (71.28 (71.25)); H_2_O (7.00 (6.98)).	ν_2_ = 201/49,751 and ν_1_ = 248/40,323.	1080 (vs, sp, ν_as_(P-O_i_); 984 (vs, ν_as_(W=O_t_)); 889 (vs, sp, ν_as_(W-O_c_-W)); 808 (vs, sp, ν_as_(W-O_e_-W)); 596 (w, sp, δ(W-O_c_-W)); 525 (m, δ(W-O_e_-W)).
**14.**	M = 2059.45; P (1.48 (1.50)); Mo (55.93 (55.90)); H_2_O (11.40 (11.37)).	ν_2_ = 193/51,550 and ν_1_ = 270/37,000.	1065 (vs, sp, ν_as_(P-O_i_); 962 (vs, sp, ν_as_(Mo=O_t_)); 870 (s, vbr, ν_as_(Mo-O_c_-Mo)); 787 (vs, br, ν_as_(Mo-O_e_-Mo)); 595 (w, δ(Mo-O-Mo)); 509 (vw, δ(Mo-O-Mo)).
**15.**	M = 2862.51; Na (7.26 (7.23)); Sb (4.22 (4.25)); W (57.87 (57.80)); H_2_O (12.33 (12.27)).	ν_2_ = 207/48,309 and ν_1_ = 238/42,017.	920 (vs, sp, ν_as_(W=O_t_)); 890 (vs, sp, ν_as_(W-O_c_-W)); 767 (s, ν_as_(W-O_e_-W)); 743 (s, sp, ν_as_(Sb-O_i_); 715 (s, ν_as_(W-O_e_-W)); 505 (w, br, δ(W-O-W)).
**16a.**	M = 2888.89; Na (7.98 (7.96)); Si (0.96 (0.97)); W (57.29 (57.27)); H_2_O (15.01 (14.97)).	ν_2_ = 208/48,077 and ν_1_ = 265/37,736.	1635 (m, δ(O-H)); 987 (m, sp, ν_as_(W=O_t_)); 937 (s, ν_as_(Si-O_i_)); 878 (vs, ν_as_(W-O_c_-W)); 844 (vs, ν_as_(W-O_c_-W)); 810 (vs, ν_as_(W-O_e_-W)); 723 (s, ν_as_(W-O_e_-W)); 618 (s, ν_s_(Si-O_i_)); 528 (m, δ(W-O-W)).
**16b.**	M = 2888.89; Na (7.98 (7.96)); Si (0.96 (0.97)); W (57.29 (57.27)); H_2_O (14.91 (14.97)).	ν_2_ = 208/48,077 and ν_1_ = 265/37,736.	1635 (m, δ(O-H)); 987 (m, sp, ν_as_(W=O_t_)); 937 (s, ν_as_(Si-O_i_)); 878 (vs, ν_as_(W-O_c_-W)); 844 (vs, ν_as_(W-O_c_-W)); 810 (vs, ν_as_(W-O_e_-W)); 723 (s, ν_as_(W-O_e_-W)); 618 (s, ν_s_(Si-O_i_)); 528 (m, δ(W-O-W)).
**17.**	M = 11293.56; Na (5.73 (5.70)); As (2.63 (2.65)); W (65.15 (65.11)); H_2_O (6.75 (6.70)).	ν_2_ = 200/50,000 and ν_1_ = 243/41,152.	951 (vs, sp, ν_as_(W=O_t_)); 876 (vs, b ν_as_(As-O_i_)+ν_as_(W-O_c_-W)); 793 (vs, sp ν_as_(W-O_c_-W)); 710 (vs, b ν_as_(W-O_e_-W)); 634 (s, b ν_s_(As-O_i_)); 577 (m, b, δ(W-O-W)).
**18.**	M = 2774.75; Na (6.65 (6.63)); P (1.10 (1.12)); W (59.68 (59.63)); H_2_O (13.05 (12.99)).	ν_2_ = 208/48,077 and ν_1_ = 245/40,816.	1054 (s, sp, ν_as_(P-O_i_); 1014 (w, ν_as_(P-O_i_); 937 (vs, sp, ν_as_(W=O_t_)); 881 (vs, sp, ν_as_(W-O_c_-W)); 741 (vs, b, ν_as_(W-O_e_-W)); 503 (vw, b, δ(W-O-W)).
**19.**	M = 13162.90; Na (0.20 (0.17)); C (39.46 (39.42)); H (7.66 (7.63)); N (2.88 (2.87)); As (2.26 (2.28)); Mo (29.21 (29.15)); H_2_O (1.67 (1.64)).	ν_2_ = 209/47,847 and ν_1_ = 228/43,859.	3446 (vs, br, ν_as_(O-H)); > 2800 (vs, br, ν_as_(C-H)); 1483 (vs, br, ν_as_(C-N)); 1617 (w, b, δ(H-O)); 943 (vs, sh, ν_as_(Mo=O_t_)); 924 (vs, sp, ν_as_(Mo=O_t_)); 904 (vs, sp, ν_as_(As-O_i_)+ν_as_(Mo-O_c_-Mo)); 879 (s, sh, ν_as_(Mo-O_c_-Mo)); 854 (vs, ν_as_(Mo-O_c_-Mo)); 806 (vs, b, ν_as_(Mo-O_e_-Mo)); 764 (vs, sh, ν_as_(Mo-O_e_-Mo)); 735 (vs, sh, ν_as_(Mo-O_e_-Mo)); 706 (vs, b, ν_as_(Mo-O_e_-Mo)); 663 (s, ν_s_(As-O_i_)); 584 (m, δ(Mo-O-Mo)); 557 (w, b, δ(Mo-O-Mo)); 517 (w, b, δ(Mo-O-Mo)).
**20.**	M = 11576.37; Na (0.22 (0.20)); C (22.44 (22.41)); H (4.25 (4.23)); Sn (18.48 (18.46)); Sb (9.45 (9.47)); W (33.39 (33.35)).	ν_2_ = 200/50,000 and ν_1_ = 254/39,370.	949 (vs, sp, ν_as_(W=O_t_)); 862 (s, b, ν_as_(Sb-O_i_) + ν_as_(W-O_c_-W)); 796 (s, ν_as_(W-O_e_-W)); 739 (vs, ν_as_(W-O_e_-W)); 749 (vs, ν_as_(W-O_e_-W)); 657 (s, δ(Sb-O_i_)); 577 (w, ν_as_(Sb-O_i_)); 505 (w, ν(C-Sn-O)); 493 (w, δ(Sb-O)); the presence of bands due to the stretching and deformation vibrations of the C-H and C-C bonds of the butyl groups in the ranges 1000–1300, 1700–1950 and >2800 cm^−1^ is also observed in the spectrum.
**21.**	M = 3062.25; K (7.70 (7.66)); Co (1.94 (1.92)); Si (0.90 (0.92)); Mo (6.30 (6.27)); W (54.08 (54.03)); H_2_O (8.87 (8.82)).	ν_2_ = 203/49,261 and ν_1_ = 253/39,526.	995 (s, sp, ν_as_(Si-O_i_); 953 (vs, sp, ν_as_(Mo=O_t_)); 901 (vs, sp, ν_as_(W=O_t_)); 798 (vs, b, ν_as_(Mo-O_c_-Mo)+ν_as_(W-O_c_-W)); 739 (vs, b, ν_as_(Mo-O_e_-Mo)+ν_as_(W-O_e_-W)); 704 (s, vb, ν_as_(Mo-O_e_-Mo)+ν_as_(W-O_e_-W)); 538 (m, sh, δ(W-O-W)); 524 (m, b, δ(W-O-W)) + δ(Mo-O-Mo)); 482 (m, sh δ(W-O)).
**22.**	M = 4861.72; K (8.08 (8.04)); Co (1.24 (1.21)); Si (1.14 (1.16)); Mo (1.98 (1.97)); W (60.53 (60.50)); H_2_O (7.10 (7.04)).	ν_2_ = 203/49,261 and ν_1_ = 253/39,526.	995 (sh, sp, ν_as_(Si-O_i_)); 952 (vs, sp, ν_as_(Mo=O_t_)); 901 (vs, b ν_as_(W=O_t_)); 798 (s, b ν_as_(W-O_c_-W) + ν_as_(Mo-O_c_-Mo)); 739 (vs, b ν_as_(W-O_c_-W)); 704 (s, ν_as_(W-O_e_-W)); 525 (s, b, δ(W-O-W) + δ(Mo-O-Mo));); 482 (sh, b ν_s_(W-O_c_-Co) + ν_s_(Mo-O_c_-Co)).
**23a.**	M = 3295.48; Na (3.50 (3.49)); Fe (1.70 (1.69)); Si (0.82 (0.85)); W (61.38 (61.36)); H_2_O (13.68 (13.67)).	ν_2_ = 200/50,000 and ν_1_ = 259/38,610.	1088 (m, ν_as_(Si-O_i_); 1005 (sh, ν_as_(Si-O_i_); 964 (s, ν_as_(W=O_t_)); 910 (vs, b, ν_s_(Si-O_i_)+ν_as_(W-O_c_-W)); 876 (sh, ν_as_(W-O_c_-W)); 787 (vs, b, ν_as_(W-O_e_-W)); 704 (sh, ν_as_(W-O_e_-W)); 538 (m, δ(W-O_c_-W)); 519 (m, b, δ(W-O_e_-W)); 418 (m, sh, ν(Fe-O)).
**23b.**	M = 3295.48; Na (3.50 (3.49)); Fe (1.70 (1.69)); Si (0.82 (0.85)); W (61.38 (61.36)); H_2_O (13.58 (13.67)).	ν_2_ = 200/50,000 and ν_1_ = 259/38,610.	1088 (m, ν_as_(Si-O_i_); 1005 (sh, ν_as_(Si-O_i_); 964 (s, ν_as_(W=O_t_)); 910 (vs, b, ν_s_(Si-O_i_)+ν_as_(W-O_c_-W)); 876 (sh, ν_as_(W-O_c_-W)); 787 (vs, b, ν_as_(W-O_e_-W)); 704 (sh, ν_as_(W-O_e_-W)); 538 (m, δ(W-O_c_-W)); 519 (m, b, δ(W-O_e_-W)); 418 (m, sh, ν(Fe-O)).
**24a.**	M = 3376.06; Na (3.42 (3.40)); Fe (1.67 (1.65)); Ge (2.12 (2.15)); W (59.92 (59.90)); H_2_O (14.42 (14.41)).	ν_2_ = 202/49,505 and ν_1_ = 255/39,216.	982 (vs, sp ν_as_(W=O_t_)); 903 (vs, sh, ν_as_(W-O_c_-W)); 876 (vs, b, ν_as_(W-O_c_-W)); 814 (s, sh, ν_as_(Ge-O) + ν_as_(W-O_e_-W)); 771 (vs, b, ν_as_(Ge-O_i_) + ν_as_(W-O_e_-W)); 525 (w, b, δ(W-O_c,e_-W)).
**24b.**	M = 3376.06; Na (3.42 (3.40)); Fe (1.67 (1.65)); Ge (2.12 (2.15)); W (59.92 (59.90)); H_2_O (14.38 (14.41)).	ν_2_ = 202/49,505 and ν_1_ = 255/39,216	982 (vs, sp ν_as_(W=O_t_)); 903 (vs, sh, ν_as_(W-O_c_-W)); 876 (vs, b, ν_as_(W-O_c_-W)); 814 (s, sh, ν_as_(Ge-O) + ν_as_(W-O_e_-W)); 771 (vs, b, ν_as_(Ge-O_i_) + ν_as_(W-O_e_-W)); 525 (w, b, δ(W-O_c,e_-W))
**25.**	M = 5519.02; Na (4.18 (4.17)); Mn (3.99 (3.98)); As (2.68 (2.72)); W (59.98 (59.96)); H_2_O (9.51 (9.47)).	ν_2_ = 201/49,751 and ν_1_ = 248/40,323.	3421 (vs, b, ν_as_(O-H)); 1624 (vs, sp, δ(H-O-H)); 957 (vs, sp, ν_as_(W=O_t_)); 877 (vs, b ν_as_(As-O_i_)+ν_as_(W-O_c_-W)); 839 (s, sp, ν_as_(W-O_c_-W)); 768 (vs, ν_as_(W-O_e_-W)); 712 (s, ν_as_(W-O_e_-W)+ν_as_(W-O_b_-W)/sandwich); <514 (m, b, δ(W-O-W)).
**26.**	M = 5956.33; Na (4.66 (4.63)); Co (2.98 (2.97)); Bi (7.00 (7.02)); W (55.60 (55.56)); H_2_O (12.15 (12.10)).	ν_2_ = 194/51,500 and ν_1_ = 256/38,991.	946 (s, ν_as_(W=O_t_)); 867 (vs, vb, ν_as_(W-O_c_-W)); 839 (s, sp, ν_as_(Bi-O_i_)); 795 (vs, ν_as_(W-O_e_-W)); 740 (s, b, ν_as_(W-O_e_-W)); 740 (s, b, ν_as_(W-O_b_-W)); 508 (w, δ(W-O-W)).
**27.**	M = 5498.39; Na (5.88 (5.85)); Mn (3.05 (3.00)); Si (1.00 (1.02)); W (60.25 (60.18)); H_2_O (10.12 (10.16)).	ν_2_ = 213/46,948 and ν_1_ = 256/39,066.	1631 (m, δ(H_2_O)); 1568 (m, δ(H_2_O)); 987 (m, ν_as_(W=O_t_)); 940 (s, ν_as_(Si-O_i_)); 893 (vs, ν_as_(W-O_c_-W)); 807 (vs, ν_as_(W-O_e_-W)); 722 (s, ν_as_(W-O_e_-W)); 682 (s, ν_s_(Si-O_i_-W)); 519 (vw, δ(W-O_c,e_-W)); 350 (s, ν(Mn-O_c,e_-W)).
**28.**	M = 8473.62; C (13.06 (13.04)); H (3.10 (3.06)); N (1.54 (1.49)); Na (0.28 (0.27)); Sb (12.94 (12.93)); Sn (4.35 (4.20)); W (45.61 (45.56)); H_2_O (3.64 (3.61)).	ν_2_ = 191/52,356 and ν_1_ = 275/36,363.	3485 (s, ν_as_(hydrogen bond from lattice water)); 3373 (vs, ν_as_(hydrogen bond from lattice water)); 3171 (m, b, ν(N-H) from NH_4_^+^); 1648 (m, δ(O-H)); 1621 (sh, δ(O-H)); 1404 (s, δ(N-H) from NH_4_^+^); 1293 (m, ν_as_(C-N) from NBu_4_); 958 (s, ν_as_(W=O_t_)); 927 (m, ν_as_(W=O_t_)); 881 (s, ν_as_(W-O_c_-W)); 871 (s, ν_as_(W-O_c_-W)); 851 (s, ν_as_(W-O_c_-W)); 800 (vs, ν_as_(W-O_e_-W)); 766 (vs, ν_as_(W-O_e_-W)); 701(sh, ν_as_(C-N) from NBu_4_); 681 (s, ν_as_(Sb-O_i_) + ν_as_(Sn-O) + ν(C-Sn-O)); 613 (m, ν_as_(Sb-O_i_) + ν_as_(Sn-O) + ν(C-Sn-O)); 549 (s, ν_as_(Sb-O_i_) + ν_as_(Sn-O) + ν(C-Sn-O)); 489 (m, ν_as_(Sn-C) + δ(W-O-W)); 431 (w, δ(Sb-O)); 418 (m, ν_as_(Sn-C)).
**29.**	M = 11908.64; K (8.90 (8.86)); Na (0.21 (0.19)); As (2.50 (2.52)); W (61.81 (61.75)); H_2_O (7.92 (7.87)).	ν_2_ = 201/49,751 and ν_1_ = 254/39,370.	966 (vs, sp, ν_as_(W=O_t_)); 883 (vs, b, ν_as_(As-O_i_)+(W-O_c_-W)); 783 (vs, b, ν_as_(W-O_e_-W)); 733 (s, sh, ν_as_(W-O_e_-W)); 671 (vs, b, ν_as_(As-O_i_)); 553 (m, b, δ(W-O-W)).
**30.**	M = 3192.02; K (7.38 (7.35)); Si (0.86 (0.88)); V (1.62 (1.60)); W (63.39 (63.35)); H_2_O (6.62 (6.77)).	ν_2_ = 198/50,505 and ν_1_ = 257/38,910.	1054 (w, sp, ν_as_(Si-O_i_)); 1000 (w, sp, ν_s_(Si-O_i_)); 965 (s, sp, ν_as_ (W=O_t_)); 989 (m, sp, ν_as_ (V=O)); 884 (vs, ν_as_(W-O_c_-W)); 805 (vs, ν_as_ (W-O_e_-W)); 741 (vs, vb, ν_as_ (W-O_e_-W)); 661 (m, δ(O_i_-Si-O_i_)); 518 (w, δ(W-O_c,e_-W)).

**Table 3:** POM antibacterial activity as measured by the disk diffusion method.

POM No.	Effect of POMs on Microorganisms (Halo Zone Test/mm)				
	*S. aureus*	*B. cereus*	*S. enteritidis*	*E. coli*	*P. aeruginosa*
**1.**	12 ± 0.50 R ^1^	7 ± 0.30 7 ± 0.22 ^2^	6 ± 0.24 R	R	9 ± 0.22 R
**2.**	R	12 ± 0.30 12 ± 0.44	R	R	R
**3.**	R	R	R	R	R
**4.**	8 ± 0.23 R	7 ± 0.45 R	R	R	R
**5.**	R	R	R	R	R
**6.**	R	R	R	R	R
**7.**	15 ± 0.50 13 ± 0.50	R	R	R	R
**8.**	10 ± 0.50 R	10 ± 0.20 R	R	R	R
**9.**	R	R	R	R	R
**10.**	R	R	R	R	R
**11.**	11 ± 0.55 R	R	10 ± 0.20 R	R	R
**12.**	R	R	R	R	R
**13.**	8 ± 0.12 12 ± 0.5	8 ± 0.22 7 ± 0.25	10 ± 0.50 10 ± 0.22	10 ± 0.50 12 ± 0.25	R
**14.**	8 ± 0.22 7 ± 0.25	R	12 ± 0.25 6 ± 0.32	12 ± 0.35 8 ± 0.25	12 ± 0.50 8 ± 0.42
**15.**	32 ± 0.22 18 ± 0.50	23 ± 0.25 12 ± 0.50	26 ± 0.25 12 ± 0.50	R	R
**16a. ^3^**	R	R	R	R	R
**16b. ^4^**	R	R	R	R	R
**17.**	R	10 ± 0.25 R	18 ± 0.25 10 ± 0.50	R	R
**18.**	R	R	8 ± 0.22 R	R	R
**19.**	20 ± 0.50 12 ± 0.30	14 ± 0.50 8 ± 0.65	25 ± 0.23 19 ± 0.18	R R	R R
**20.**	30 ± 0.10 13 ± 0.25	24 ± 0.15 14 ± 0.22	22 ± 0.10 10 ± 0.22	12 ± 0.25 8 ± 0.25	12 ± 0.22 18 ± 0.25
**21.**	R	R	R	R	R
**22.**	R	R	R	R	R
**23a. ^3^**	14 ± 0.25 13 ± 0.25	R	R	R	R
**23b. ^4^**	14 ± 0.22 13 ± 0.12	R	R	R	R
**24a. ^3^**	12 ± 0.15 10 ± 0.25	R	R	R	R
**24b. ^4^**	10 ± 0.25 R	R	R	R	R
**25.**	13 ± 0.25 16 ± 0.55	R	R	R	R
**26.**	18 ± 0.55 16 ± 0.10	20 ± 0.55 12 ± 0.15	18 ± 0.55 15 ± 0.15	16 ± 0.25 14 ± 0.22	15 ± 0.25 22 ± 0.50
**27.**	14 ± 0.50 10 ± 0.35	14 ± 0.37 10 ± 0.22	R	R	R
**28.**	40 ± 0.50 20 ± 0.22	30 ± 0.50 12 ± 0.55	30 ± 0.52 23 ± 0.23	20 ± 0.23 16 ± 0.27	16 ± 0.45 8 ± 0.56
**29**	12 ± 0.50 12 ± 0.22	R	18 ± 0.50 R	R	R
**30**	18 ± 0.55 11 ± 0.25	6 ± 0.51 6 ± 0.45	12 ± 0.56 7 ± 0.52	14 ± 0.57 8 ± 0.45	R
**+ive C ^5^**	19 ± 0.52	12 ± 0.37	18 ± 0.33	18 ± 0.26	R
**−ive C ^6^**	R	R	R	R	R

^1^ R = resistant; ^2^ the retest values of the halo zone test (from the same solution, 6 months after the initial preparation) are written in the second row for each POM; ^3^ only POMs 16, 23 and 24 required recrystallizations: POM (number) a = original POM, ^4^ POM (number) b = recrystallized POM; ^5^ +ive C = positive control (Amoxicillin, 25 μg); ^6^ −ive C = negative control (0.15 M NaCl solution).

**Table 4:** Minimum inhibitory concentration of active POMs.

POM No.	Minimum Inhibitory Concentration (mg/L)				
	*S. aureus*	*B. cereus*	*S. enteritidis*	*E. coli*	*P. aeruginosa*
**1.**	0.625	1.25	1.25	-	0.625
**2.**	-	1.25	-	-	-
**4.**	0.625	1.25	-	-	-
**7.**	1.25	-	-	-	-
**8.**	1.25	1.25	-	-	-
**11.**	0.039	-	0.156	-	-
**13.**	1.25	2.5	0.312	0.078	-
**14.**	0.156	-	0.312	0.156	0.312
**15.**	1.25	0.312	0.625	-	-
**17.**	-	0.625	1.25	-	-
**18.**	-	-	1.25	-	-
**19.**	0.156	0.625	0.312	-	-
**20.**	0.039	0.039	0.156	0.156	0.625
**23a.**	0.078	-	-	-	-
**23b.**	0.625	-	-	-	-
**24a.**	0.078	-	-	-	-
**24b.**	0.625	-	-	-	-
**25.**	0.156	-	-	-	-
**26.**	0.312	0.078	0.156	0.312	0.625
**27.**	0.156	0.156	-	-	-
**28.**	0.625	0.0048	0.019	0.078	0.039
**29.**	0.625	-	0.625	-	-
**30.**	0.078	0.312	0.312	0.156	-

**Table 5:** Minimum bactericidal concentration of active POMs.

POM no.	Minimum Bactericidal Concentration (mg/L)				
	*S. aureus*	*B. cereus*	*S. enteritidis*	*E. coli*	*P. aeruginosa*
**1.**	1.25	2.5	-	-	-
**2.**	-	-	-	-	-
**4.**	2.5	2.5	-	-	-
**7.**	1.25	-	-	-	-
**8.**	2.5	2.5	-	-	-
**11.**	1.25	-	1.25	-	-
**13.**	0.625	-	1.25	1.25	-
**14.**	0.625	-	1.25	1.25	0.625
**15.**	2.5	0.625	0.625	-	-
**17.**	-	1.25	2.5	-	-
**18.**	-	-	2.5	-	-
**19.**	1.25	1.25	0.625	-	-
**20.**	1.25	0.625	2.5	1.25	2.5
**23a.**	1.25	-	-	-	-
**23b.**	1.25	-	-	-	-
**24a.**	1.25	-	-	-	-
**24b.**	1.25	-	-	-	-
**25.**	2.5	-	-	-	-
**26.**	2.5	0.625	1.25	1.25	1.25
**27.**	1.25	0.625	-	-	-
**28.**	0.625	0.312	0.625	1.25	0.625
**29.**	2.5	-	0.625	-	-
**30.**	0.625	0.625	0.312	1.25	-

## Data Availability

Data is contained within the article and all three Supplementary Materials.

## Supplementary Materials

The following are available online at https://www.mdpi.com/article/10.3390/ph15010033/s1, In Supplementary Material 1, the synthesis method and physico-chemical characterization of K_6_[(VO)SiMo_2_W_9_O_39_]·11H_2_O (POM 7) and its ligand K_8_[SiMo_2_W_9_O_39_]·14H_2_O (L 7); Figure S1: TG/DTG/DTA curves of K_6_[(VO)SiMo_2_W_9_O_39_]·11H_2_O; Figure S2: The proposed structure for POM 7: K_6_[(VO)SiMo_2_W_9_O_39_]·11H_2_O; Figure S3: FTIR spectra of POM 7, K_6_[(VO)SiMo_2_W_9_O_39_]·11H_2_O, and his ligand (L7), K_8_[SiMo_2_W_9_O_39_]·14H_2_O; Figure S4: UV (upper right window) and VIS electronic spectra of K_6_[(VO)SiMo_2_W_9_O_39_]·11H_2_O; Figure S5: ESR spectrum of K_6_[(VO)SiMo_2_W_9_O_39_]·11H_2_O complex powder at room temperature (solid line) and its simulated spectrum (dashed line); Table S1. FTIR spectral data (M = metal atoms); Table S2. Spectral data from UV electronic spectrum of K_6_[(VO)SiMo_2_W_9_O_39_]·11H_2_O. In Supplementary Material 2, FTIR vibrational spectra of 27 nanoPOMs (Figures S6–S32); UV electronic spectra of 27 nanoPOMs (Figures S33–S59); Bacterial inoculi microplates prepared from various reference strains inhibited by the 21 analyzed nanoPOMs (Figures S60–S64). In Supplementary Material 3, all syntheses of nanoPOMs (Pages 1–12).
