# Supplementary material for: In Vitro Antibacterial Susceptibility of Different Pathogens to Thirty Nano-Polyoxometalates
Source: Pharmaceuticals (Basel). 2021 Dec 27;15(1):33. doi: 10.3390/ph15010033 (PMC8777622; doi:10.3390/ph15010033)
Supplement: Supplementary file 1 [file pharmaceuticals-15-00033-s001.zip › 2021_SUPPLEMENTARY MATERIAL_1.pdf]

SUPPLEMENTARY MATERIAL

In vitro Antibacterial Susceptibility of Different Pathogens to Thirty Nano-Polyoxometalates

Ștefana Bâlici[a], Dan Rusu[b], Emőke Páll[c], Miuța Filip[d], Flore Chirilă[e], Gheorghe Zsolt Nicula[a], Mihaela Laura Vică[a,*], Rodica Ungur[f], Horea-Vladi Matei[a], Nicodim Iosif Fiț[e]

[a] "Iuliu Hațieganu" University of Medicine and Pharmacy, Faculty of Medicine, Department of Cell and Molecular Biology, Cluj-Napoca, România
[b] "Iuliu Hațieganu" University of Medicine and Pharmacy, Faculty of Pharmacy, Department of Physical-Chemistry, Cluj-Napoca, România
[c] University of Agricultural Science and Veterinary Medicine, Faculty of Veterinary Medicine, Department of Reproduction, Obstetrics and Veterinary Gynecology, Cluj-Napoca, România
[d] "Babeș-Bolyai" University, "Raluca Ripan" Institute for Research in Chemistry, Analytical and Environmental Chemistry Laboratory, Cluj-Napoca, România
[e] University of Agricultural Science and Veterinary Medicine, Faculty of Veterinary Medicine, Department of Microbiology and Immunology, Cluj-Napoca, România
[f] "Iuliu Hațieganu" University of Medicine and Pharmacy, Faculty of Medicine, Department of Medical Rehabilitation, Cluj-Napoca, România
* Correspondence: mvica@umfcluj.ro

Supplemental Table of Contents

The synthesis method and physico-chemical characterization of $K_6[(VO)SiMo_2W_9O_{39}]\cdot 11H_2O$ (POM 7) and its ligand $K_8[SiMo_2W_9O_{39}]\cdot 14H_2O$ (L 7) **2**
Figure S1 TG/DTG/DTA curves of $K_6[(VO)SiMo_2W_9O_{39}]\cdot 11H_2O$ **4**
Figure S2 The proposed structure for POM 7: $K_6[(VO)SiMo_2W_9O_{39}]\cdot 11H_2O$ **4**
Figure S3 FTIR spectra of POM 7, $K_6[(VO)SiMo_2W_9O_{39}]\cdot 11H_2O$, and his ligand (L7), $K_8[SiMo_2W_9O_{39}]\cdot 14H_2O$ **5**
Figure S4 UV (upper right window) and VIS electronic spectra of $K_6[(VO)SiMo_2W_9O_{39}]\cdot 11H_2O$ **6**
Figure S5 ESR spectrum of $K_6[(VO)SiMo_2W_9O_{39}]\cdot 11H_2O$ complex powder at room temperature (solid line) and its simulated spectrum (dashed line) **8**
Table S1. FTIR spectral data (M = metal atoms) **5**
Table S2. Spectral data from UV electronic spectrum of $K_6[(VO)SiMo_2W_9O_{39}]\cdot 11H_2O$ **7**

1. Synthesis and physico-chemical characterization of $K_6[(VO)SiMo_2W_9O_{39}]\cdot 11H_2O$

The mono-lacunary Keggin with mixed addenda atoms, $K_6[(VO)SiMo_2W_9O_{39}]\cdot 11H_2O$ (POM7), with a new formula proposed here was first prepared (using the two step method) and characterized by us as described below. Both the POM 7 and its ligand $K_8[SiMo_2W_9O_{39}]\cdot 14H_2O$ (L7) were identified by ultraviolet (UV), Fourier Transform Infrared (FTIR) and Electron Spin Resonance (ESR) spectroscopy. The analytical characterization of POM 7 by elemental analysis and thermal analysis data is described in Table 2 of this paper.

1.1. The synthesis of ligand L 7, $K_8[SiMo_2W_9O_{39}]\cdot 14H_2O$

The ligand $K_8[SiMo_2W_9O_{39}]\cdot 14H_2O$ (L7) was prepared as follows: 20 mmol (4.84 g) $Na_2MoO_4\cdot 2H_2O$ was dissolved in 40 mL bidistilled and deionized hot water, then 90 mmol (29.69 g) $Na_2WO_4\cdot 2H_2O$ and very quickly 10 mmol (1.22 g) Na_2SiO_3 were successively added under continuous stirring. This solution was acidified with 6M HCl to a pH of 4.5 and refluxed for three hours. Finally, fine powdered KCl was gradually added to this hot solution, under continuous stirring for 10 minutes. The precipitate was filtered under vacuum using a medium porosity frit, and the resulting white powder was dried in a stream of air. The compound was further dried in a desiccator, 8.2 mmol (25.12 g) of $K_8[SiMo_2W_9O_{39}]\cdot 14H_2O$ being obtained at a yield of 82%. The equation of the chemical reaction to obtain the ligand for the mono-lacunary Keggin with mixed addenda atoms (Mo and W) was:

$$9WO_4^{2-}+2MoO_4^{2-}+SiO_3^{2-}+28H^+\rightarrow\alpha\text{-}[SiMo_2W_9O_{39}]^{8-}+14H_2O.$$

1.2. The synthesis of POM 7, $K_6[(VO)SiMo_2W_9O_{39}]\cdot 11H_2O$

2 mmol (6.13 g) of $K_8[SiMo_2W_9O_{39}]\cdot 14H_2O$ was dissolved in a minimum volume (20 mL) of bidistilled and deionized hot water (heated to 70ºC) under continuous stirring, and 2M H_2SO_4 was added until a pH of 4. A solution obtained by dissolving 2 mmol (0.51 g) $VOSO_4\cdot 5H_2O$ in 20 mL of distilled water was then added drop by drop, and the mixture was stirred for 30 minutes. 10.06 mmol (0.75 g) of fine powdered KCl was gradually added under stirring.

This solution with the formed compound was cooled to room temperature, and stored to 4ºC, brought to room temperature 24 hours later and dark blue POM7 crystals recovered after

three days. This POM was recrystallized from a minimum amount of warm bidistilled and deionized water, and then was dried in desiccator. Finally 1.48 mmol (4.52 g) were obtained with a final yield of 74%.

The reaction of vanadyl ions $(VO)^{2+}$ with $[SiMo_2W_9]^{8-}$ in aqueous medium, followed by the isolation of these complexes as potassium salts and their recrystallization from warm bidistilled and deionized water was carried out with good yields, according to the reaction:

$$\alpha\text{-}K_8[SiMo_2W_9O_{39}] + VOSO_4 \rightarrow \alpha\text{-}K_6[(VO)SiMo_2W_9O_{39}] + K_2SO_4\text{, at a pH=4.}$$

2. Physico-chemical characterization of $K_6[(VO)SiMo_2W_9O_{39}]\cdot 11H_2O$ (POM 7)

The thermal stability and behavior of POM 7 was investigated by thermogravimetric analysis of the vanadyl-tungsten-silicate complexes, performed after heating the samples at 180ºC for 30 minutes. The presence of 11 crystallization/lattice water molecules was confirmed by a weight loss of 6.62% between 25-170ºC, as seen on the thermogravimetry (TG) curve in Figure S1. The water loss occurred in two stages, as evidenced on the derivative thermogravimetry (DTG) curve in Figure S1. The dehydration process was accompanied by an endothermic event between ~25-170ºC, which is observed on the differential thermal analysis (DTA) curve in the same figure.

According to literature data, the first exothermic peak of the DTA curve usually occurs at 20-30ºC over the POMs' decomposition temperature [12,13] and is considered a sign of their thermal stability [22,30]. The first exothermic peak of POM7 was observed at 489ºC, indicating a good thermal stability of the complex. The second exothermic peak, at 560ºC, can be attributed to some phase transitions of the oxides resulting from the polyoxoanion decomposition.

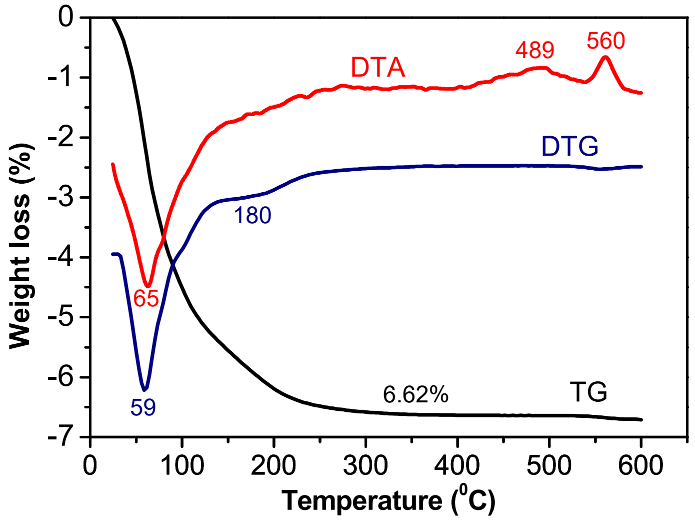

Figure S1. TG/DTG/DTA curves of $K_6[(VO)SiMo_2W_9O_{39}]\cdot11H_2O$.

The proposed structure of POM 7, a mono-lacunary Keggin type, is illustrated in Figure S2.

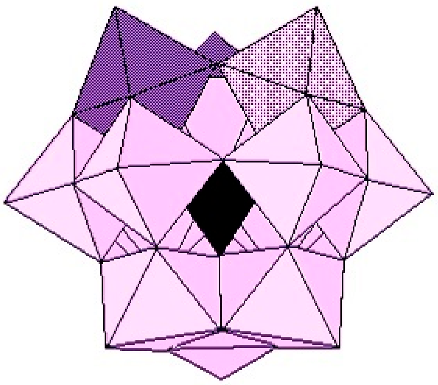

Figure S2. The proposed structure for POM 7: $K_6[(VO)SiMo_2W_9O_{39}]\cdot11H_2O$ (polyhedral representation: MoO_6 octahedra – in dark purple, VO_6 octahedron – in purple dotted, WO_6 octahedra – in light purple, and SiO_4 tetrahedron of the primary heteroatom – in black).

The study of the $(VO)^{2+}$ ion coordination mode was performed by comparing the **FTIR vibrational spectra** of POM 7 ($K_6[(VO)SiMo_2W_9O_{39}]\cdot11H_2O$) with his ligand $K_7[SiMo_2W_9O_{39}]\cdot14H_2O$ (L7). The higher frequency value for the $M{=}O_t$ double bond of POM 7

was compared to same bond in his ligand, demonstrating the involvement of terminal oxygen atoms (O_t) in the vanadyl ion coordination.

The M-O_c-M tricentric bonds corresponding to MO_6 octahedra, joined by common corners, are characterized by a single band. The M-O_c-M bond frequency in POM 7 is 19 cm^{-1} higher than that of his ligand, difference due to the $(VO)^{2+}$ ion coordination, as seen in Table S1 and Figure S3.

Table S1. FTIR spectral data (M = metal atoms).

Attributed bond	$K_8[SiMo_2W_9O_{39}]\cdot 14H_2O$; L 7 ($cm^{-1}$)	$K_6[(VO)SiMo_2W_9O_{39}]\cdot 11H_2O$; POM 7 ($cm^{-1}$)
**ν_{as}(Si-O_i)**	1103 w	1109 w
**ν_{as}(M=O_t)**	947 s	968 s
**ν_{as}(M-O_c-M)**	887 vs, b	906 vs
**ν_{as}(M-O_e-M)**	796 vs	783 vs
	729 s	669 m

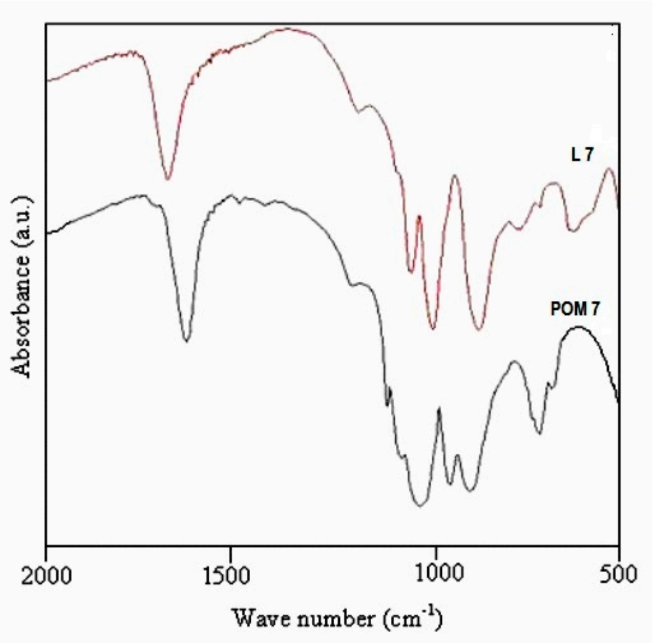

Figure S3. FTIR spectra of POM 7, $K_6[(VO)SiMo_2W_9O_{39}]\cdot 11H_2O$, and his ligand (L7), $K_8[SiMo_2W_9O_{39}]\cdot 14H_2O$; spectral data for both L7 and POM7 were presented in Table 1 in the Supplementary Material, and their contribution and bonds assigned in Table 2 (the last column) of this paper.

The increased frequency of the POM 7 M-O_c-M bonds expresses a shortening of the post-complexation binding compared to the L7 ligand. The M-O_e-M bond has a lower frequency in

POM 7 than in his ligand, which reflects an elongation of this bond in the nanocomplex compared to the vanadium-free ligand. The Si-O_i bond also has a slightly modified frequency, insignificant in POM 7 relative to his ligand. The displacements of all bands in the nanocomplex compared to the ligand suggest the involvement of the oxygen atoms (O_t, O_c and O_e) in the $(VO)^{2+}$ ion coordination.

The UV electronic spectra of the ligand and of the complex (here in Figure S4 and Table S2) presented two characteristic bands of the polyoxoanionic structures at ν_2 = ~200 nm attributed to the bonds M=O_t (ν_2) (O_t, the terminal oxygen atoms), and ν_1 = ~250 nm attributed to the bridge-bonds M-O_b-M (ν_1), where the oxygen atoms (O_b) into the bridge-bonds, with b = c are bridge-bonds via corners, and b = e bridge-bond via edges.

In the POM 7 spectrum, both the ν_2 (50251 cm^{-1}/199 nm) band corresponding to the M=O_t bond and the ν_1 (38759 cm^{-1}/258 nm) band corresponding to the M-O_b-M bond were shifted to higher frequencies than those presented by the ligand, which due to the vanadyl cations coordination by the O_t, O_c and O_e oxygen atoms. All types of oxygen atoms in the POM 7 structure participated in the coordination, and these data were consistent with those obtained from FTIR vibrational spectra.

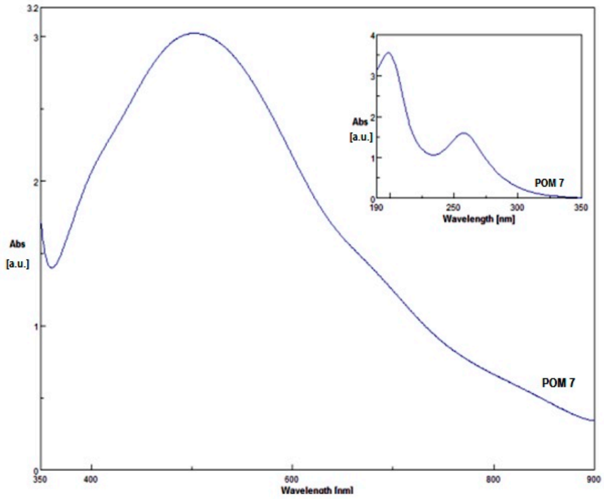

Figure S4. UV (upper right window) and VIS electronic spectra of $K_6[(VO)SiMo_2W_9O_{39}]\cdot 11H_2O$ (recorded in aqueous solution at a concentration of $5x10^{-5}$ mol/L for UV, and $5x10^{-3}$ mol/L for VIS, respectively).

Table S2. Spectral data from UV electronic spectrum of $K_6[(VO)SiMo_2W_9O_{39}]\cdot 11H_2O$.

Attributed bond	$K_6[(VO)SiMo_2W_9O_{39}]\cdot 11H_2O$; POM 7 ($cm^{-1}$/nm)
$\nu_2(M=O_t)$	50251 cm^{-1}/199 nm
$\nu_1(M\text{-}O_{c,e}\text{-}M)$	38759 cm^{-1}/258 nm

In the Visible range, the electronic spectrum of the nanocomplex presented a stronger absorption, over 16000 cm^{-1}, and three transfer bands (as a shoulders) at lower wave number, but higher than the one given by the $[VO(H_2O)_5]^{2+}$ aquaion.

The strong absorbances correspond to the $V^{IV}\rightarrow W^{VI}$ load transfer transitions. The spectrum analysis leads to obtaining of the d-d transitions position for V^{IV} ions. The bands of the complex are assigned to the transitions: $^2A_1(d_{z^2}) \leftarrow {}^2B_2(d_{xy})$, $^2T_{2g} \leftarrow {}^2E_g$, $^2A_{1g} \leftarrow {}^2B_{2g}$ and $^2B_{1g}\longleftarrow {}^2B_{2g}$, according to the molecular orbital theory for vanadyl ions, with a local O_h symmetry, a theory developed in 1962 by Ballhausen and Gray.

The $K_6[(VO)SiMo_2W_9O_{39}]\cdot 11H_2O$ electronic spectrum in Visible range exhibited four bands, and it can be concluded that this compound has a distorted O_h symmetry. The very weak shoulder-like band at 13440 cm^{-1}/744 nm was shifted to lower energy compared to the $[VO(H_2O)_5]^{2+}$ aquaion, and it was assigned to the transition $^2B_{1g} \leftarrow {}^2B_{2g}$.

The 15527 cm^{-1}/644 nm shoulder-like band shifted to higher energy compared to the $[VO(H_2O)_5]^{2+}$ aquaion, and was assigned to the $^2A_{1g}\longleftarrow {}^2B_{2g}$ transition.

The third band at 19880 cm^{-1}/503 nm was very strong in intensity, being shifted to higher energy compared to the aquaion $[VO(H_2O)_5]^{2+}$, and can be attributed to the $^2T_{2g}\longleftarrow {}^2E_g$ transition. Finally, the fourth band at 24752 cm^{-1}/404 nm shifted to lower energy compared to the $[VO(H_2O)_5]^{2+}$ aquaion and can be attributed to $^2A_1(d_{z^2})\longleftarrow {}^2B_2(d_{xy})$ transition.

Electron Spin Resonance (ESR) Spectra

The ESR spectrum of $K_6[(VO)SiMo_2W_9O_{39}]\cdot 11H_2O$ (Figure S5), in powder form, recorded at room temperature, highlights the presence of reduced vanadium species, as also shown by the electronic spectra. The spectrum recorded for the Keggin complex with mixed addenda atoms of Mo, W is axial and suggests the existence of eight hyperfine components, both in the perpendicular bands $\perp$ and in the parallel ones, due to the hyperfine coupling of the unpaired electron with nuclear spin, I = 7/2, of the ^{51}V isotope with natural abundance of 99.8%.

The absence of signals for Mo^{5+} and W^{5+} ions in the ESR spectrum confirms that all Mo and W ions are in a +6 oxidation state.

ESR spectral parameters for $K_6[(VO)SiMo_2W_9O_{39}]\cdot 11H_2O$ have the following values:

$g_{II}=1.922$, $g_{\perp}=1.972$, $A_{II}=181G$, $A_{\perp}= 63G$

$\Delta B_{II(p-p)} = 30G$ $\Delta B_{\perp(p-p)} = 33G.$

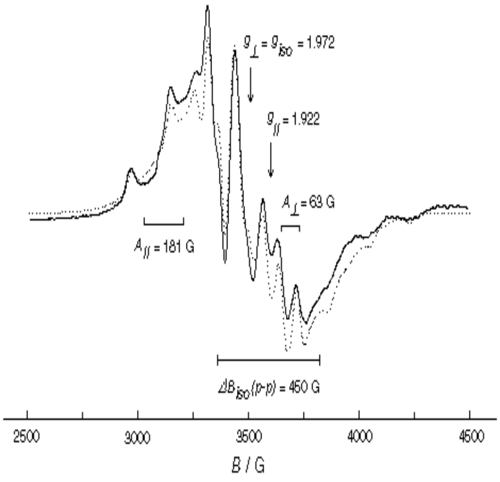

Figure S5. ESR spectrum of $K_6[(VO)SiMo_2W_9O_{39}]\cdot 11H_2O$ complex powder at room temperature (solid line) and its simulated spectrum (dashed line).

The two components (parallel and perpendicular) contribute equally (50%) to the formation of the spectra, suggesting that species reduced by one electron and the species monoprotonated by 2 electrons are present in equal amounts in the sample. The main tensors of the axes g and A were supposed to coincide with g_{II} and A_{II} of the $V=O_t$ bond. Differences between experimental and spectrally simulated data indicate a small rhombic distortion of the vanadium octahedron (VO_6).
