# Supplementary material for: In Vitro Antibacterial Susceptibility of Different Pathogens to Thirty Nano-Polyoxometalates
Source: Pharmaceuticals (Basel). 2021 Dec 27;15(1):33. doi: 10.3390/ph15010033 (PMC8777622; doi:10.3390/ph15010033)

<u>SUPPLEMENTARY MATERIAL 2</u>

In vitro Antibacterial Susceptibility of Different Pathogens to Thirty Nano-Polyoxometalates

Ştefana Bâlici[a], Dan Rusu[b], Emőke Páll[c], Miuţa Filip[d], Flore Chirilă[e], Gheorghe Zsolt Nicula[a], Mihaela Laura Vică[a,*], Rodica Ungur[f], Horea-Vladi Matei[a], Nicodim Iosif Fiţ[e]

[a] "Iuliu Haţieganu" University of Medicine and Pharmacy, Faculty of Medicine, Department of Cell and Molecular Biology, Cluj-Napoca, România
[b] "Iuliu Haţieganu" University of Medicine and Pharmacy, Faculty of Pharmacy, Department of Physical-Chemistry, Cluj-Napoca, România
[c] University of Agricultural Science and Veterinary Medicine, Faculty of Veterinary Medicine, Department of Reproduction, Obstetrics and Veterinary Gynecology, Cluj-Napoca, România
[d] "Babeş-Bolyai" University, "Raluca Ripan" Institute for Research in Chemistry, Analytical and Environmental Chemistry Laboratory, Cluj-Napoca, România
[e] University of Agricultural Science and Veterinary Medicine, Faculty of Veterinary Medicine, Department of Microbiology and Immunology, Cluj-Napoca, România
[f] "Iuliu Haţieganu" University of Medicine and Pharmacy, Faculty of Medicine, Department of Medical Rehabilitation, Cluj-Napoca, România
* Correspondence: mvica@umfcluj.ro

<u>Supplemental Table of Contents</u>

FTIR vibrational spectra of 27 nanoPOMs (Figures S6 – S32) 3
Figures S6 – S9. FTIR spectra of nanoPOMs with saturated Keggin structures 3
Figures S10 – S15. FTIR spectra of nanoPOMs with mono-lacunary Keggin structures 5
Figures S16 – S18. FTIR spectra of nanoPOMs with tri-lacunary Keggin structures 8
Figure S19. FTIR spectrum of nanoPOM with tri-lacunary *pseudo*-Keggin structures 9
Figures S20 –S23. FTIR spectra of nanoPOMs with tri-lacunary Keggin/sandwich type structures 10
Figures S24 – S26. FTIR spectra of nanoPOMs with tri-lacunary *pseudo*-Keggin/sandwich type structures 12
Figures S27 – S31. FTIR spectra of nanoPOMs with cluster structures 13
Figure S32. FTIR spectrum of nanoPOM, mono-lacunary Wells-Dawson with mixed addenda atoms 16

UV electronic spectra of 27 nanoPOMs (Figures S33 – S59) **17**
Figure S33 – S36. UV electronic spectra of nanoPOMs with saturated Keggin structures **17**
Figure S37 – S42. UV electronic spectra of nanoPOMs with mono-lacunary Keggin structures **18**
Figure S43 – S45. UV electronic spectra of nanoPOMs with tri-lacunary Keggin structures **19**
Figure S46. UV electronic spectrum of nanoPOM with tri-lacunary *pseudo*-Keggin structures **20**
Figure S47 – S50. UV electronic spectra of nanoPOMs with tri-lacunary Keggin/sandwich type structures **20**
Figure S51 – S53. UV electronic spectra of nanoPOMs with tri-lacunary *pseudo*-Keggin/sandwich type structures **22**
Figure S54 – S58. UV electronic spectra of nanoPOMs with cluster structures **23**
Figure S59. UV electronic spectrum of nanoPOM, mono-lacunary Wells-Dawson with mixed addenda atoms **24**

Bacterial inoculi microplates prepared from various reference strains inhibited by the 21 analyzed nanoPOMs (Figures S60 – S64) **25**
Figure S60. Inhibition aspects for the 21 nanoPOMs analyzed (in microplates of bacterial inoculum prepared from reference strain ATCC 6538P– *Staphylococcus aureus*): A-C. Active nanoPOMs, D. Control samples. **25**
Figure S61. Inhibition aspects for the 21 nanoPOMs analyzed (in microplates of bacterial inoculum prepared from reference strain ATCC 14579 – *Bacillus cereus*): A-C. Active nanoPOMs, D. Control samples. **25**
Figure S62. Inhibition aspects for the 21 nanoPOMs analyzed (in microplates of bacterial inoculum prepared from reference strain ATCC 13076 – *Salmonella enteritidis*): A-C. Active nanoPOMs, D. Control samples. **26**
Figure S63. Inhibition aspects for the 21 nanoPOMs analyzed (in microplates of bacterial inoculum prepared from reference strain ATCC 10536 – *Escherichia coli*): A-C. Active nanoPOMs, D. Control samples. **26**
Figure S64. Inhibition aspects for the 21 nanoPOMs analyzed (in microplates of bacterial inoculum prepared from reference strain ATCC 27853 – *Pseudomonas aeruginosa*): A-C. Active nanoPOMs, D. Control samples. **27**

FTIR vibrational spectra of 27 nanoPOMs

<u>FTIR spectra of nanoPOMs with saturated Keggin structures</u>

Figure S6. FTIR spectrum of POM 12, $H_4[SiW_{12}O_{40}]\cdot 14H_2O$

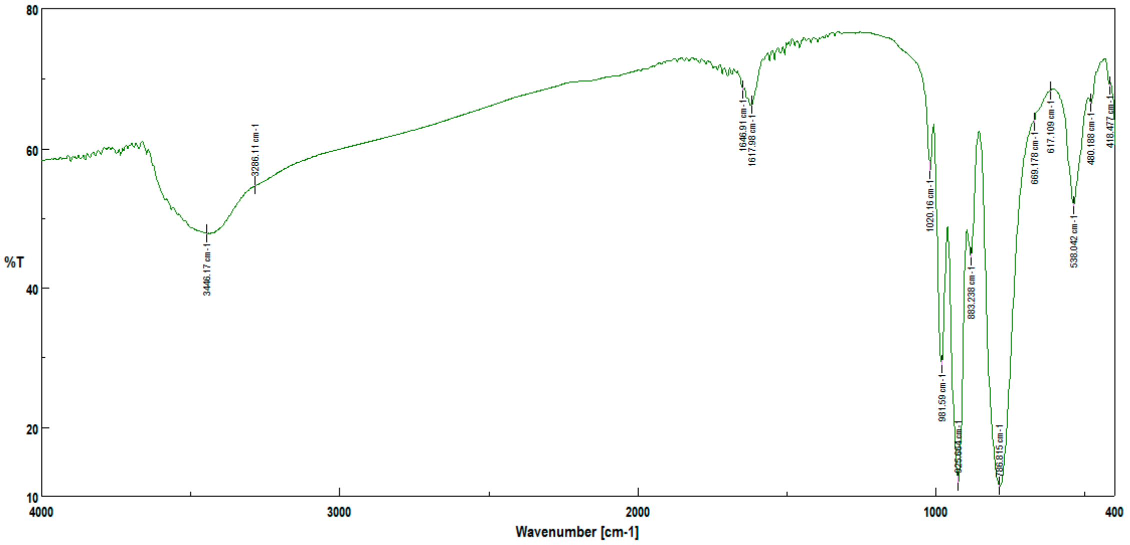

Figure S7. FTIR spectrum of POM 13, $H_3[PW_{12}O_{40}]\cdot 12H_2O$

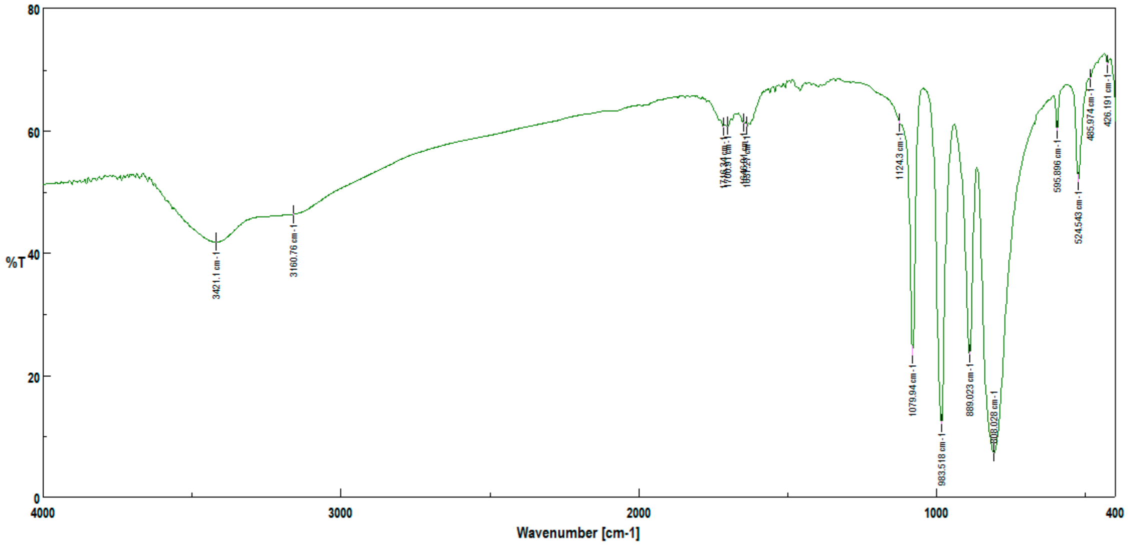

Figure S8. FTIR spectrum of POM 14, $H_3[PMo_{12}O_{40}]\cdot 13H_2O$

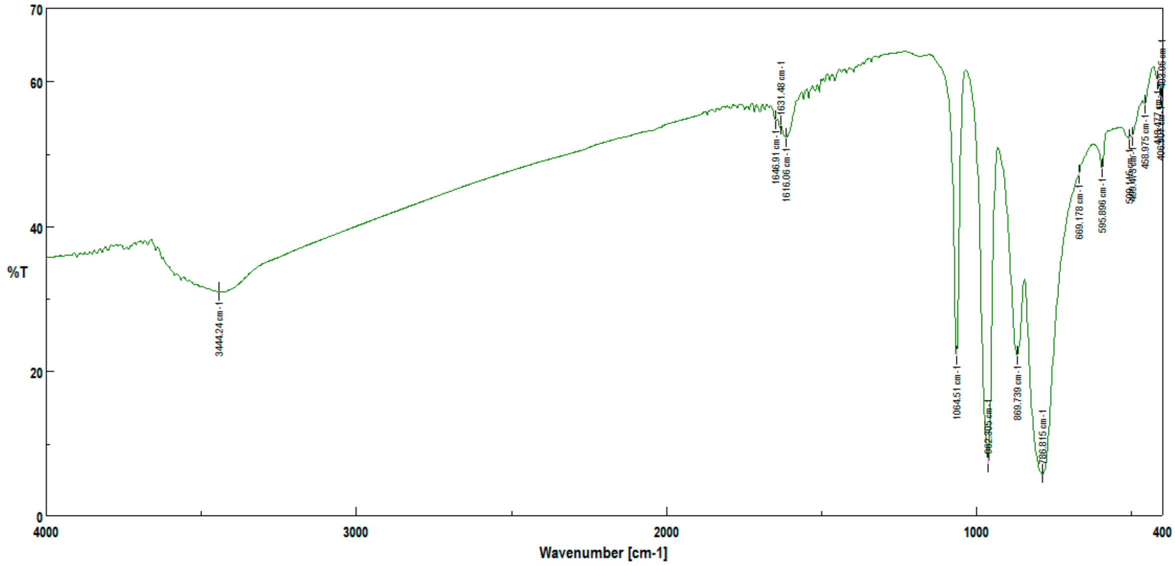

Figure S9. FTIR spectrum of POM 6, $Na_6[PMo_9^{VI}V_3^{V}O_{40}]\cdot 16H_2O$, Keggin with mixed addenda atoms

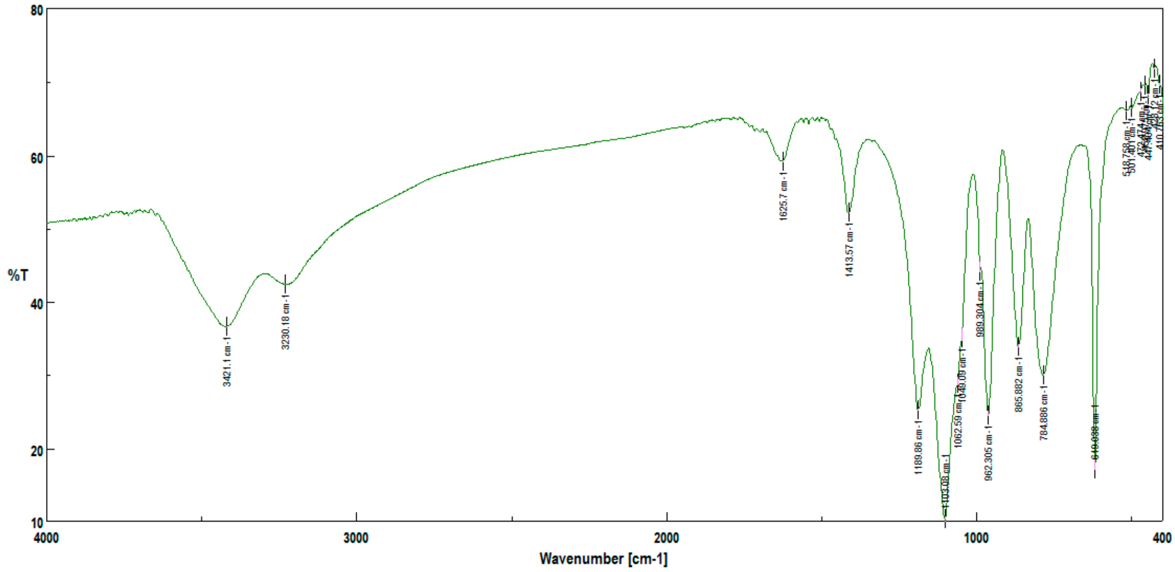

FTIR spectra of nanoPOMs with mono-lacunary Keggin structures

Figure S10. FTIR spectrum of POM 1, $Na_4[Fe^{III}(H_2O)PMo_{11}O_{39}]\cdot 18H_2O$

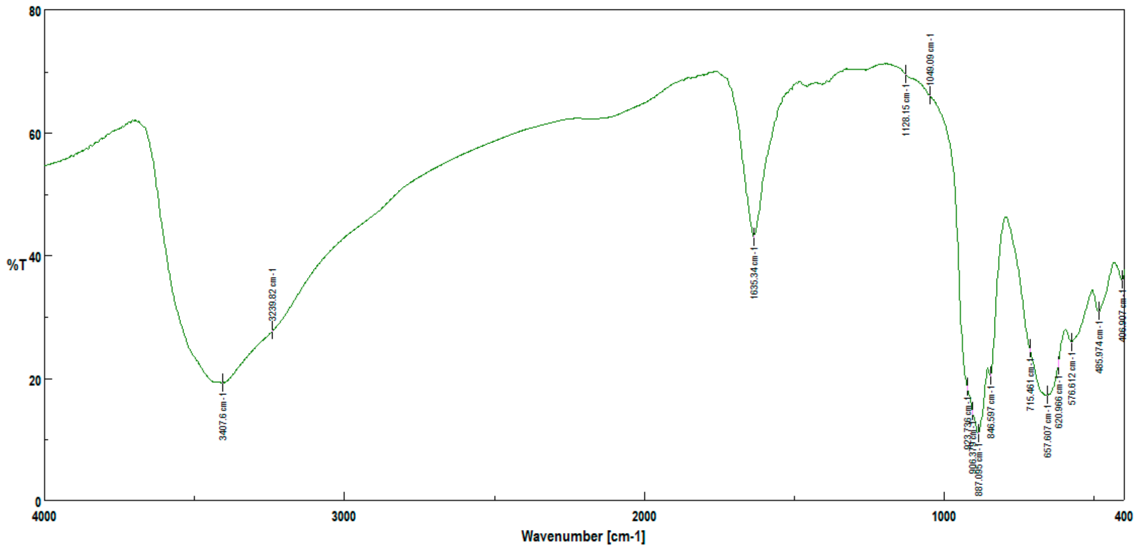

Figure S11. FTIR spectrum of POM 3, $Na_8[SiW_{11}O_{39}]\cdot 12H_2O$

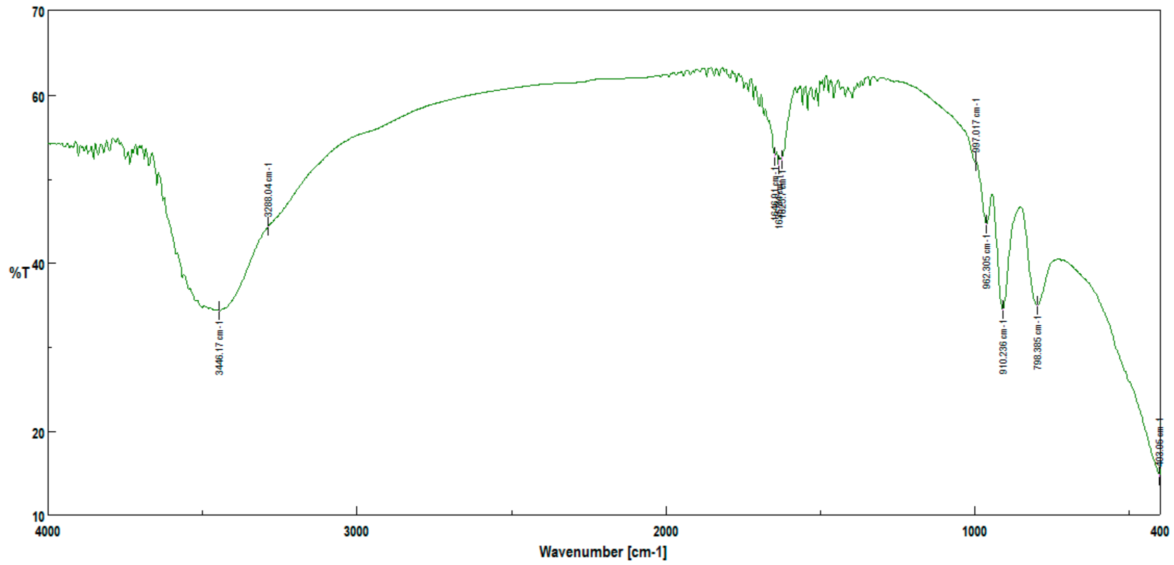

Figure S12. FTIR spectrum of POM 23a,b, $Na_5[Fe^{III}(H_2O)SiW_{11}O_{39}]\cdot 24H_2O$

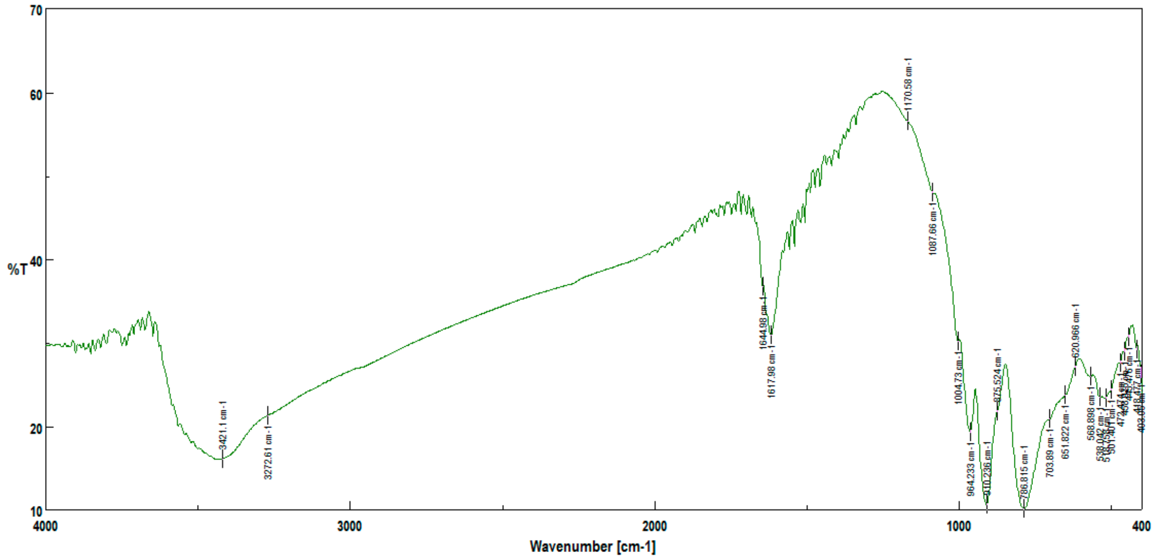

Figure S13. FTIR spectrum of POM 24a,b, $Na_5[Fe^{III}(H_2O)GeW_{11}O_{39}]\cdot 26H_2O$

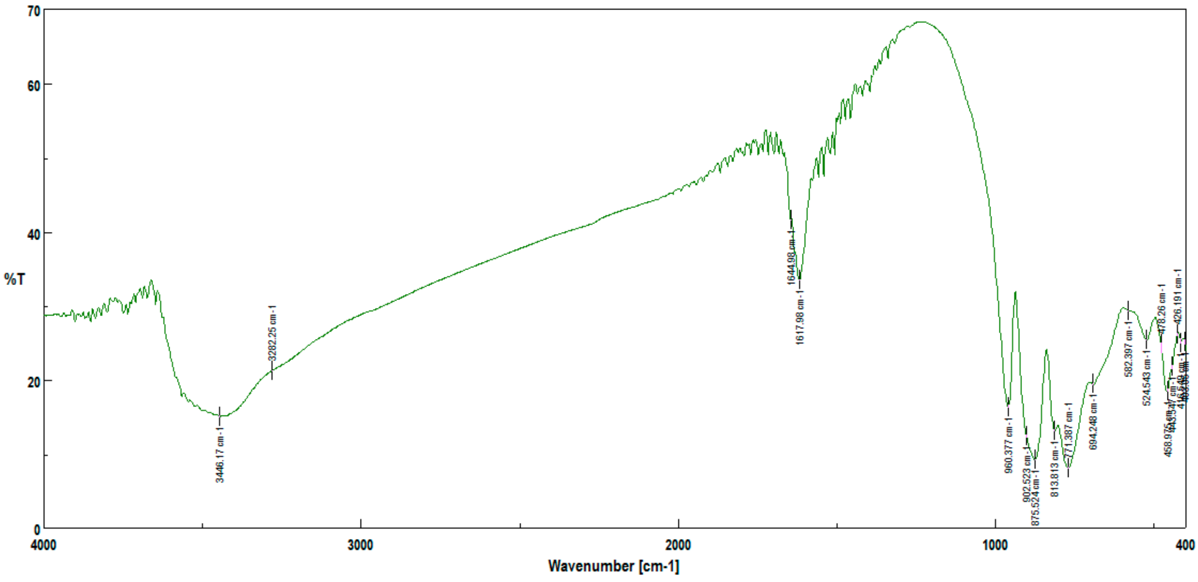

Figure S14. FTIR spectrum of POM 30, $K_6[SiV^{IV}W_{11}O_{40}]\cdot 12H_2O$

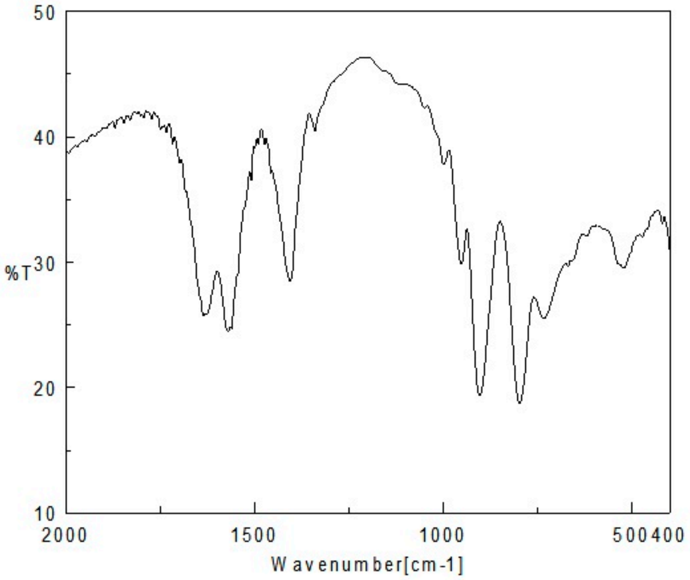

Figure S15. FTIR spectrum of POM 21, $K_6[Co(H_2O)SiMo_2W_9O_{39}]\cdot 14H_2O$, mono-lacunary Keggin with mixed addenda atoms

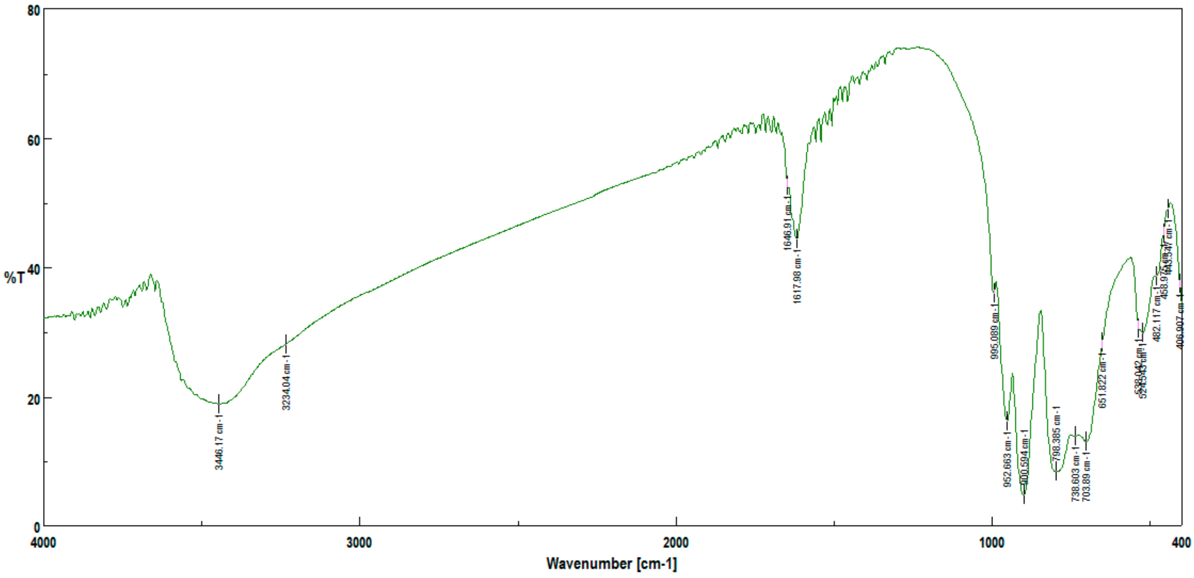

FTIR spectra of nanoPOMs with tri-lacunary Keggin structures

Figure S16. FTIR spectrum of POM 5, $K_3[(VO)_3PMo_9O_{34}]\cdot 14H_2O$

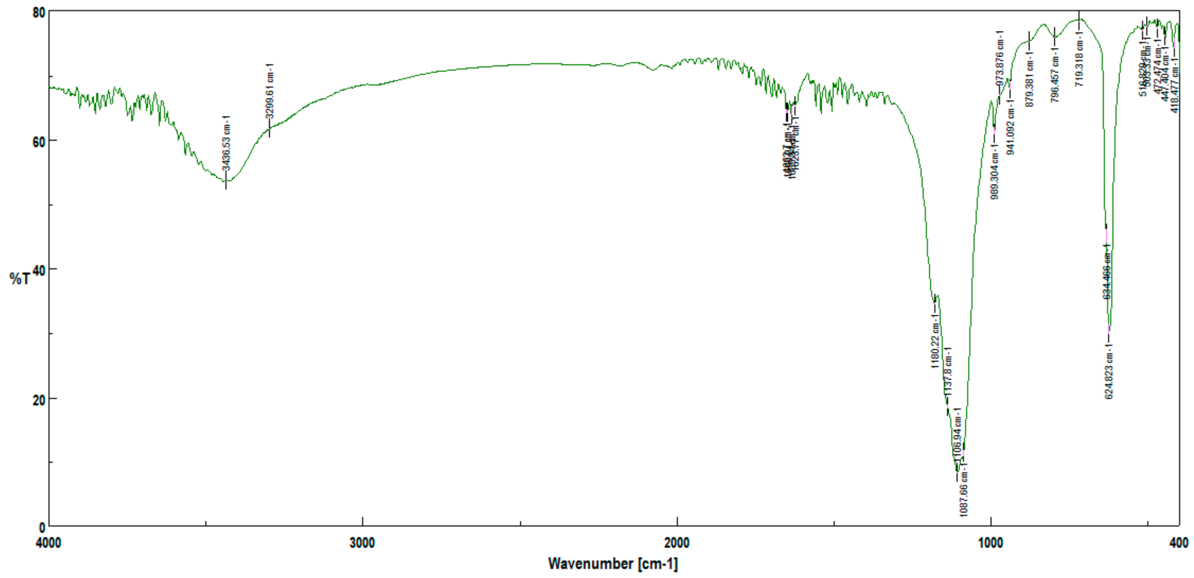

Figure S17. FTIR spectrum of POM 16a,b, $Na_{10}[SiW_9O_{34}]\cdot 24H_2O$

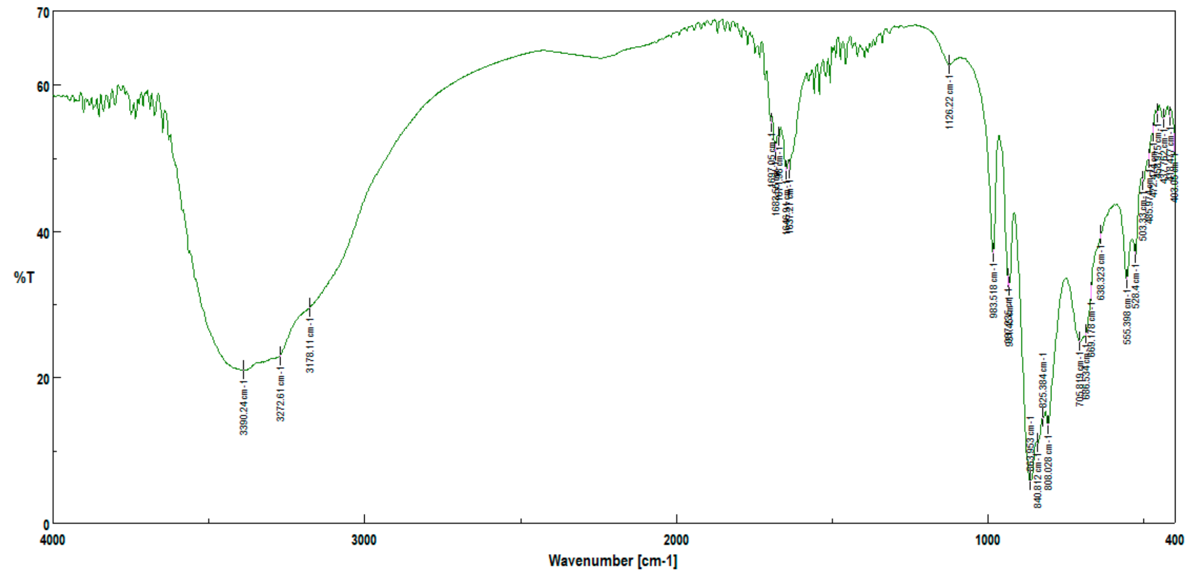

Figure S18. FTIR spectrum of POM 18, $Na_8H[PW_9O_{34}]\cdot 20H_2O$

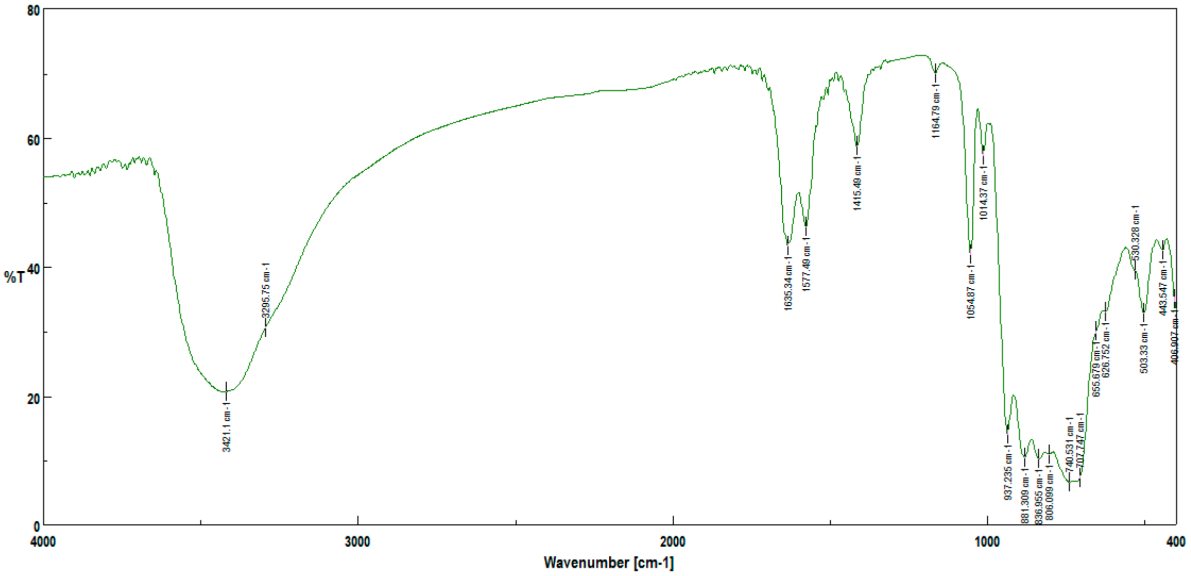

<u>FTIR spectrum of nanoPOM with tri-lacunary *pseudo*-Keggin structures</u>

Figure S19. FTIR spectrum of POM 15, $Na_9[SbW_9O_{33}]\cdot 19{,}5H_2O$

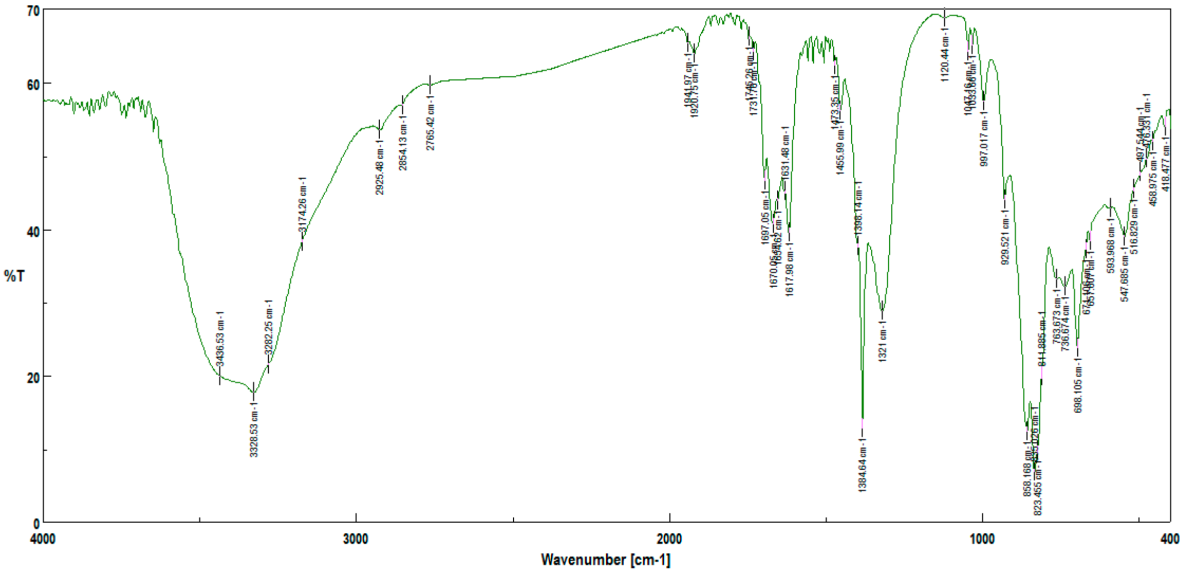

FTIR spectra of nanoPOMs with tri-lacunary Keggin/sandwich type structures

Figure S20. FTIR spectrum of POM 2, $Na_9[Fe_3(H_2O)_3(PMo_9O_{34})_2]$

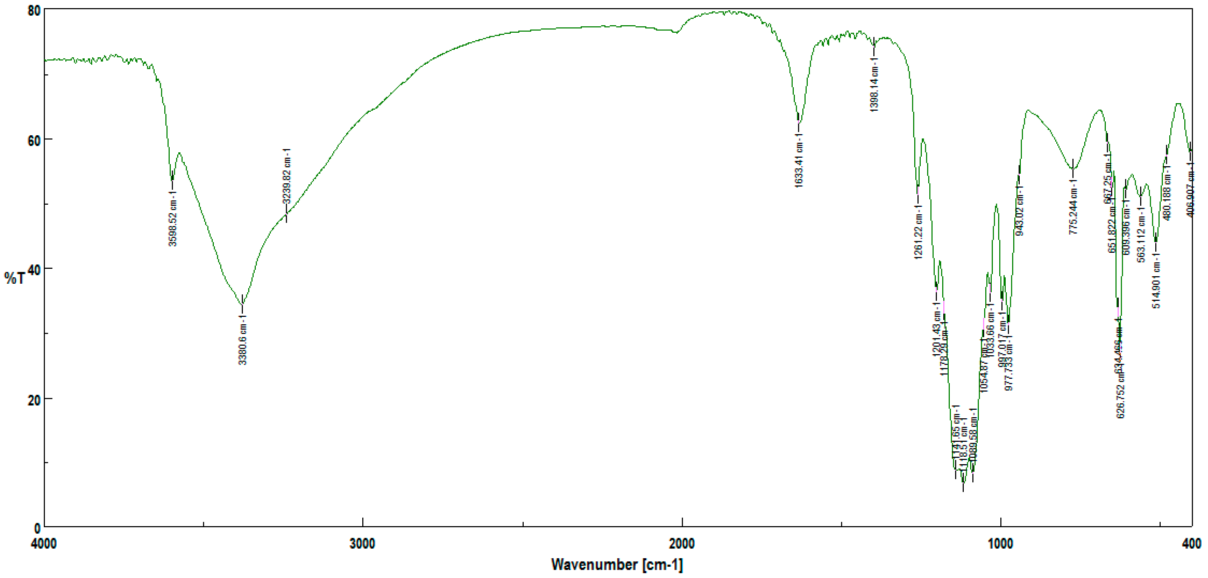

Figure S21. FTIR spectrum of POM 4, $Na_{11}[Fe_3(H_2O)_3(SiW_9O_{34})_2]\cdot 25H_2O$

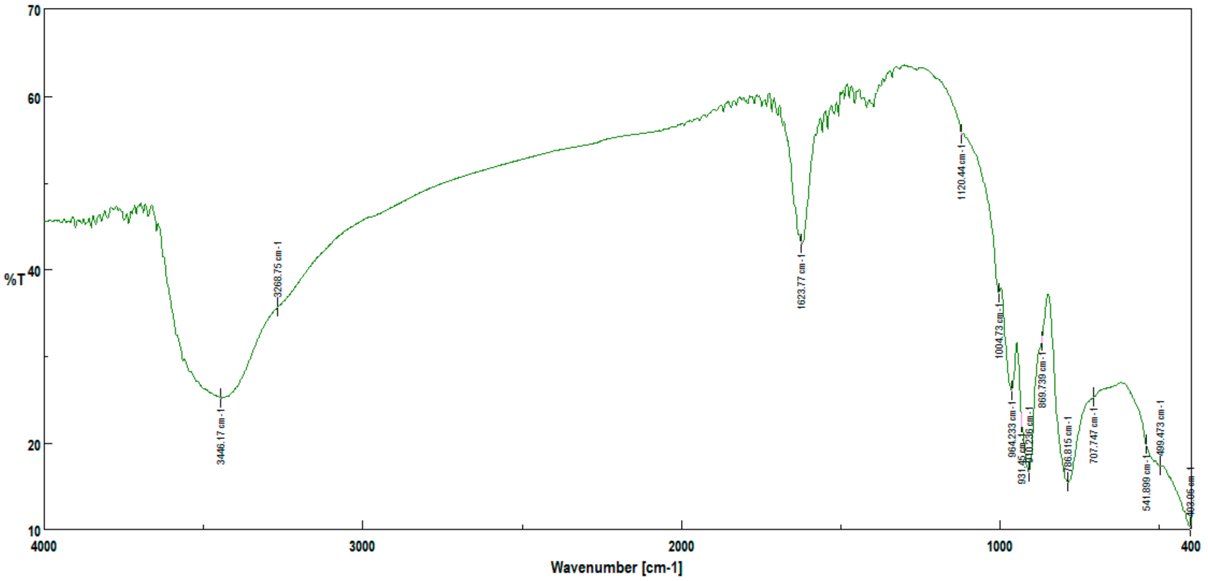

Figure S22. FTIR spectrum of POM 8, $K_{10}[(VO)_4(PW_9O_{34})_2]\cdot 26H_2O$

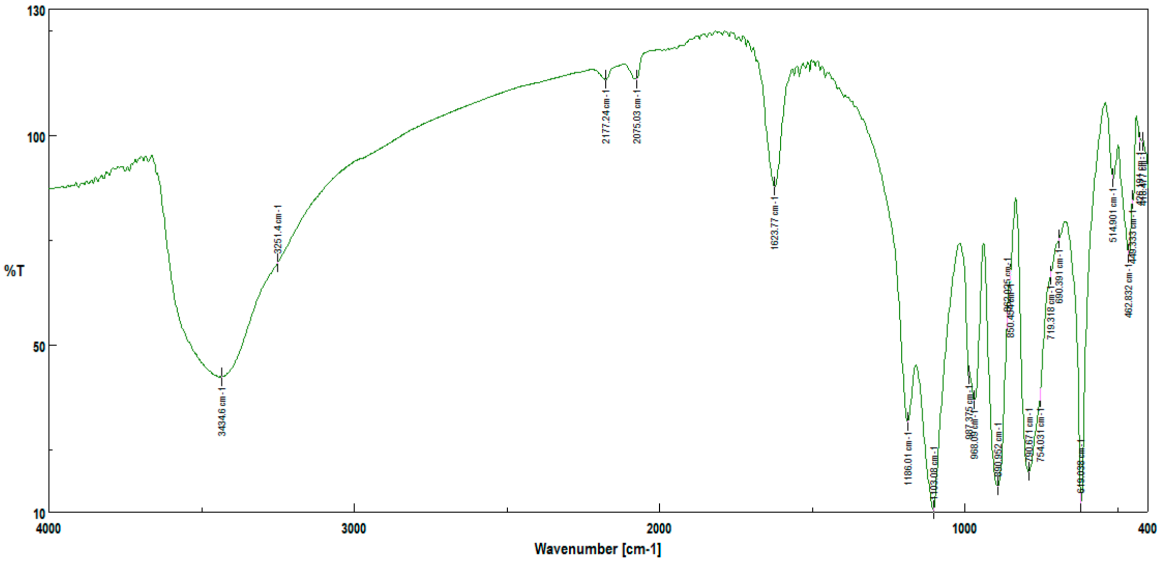

Figure S23. FTIR spectrum of POM 27, $Na_{14}[Mn_3(H_2O)_3(SiW_9O_{34})_2]\cdot 28H_2O$

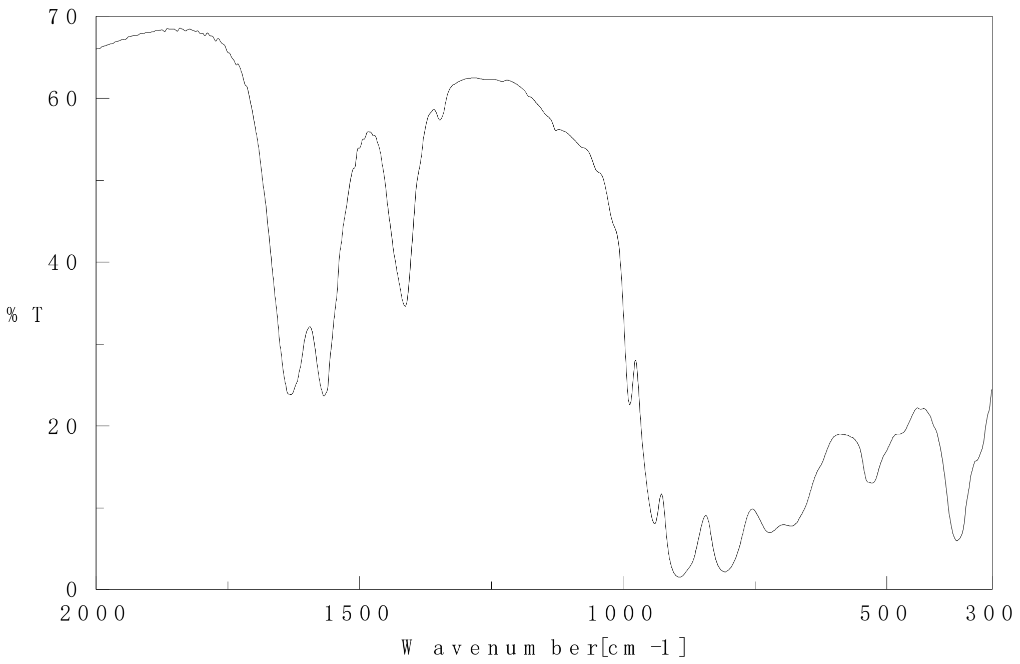

<u>**FTIR spectra of nanoPOMs with tri-lacunary *pseudo*-Keggin/sandwich type structures**</u>

FTIR spectrum of POM 10, $K_{11}H[(VO)_3(Sb^{III}W_9O_{33})_2]\cdot 27H_2O$

Published in our previous paper, cited in this paper as reference 22

Figure S24. FTIR spectrum of POM 25, $Na_{10}[Mn_4(H_2O)_2(AsW_9O_{34})_2]\cdot 27H_2O$

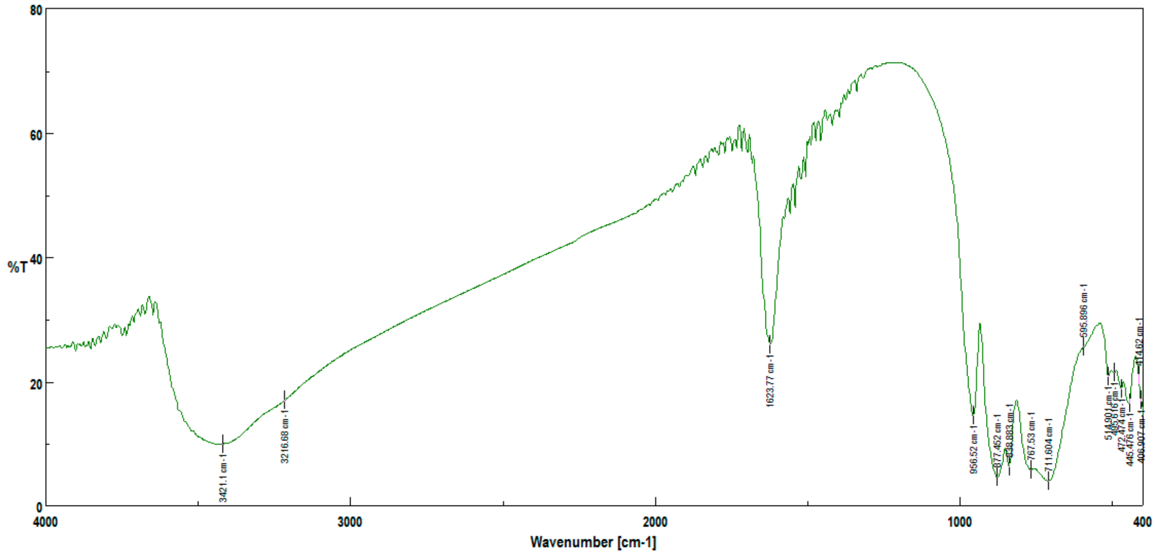

Figure S25. FTIR spectrum of POM 26, $Na_{12}[Co_3(H_2O)_3(BiW_9O_{33})_2]\cdot 37H_2O$

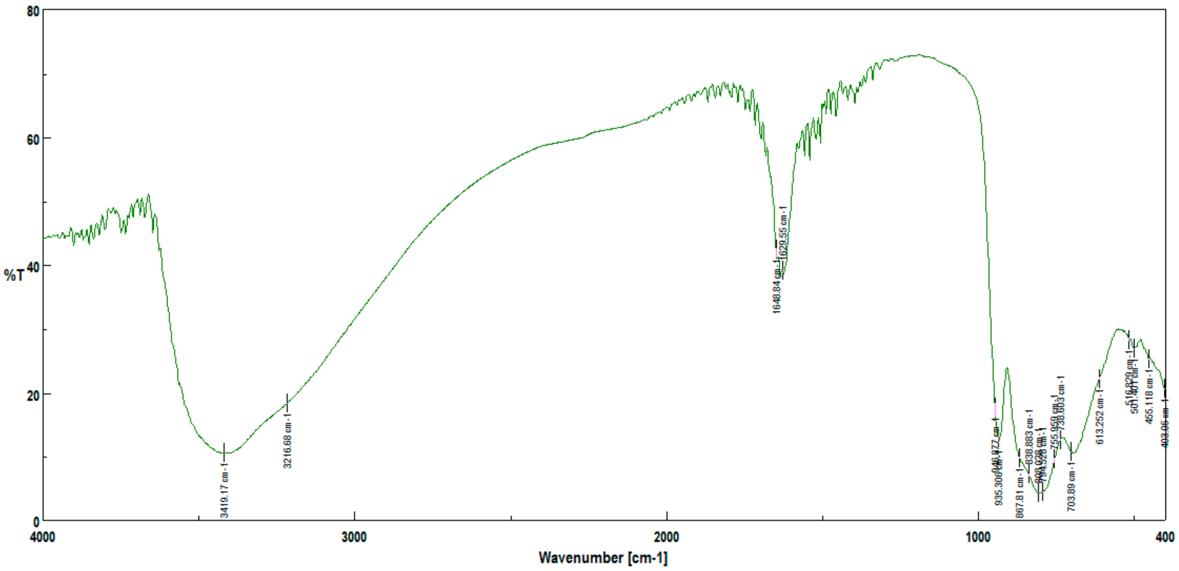

Figure S26. FTIR spectrum of POM 9, $K_{10}[(VO)_4(AsW_9O_{34})_2]\cdot21H_2O$

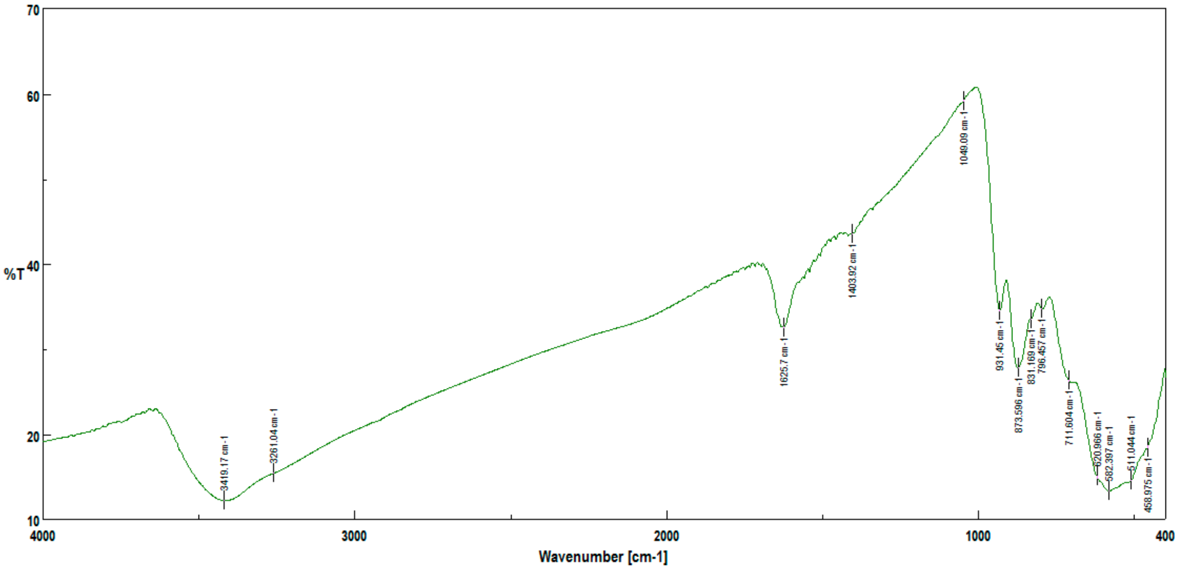

FTIR spectra of nanoPOMs with cluster structures

Figure S27. FTIR spectrum of POM 11, $Na_{12}[Sb_2W_{22}O_{74}(OH)_2]\cdot38H_2O$

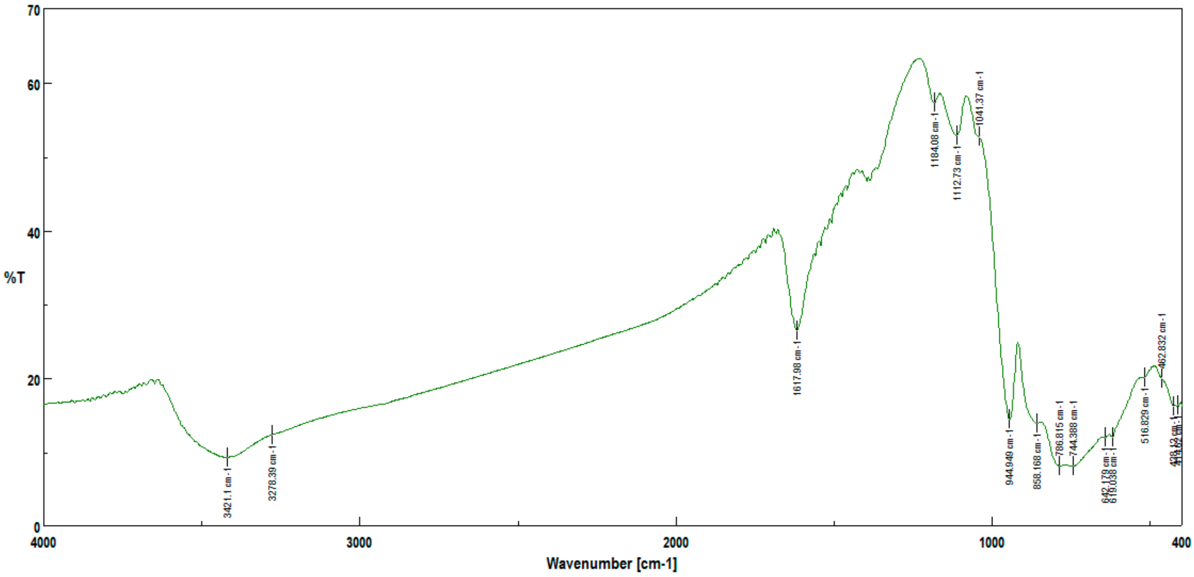

Figure S28. FTIR spectrum of POM 17, $Na_{27}[NaAs_4W_{40}O_{140}]\cdot 42H_2O$

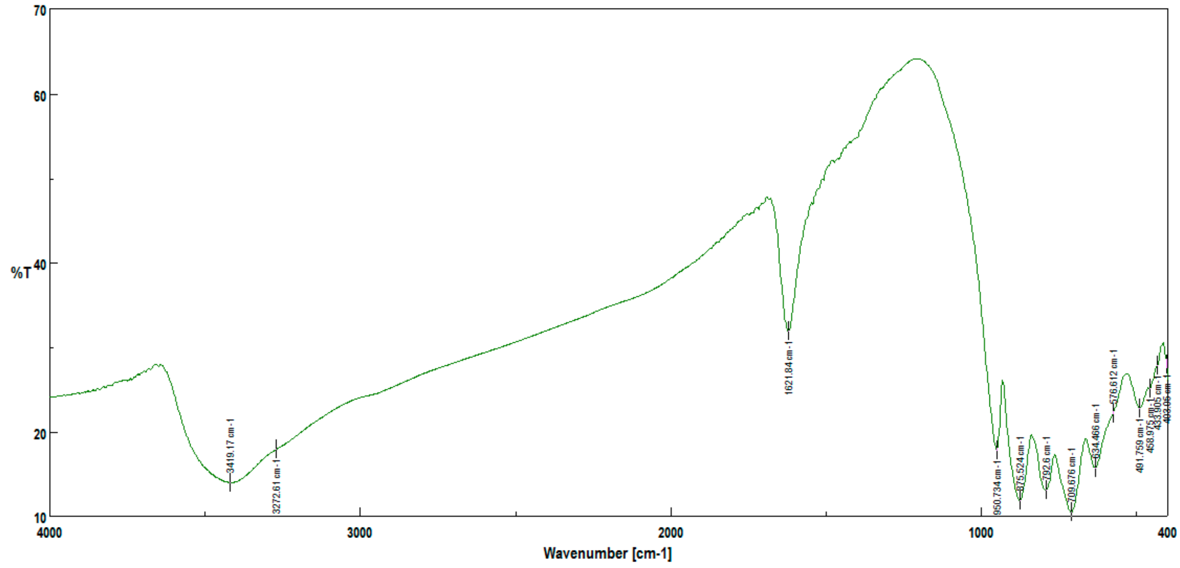

Figure S29. FTIR spectrum of POM 19, $(NBu_4)_{27}[NaAs_4Mo_{40}O_{140}]\cdot 12H_2O$

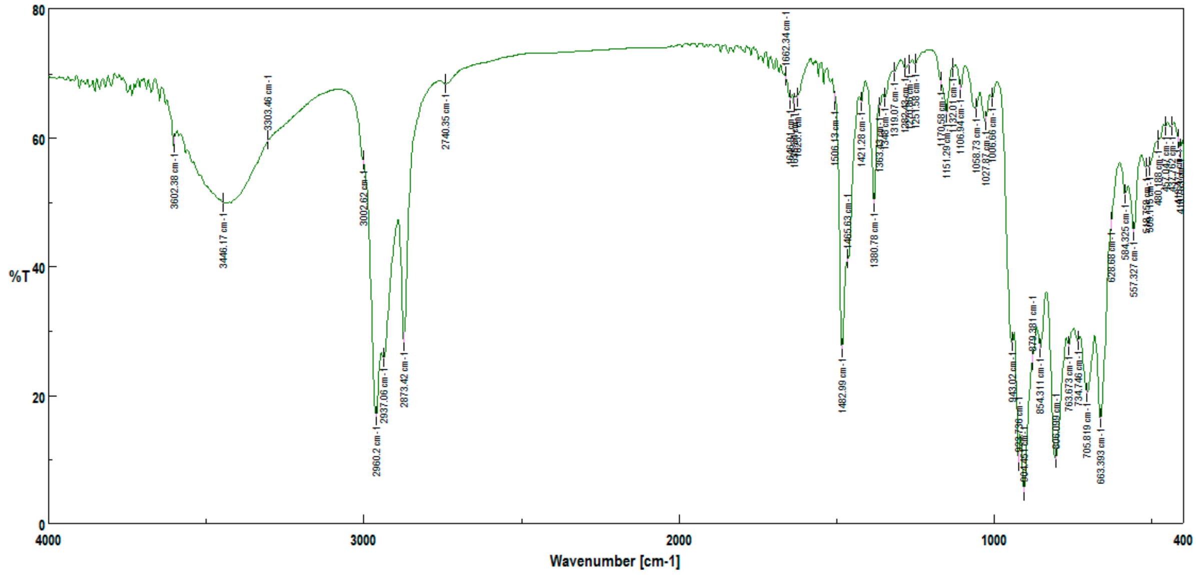

Figure S30. FTIR spectrum of POM 20, $(Bu_3Sn)_{18}[NaSb_9W_{21}O_{86}]$

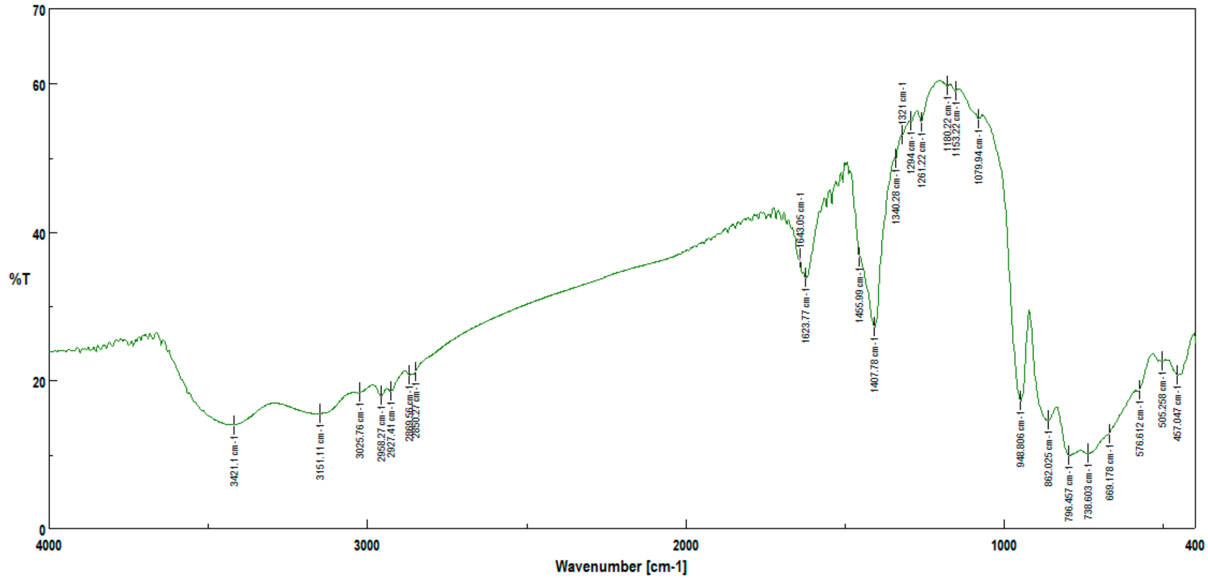

FTIR spectrum of POM 28, $(NH_4)_4(NBu_4)_5[Na(BuSn)_3Sb_9W_{21}O_{86}]\cdot 17H_2O$
Published in our previous paper, cited in this paper as reference 22

Figure S31. FTIR spectrum of POM 29, $K_{27}[NaAs_4W_{40}O_{140}]\cdot 52H_2O$

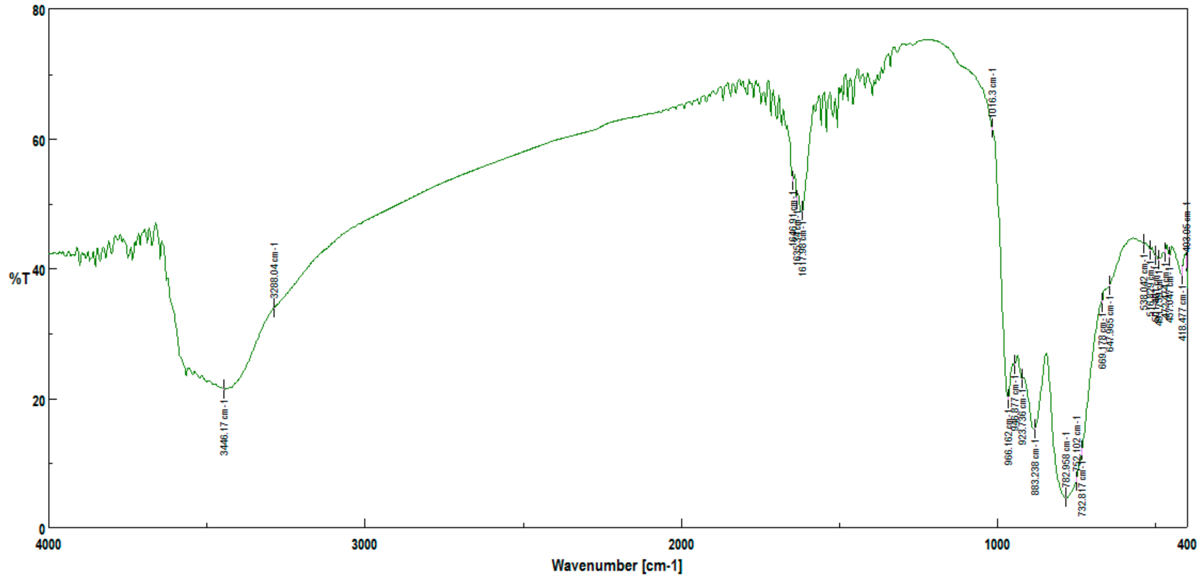

FTIR spectrum of nanoPOM, mono-lacunary Wells-Dawson with mixed addenda atoms

Figure S32. FTIR spectrum of POM 22, $K_{10}[Co(H_2O)Si_2MoW_{16}O_{61}]\cdot18H_2O$

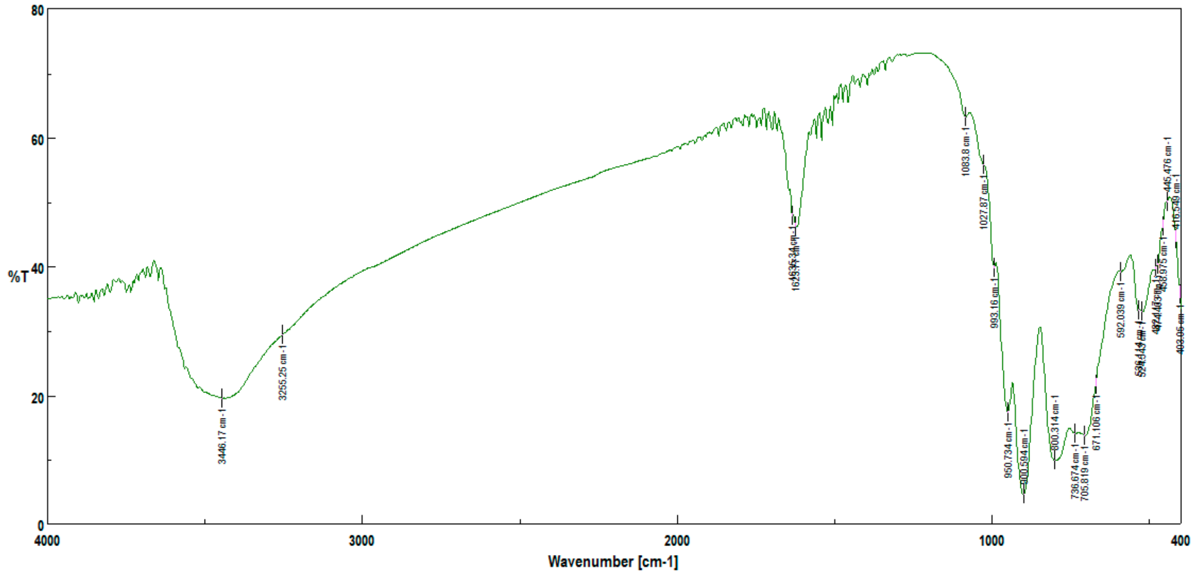

UV electronic spectra of 27 nanoPOMs

UV electronic spectra of nanoPOMs with saturated Keggin structures

Figure S33. UV electronic spectrum of POM 12, $H_4[SiW_{12}O_{40}]\cdot 14H_2O$

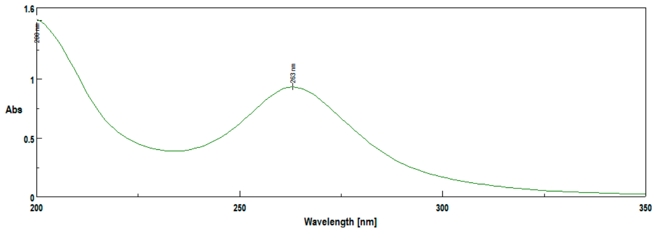

Figure S34. UV electronic spectrum of POM 13, $H_3[PW_{12}O_{40}]\cdot 12H_2O$

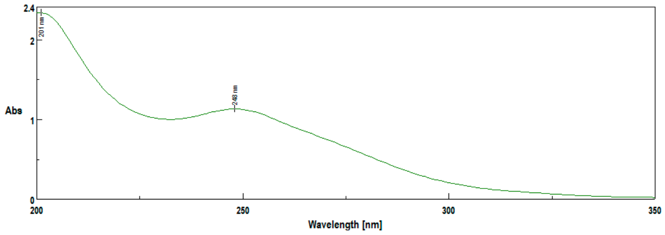

Figure S35. UV electronic spectrum of POM 14, $H_3[PMo_{12}O_{40}]\cdot 13H_2O$

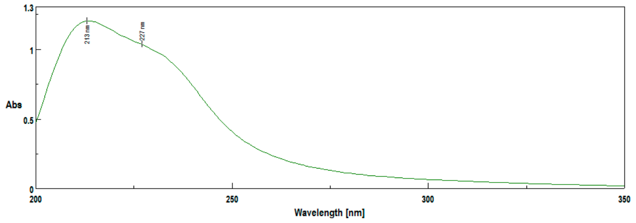

Figure S36. UV electronic spectrum of POM 6, $Na_6[PMo_9^{VI}V_3^{V}O_{40}]\cdot 16H_2O$, Keggin with mixed addenda atoms

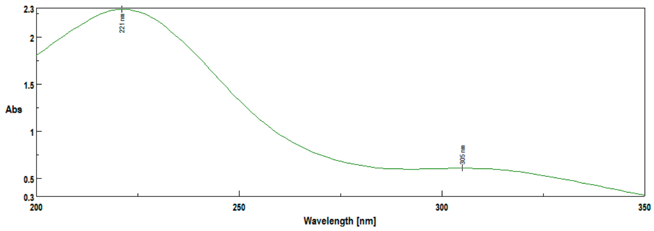

UV electronic spectra of nanoPOMs with mono-lacunary Keggin structures

Figure S37. UV electronic spectrum of POM 1, $Na_4[Fe^{III}(H_2O)PMo_{11}O_{39}]\cdot18H_2O$

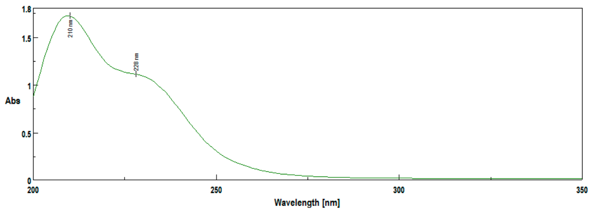

Figure S38. UV electronic spectrum of POM 3, $Na_8[SiW_{11}O_{39}]\cdot12H_2O$

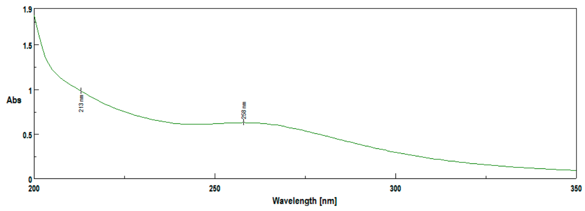

Figure S39. UV electronic spectrum of POM 23a,b, $Na_5[Fe^{III}(H_2O)SiW_{11}O_{39}]\cdot24H_2O$

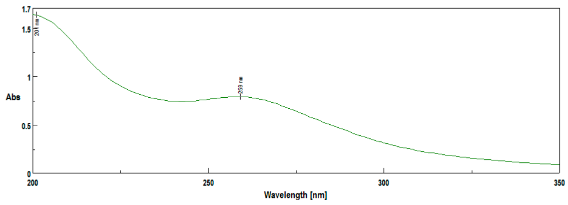

Figure S40. UV electronic spectrum of POM 24a,b, $Na_5[Fe^{III}(H_2O)GeW_{11}O_{39}]\cdot26H_2O$

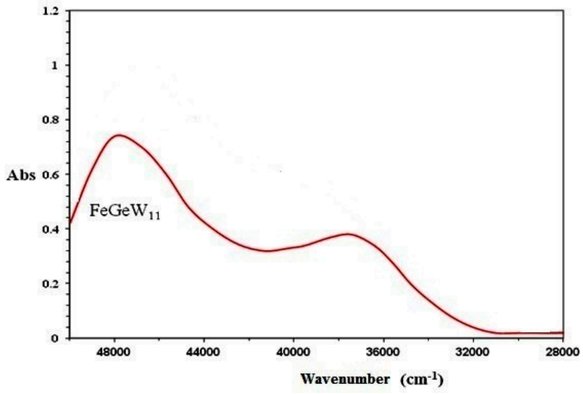

Figure S41. UV electronic spectrum of POM 30, $K_6[SiV^{IV}W_{11}O_{40}]\cdot 12H_2O$

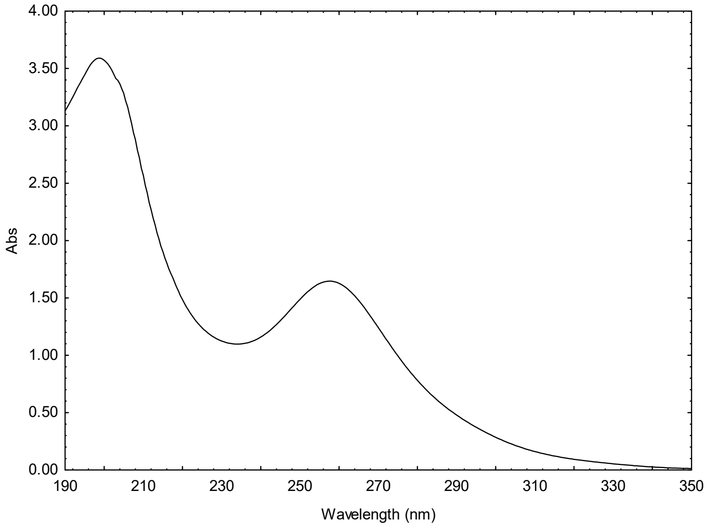

Figure S42. UV electronic spectrum of POM 21, $K_6[Co(H_2O)SiMo_2W_9O_{39}]\cdot 14H_2O$, mono-lacunary Keggin with mixed addenda atoms

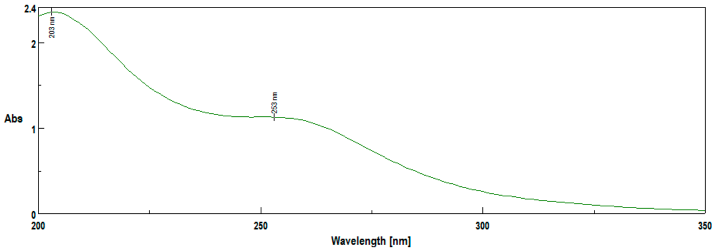

UV electronic spectra of nanoPOMs with tri-lacunary Keggin structures

Figure S43. UV electronic spectrum of POM 5, $K_3[(VO)_3PMo_9O_{34}]\cdot 14H_2O$

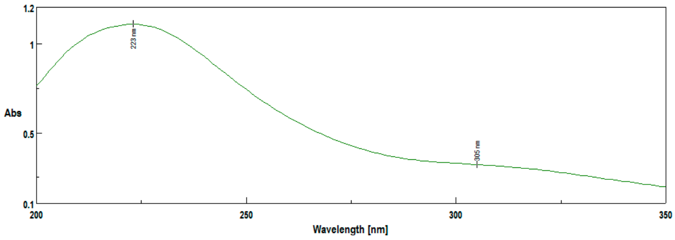

Figure S44. UV electronic spectrum of POM 16a,b, $Na_{10}[SiW_9O_{34}]\cdot 24H_2O$

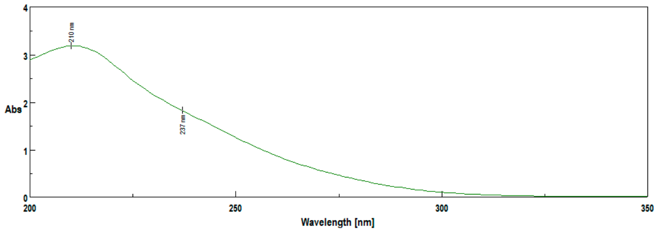

Figure S45. UV electronic spectrum of POM 18, $Na_8H[PW_9O_{34}]\cdot 20H_2O$

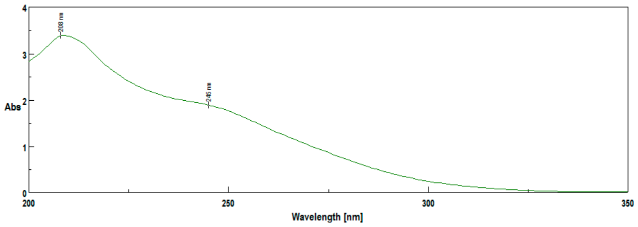

<u>UV electronic spectrum of nanoPOM with tri-lacunary *pseudo*-Keggin structures</u>

Figure S46. UV electronic spectrum of POM 15, $Na_9[SbW_9O_{33}]\cdot 19{,}5H_2O$

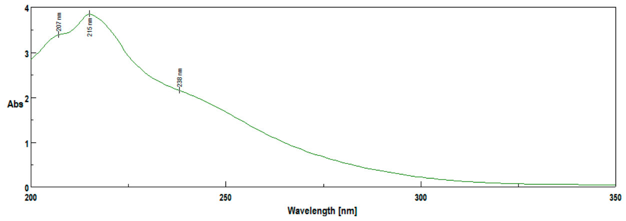

<u>UV electronic spectra of nanoPOMs with tri-lacunary Keggin/sandwich type structures</u>

Figure S47. UV electronic spectrum of POM 2, $Na_9[Fe_3(H_2O)_3(PMo_9O_{34})_2]$

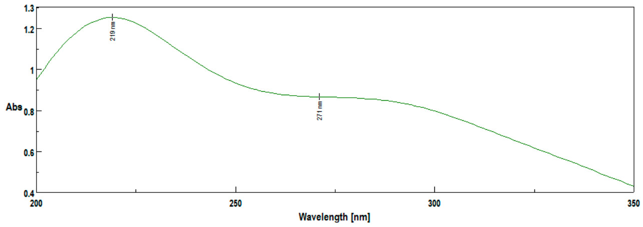

Figure S48. UV electronic spectrum of POM 4, $Na_{11}[Fe_3(H_2O)_3(SiW_9O_{34})_2]\cdot 25H_2O$

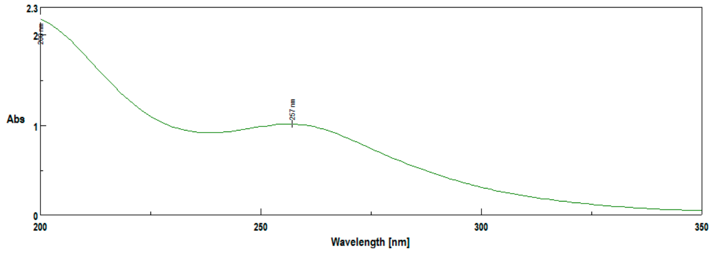

Figure S49. UV electronic spectrum of POM 8, $K_{10}[(VO)_4(PW_9O_{34})_2]\cdot 26H_2O$

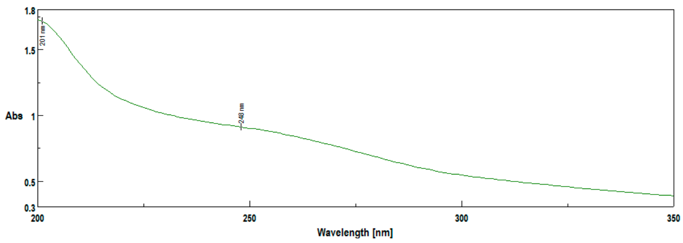

Figure S50. UV electronic spectrum of POM 27, $Na_{14}[Mn_3(H_2O)_3(SiW_9O_{34})_2]\cdot 28H_2O$

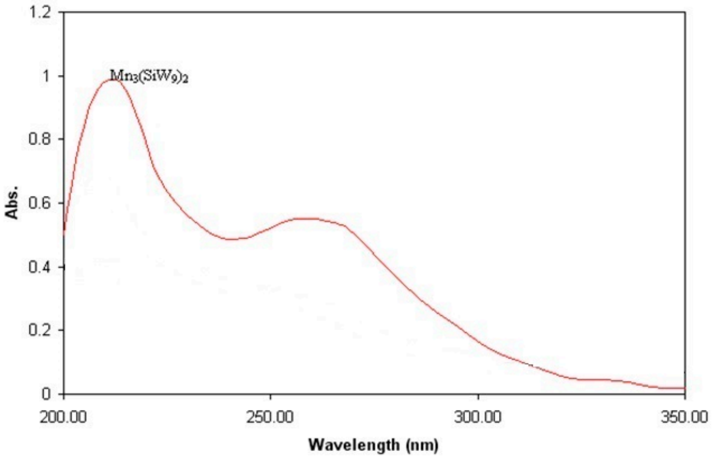

UV electronic spectra of nanoPOMs with tri-lacunary *pseudo*-Keggin/sandwich type structures

UV electronic spectrum of POM 10, $K_{11}H[(VO)_3(Sb^{III}W_9O_{33})_2]\cdot 27H_2O$

Published in our previous paper, cited in this paper as reference 22

Figure S51. UV electronic spectrum of POM 25, $Na_{10}[Mn_4(H_2O)_2(AsW_9O_{34})_2]\cdot 27H_2O$

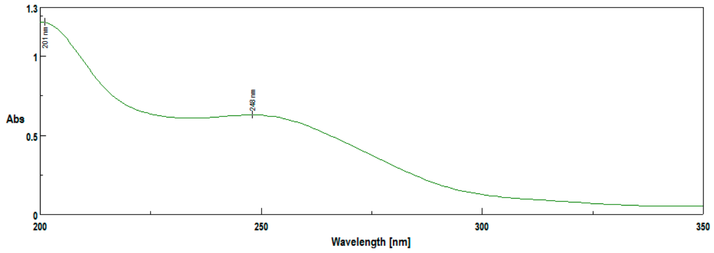

Figure S52. UV electronic spectrum of POM 26, $Na_{12}[Co_3(H_2O)_3(BiW_9O_{33})_2]\cdot 37H_2O$

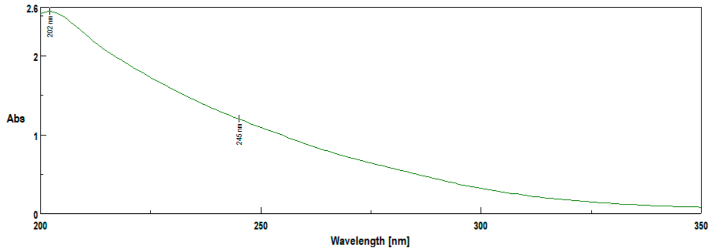

Figure S53. UV electronic spectrum of POM 9, $K_{10}[(VO)_4(AsW_9O_{34})_2]\cdot 21H_2O$

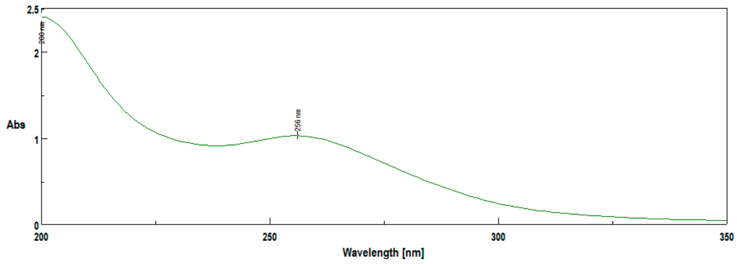

UV electronic spectra of nanoPOMs with cluster structures

Figure S54. UV electronic spectrum of POM 11, $Na_{12}[Sb_2W_{22}O_{74}(OH)_2]\cdot 38H_2O$

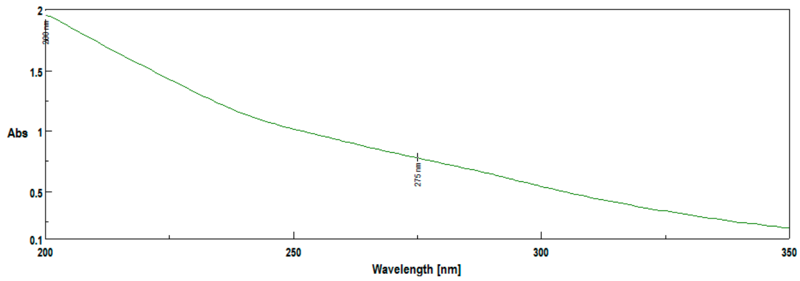

Figure S55. UV electronic spectrum of POM 17, $Na_{27}[NaAs_4W_{40}O_{140}]\cdot 42H_2O$

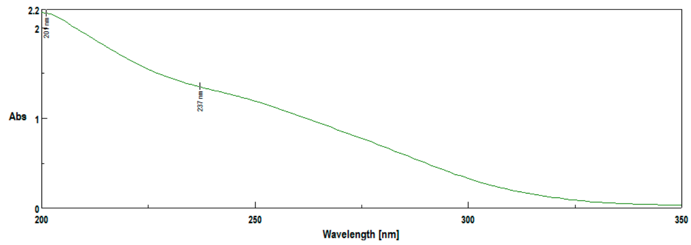

Figure S56. UV electronic spectrum of POM 19, $(NBu_4)_{27}[NaAs_4Mo_{40}O_{140}]\cdot 12H_2O$

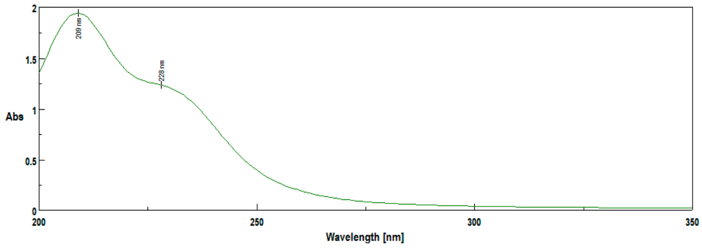

Figure S57. UV electronic spectrum of POM 20, $(Bu_3Sn)_{18}[NaSb_9W_{21}O_{86}]$

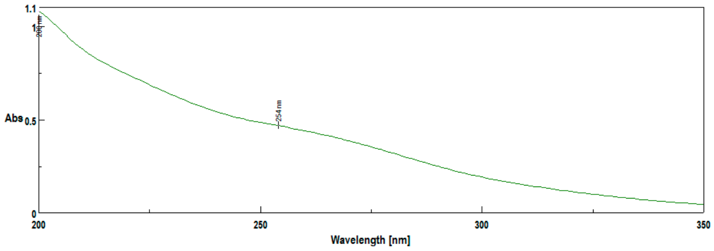

UV electronic spectrum of POM 28, $(NH_4)_4(NBu_4)_5[Na(BuSn)_3Sb_9W_{21}O_{86}]\cdot17H_2O$
Published in our previous paper, cited in this paper as reference 22

Figure S58. UV electronic spectrum of POM 29, $K_{27}[NaAs_4W_{40}O_{140}]\cdot52H_2O$

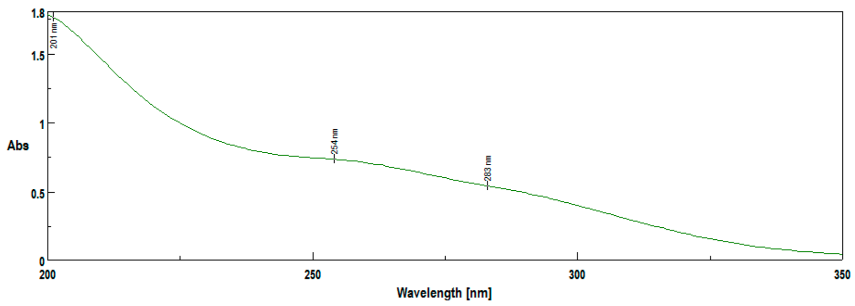

<u>UV electronic spectrum of nanoPOM mono-lacunary Wells-Dawson with mixed addenda atoms</u>

Figure S59. UV electronic spectrum of POM 22, $K_{10}[Co(H_2O)Si_2MoW_{16}O_{61}]\cdot18H_2O$

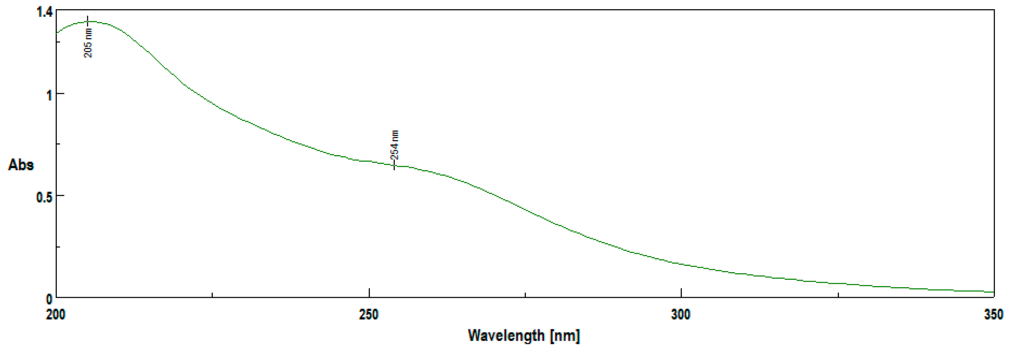

<u>Bacterial inoculi microplates prepared from various reference strains inhibited by the 21 analyzed nanoPOMs</u>

Figure S60. Inhibition aspects for the 21 nanoPOMs analyzed (in microplates of bacterial inoculum prepared from reference strain ATCC 6538P – *Staphylococcus aureus*):
A-C. Active nanoPOMs, D. Control samples.

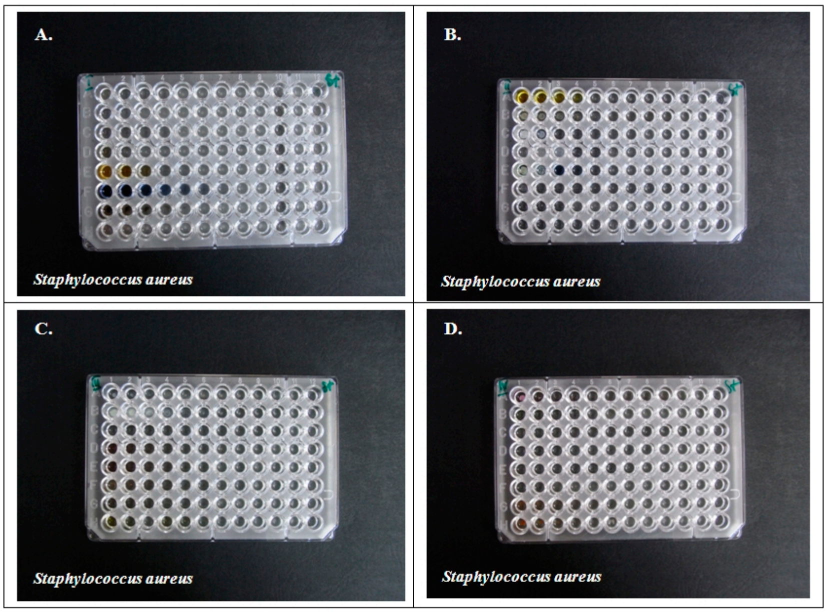

Figure S61. Inhibition aspects for the 21 nanoPOMs analyzed (in microplates of bacterial inoculum prepared from reference strain ATCC 14579 – *Bacillus cereus*):
A-C. Active nanoPOMs, D. Control samples.

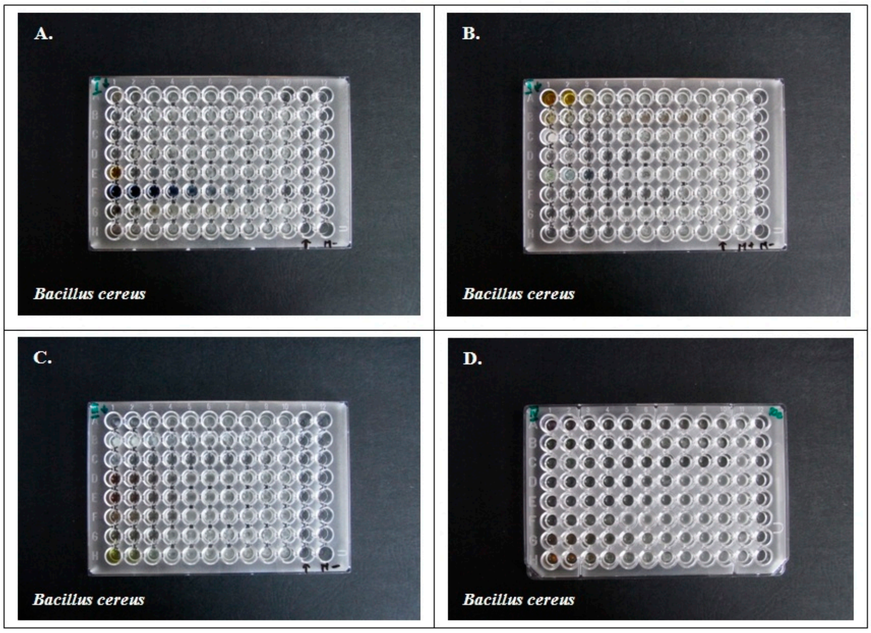

Figure S62. Inhibition aspects for the 21 nanoPOMs analyzed (in microplates of bacterial inoculum prepared from reference strain ATCC 13076 – *Salmonella enteritidis*): A-C. Active nanoPOMs, D. Control samples.

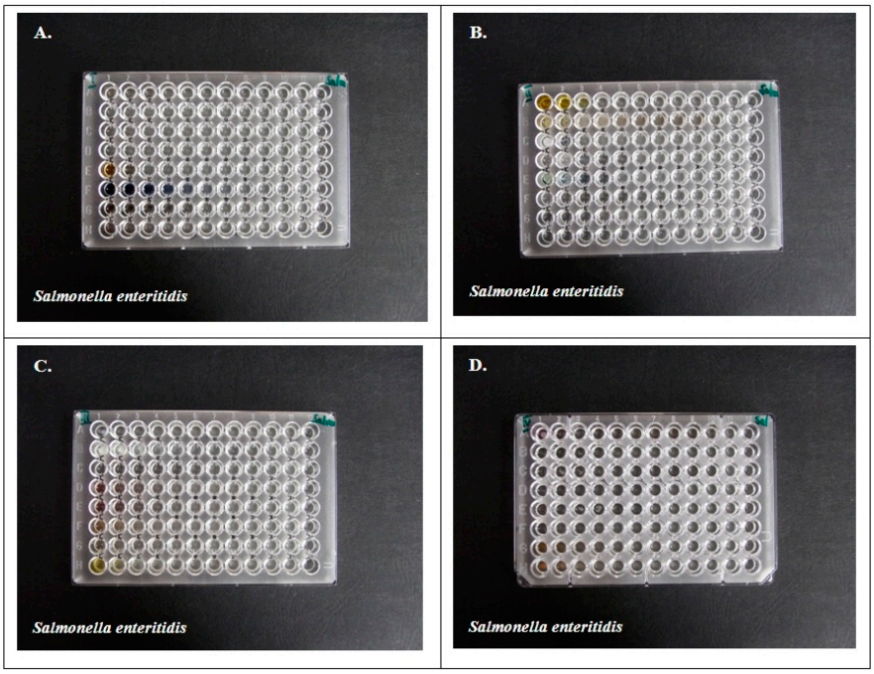

Figure S63. Inhibition aspects for the 21 nanoPOMs analyzed (in microplates of bacterial inoculum prepared from reference strain ATCC 10536 – *Escherichia coli*): A-C. Active nanoPOMs, D. Control samples.

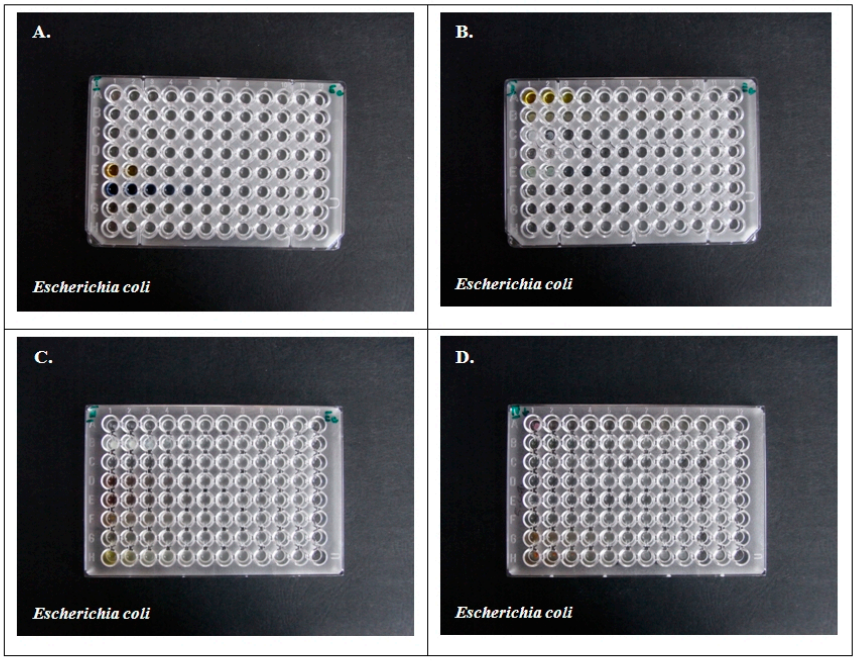

Figure S64. Inhibition aspects for the 21 nanoPOMs analyzed (in microplates of bacterial inoculum prepared from reference strain ATCC 27853 – *Pseudomonas aeruginosa*): A-C. Active nanoPOMs, D. Control samples.

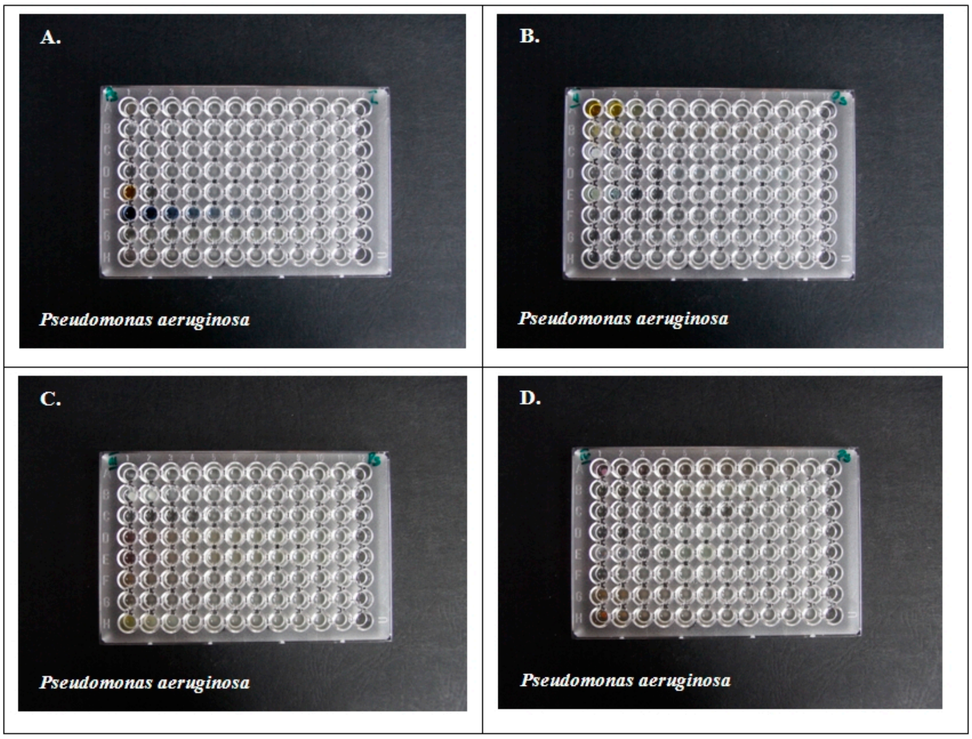

Supplement: Supplementary file 1 [file pharmaceuticals-15-00033-s001.zip › 2021_SUPPLEMENTARY MATERIAL_2.pdf]
