# Supplementary material for: In Vitro Antibacterial Susceptibility of Different Pathogens to Thirty Nano-Polyoxometalates
Source: Pharmaceuticals (Basel). 2021 Dec 27;15(1):33. doi: 10.3390/ph15010033 (PMC8777622; doi:10.3390/ph15010033)
Supplement: Supplementary file 1 [file pharmaceuticals-15-00033-s001.zip › 2021_SUPPLEMENTARY MATERIAL_3.pdf]

<u>SUPPLEMENTARY MATERIAL 3</u>

In vitro Antibacterial Susceptibility of Different Pathogens to Thirty Nano-Polyoxometalates

Ştefana Bâlici[a], Dan Rusu[b], Emőke Páll[c], Miuța Filip[d], Flore Chirilă[e], Gheorghe Zsolt Nicula[a], Mihaela Laura Vică[a,*], Rodica Ungur[f], Horea-Vladi Matei[a], Nicodim Iosif Fiț[e]

[a] "Iuliu Hațieganu" University of Medicine and Pharmacy, Faculty of Medicine, Department of Cell and Molecular Biology, Cluj-Napoca, România
[b] "Iuliu Hațieganu" University of Medicine and Pharmacy, Faculty of Pharmacy, Department of Physical-Chemistry, Cluj-Napoca, România
[c] University of Agricultural Science and Veterinary Medicine, Faculty of Veterinary Medicine, Department of Reproduction, Obstetrics and Veterinary Gynecology, Cluj-Napoca, România
[d] "Babeș-Bolyai" University, "Raluca Ripan" Institute for Research in Chemistry, Analytical and Environmental Chemistry Laboratory, Cluj-Napoca, România
[e] University of Agricultural Science and Veterinary Medicine, Faculty of Veterinary Medicine, Department of Microbiology and Immunology, Cluj-Napoca, România
[f] "Iuliu Hațieganu" University of Medicine and Pharmacy, Faculty of Medicine, Department of Medical Rehabilitation, Cluj-Napoca, România
* Correspondence: mvica@umfcluj.ro

<u>Supplemental Table of Contents</u>

Syntheses of 27 nanoPOMs 2
Syntheses of nanoPOMs with saturated Keggin structures 2
Syntheses of nanoPOMs with mono-lacunary Keggin structures 2
Syntheses of nanoPOMs with tri-lacunary Keggin structures 4
The synthesis of nanoPOM with tri-lacunary *pseudo*-Keggin structures 6
Syntheses of nanoPOMs with tri-lacunary Keggin/sandwich type structures 6
Syntheses of nanoPOMs with tri-lacunary *pseudo*-Keggin/sandwich type structures 7
Syntheses of nanoPOMs with cluster structures 9
The synthesis of nanoPOM, mono-lacunary Wells-Dawson with mixed addenda atoms 11

Syntheses of 27 nanoPOMs

Syntheses of nanoPOMs with saturated Keggin structures

$H_4[SiW_{12}O_{40}]\cdot 14H_2O$ (POM 12), $H_3[PW_{12}O_{40}]\cdot 12H_2O$ (POM 13), $H_3[PMo_{12}O_{40}]\cdot 13H_2O$ (POM 14) were purchased from Sigma-Aldrich (N.V./S.A., Bornem, Belgium or Co. LLC., St. Louis, MO, USA), see Materials and Methods, in this paper.

The synthesis of POM 6, $Na_6[PMo_9^{VI}V_3^{V}O_{40}]\cdot 16H_2O$, Keggin with mixed addenda atoms

1.51 g (1 mmol) of the trilacunary ligand were soluted and 0.55 g (3 mmol) of Na_3VO_4 ortho-vanadate were added, the solution being then acidified with 6 M HCl to a pH of 5.5 and gently stirred at 30 °C for one hour. Following vacuum filtration, the orange filtrate was left to rest at room temperature. After 2 days the very small orange crystals of the compound sedimented. We obtained 0.31 g (0.65 mmol) of $Na_6[PMo_9^{VI}V_3^{V}O_{40}]\cdot 16H_2O$ for a yield of 65%.

$3Na_3VO_4+Na_3[H_6PMo_9O_{34}]+6HCl \rightarrow Na_6[PMo_9V_3O_{40}]+6NaCl+6H_2O$

Syntheses of nanoPOMs with mono-lacunary Keggin structures

The synthesis of POM 1, $Na_4[Fe^{III}(H_2O)PMo_{11}O_{39}]\cdot 18H_2O$

4.12 g (2 mmol) of $H_3[PMo_{12}O_{40}]\cdot 13H_2O$ were dissolved in 25 mL of distilled water, the addition of 1.51 g (18 mmol) of anhydrous $NaHCO_3$ for a pH adjustment to 4.3 of the highly acid solution (initial pH of 1) leading to the quick formation of the monolacunary species $[PMo_{11}O_{39}]^{7-}$. 0.54 g (2 mmol) of $FeCl_3\cdot 6H_2O$ were then added under continuous stirring and gentle heating, the resulting yellow solution being filtered and left to rest at room temperature for three days, until the compound crystals sedimented. Following recrystallization from a minimum amount of hot distilled water 2.82 g (1.28 mmol) of $Na_4[Fe(H_2O)PMo_{11}O_{39}]\cdot 18H_2O$ were obtained for a yield of 64%.

$H_3[PMo_{12}O_{40}]+9NaHCO_3+FeCl_3 \rightarrow Na_4[Fe^{III}(H_2O)PMo_{11}O_{39}]+Na_2MoO_4+9CO_2+3NaCl+5H_2O$

The synthesis of POM 3, $Na_8[SiW_{11}O_{39}]\cdot 12H_2O$

6.26 g (2 mmol) of $H_4[SiW_{12}O_{40}]\cdot 14H_2O$ were dissolved in 50 mL distilled water at room temperature. 1.68 g (20 mmoli) $NaHCO_3$ dissolved in 6 mL of distilled water were dropwise added to the initial solution, which subsequently was stabilized at a pH of 6-7 and left several days at room temperature for the small white crystals to precipitate. Following vacuum filtration the compound was washed in 10 mL 1:1 (v/v) ethanol/methanol, then dried for two hours under vacuum and 24 hours in a desiccator. The compound was purified by recrystallization from hot distilled water, 4.30 g (1.40 mmol) of α-$Na_8[SiW_{11}O_{39}]\cdot 12H_2O$ being obtained at a yield of 70%.

$H_4[SiW_{12}O_{40}]+10NaHCO_3 \rightarrow Na_8[SiW_{11}O_{39}]+Na_2WO_4+10CO_2+7H_2O$

The synthesis of POM 23a,b, $Na_5[Fe^{III}(H_2O)SiW_{11}O_{39}]\cdot 24H_2O$

0.307 g (1.134 mmol) $FeCl_3$ were dissolved under stirring in 11 mL 2M NaCl warm solution. 3.50 g (1,137 mmol) $Na_8[SiW_{11}O_{39}]\cdot 12H_2O$ were then added to this solution and the mixture was stirred at 80 °C for 5 minutes before adjusting the pH to 1.5-2 with 6 M HCl. The clear yellow solution was left to rest at room temperature. After 3 days, the yellow cubic crystals deposited on the bottom of the beaker were filtered through a Büchner funnel and washed with 5 mL methanol, then dried for two hours under vacuum and then kept in the desiccator for 24 hours. Following recrystallization from a minimum amount of hot distilled water 1.79 g of compound were obtained at a yield of 48%.

$\alpha\text{-}Na_8[SiW_{11}O_{39}]+FeCl_3+H_2O\rightarrow \alpha\text{-}Na_5[Fe^{III}(H_2O)SiW_{11}O_{39}]+3NaCl$

The synthesis of POM 24a,b, $Na_5[Fe^{III}(H_2O)GeW_{11}O_{39}]\cdot 26H_2O$

0.33 g (2 mmol) of Na_2GeO_3 and 7.26 g (22 mmol) $Na_2WO_4\cdot 2H_2O$ were dissolved in 40 mL of distilled water in a round-bottomed flask fitted with an ascending refrigerant and a continuous stirring system. The solution was brought to a pH of 6-6.5 by adding 12 mL of 6 M HCl, then kept under stirring and reflux for one hour, then filtered and left to rest for three days at room temperature. The resulting white crystals were filtered on a Büchner funnel, washed with 10 mL of methanol and dried for 2 hours under vacuum and 24 hours in a desiccator.

Following recrystallization from a minimum amount of hot distilled water 0.27 g (0.84 mmoli) of the $\alpha\text{-}Na_8[GeW_{11}O_{39}]\cdot 12H_2O$ ligand were obtained at a yield of 42%.

$11WO_4^{2-}+GeO_3^{2-}+16H^+\rightarrow[\alpha\text{-}GeW_{11}O_{39}]^{8-}+8H_2O$

0.301 g (1,112 mmol) of $FeCl_3$ were dissolved under stirring in 11 mL 2 M NaCl warm solution. 3.5 g (1,115 mmol) $Na_8[GeW_{11}O_{39}]\cdot 12H_2O$ were added to this solution and the mixture was stirred at 80 °C for 5 minutes before adjusting the pH to 2-2.5 with 6 M HCl.

The clear yellowish solution was left to rest at room temperature. After 4 days, the yellow crystals deposited on the bottom of the beaker were filtered through a Büchner funnel and washed with 5 mL methanol, then dried for two hours under vacuum and kept in the desiccator for 24 hours. Following recrystallization from a minimum amount of hot distilled water 1.83 g of compound were obtained at a yield of 49%.

$\alpha\text{-}Na_8[GeW_{11}O_{39}]+FeCl_3+H_2O\rightarrow\alpha\text{-}Na_5[Fe^{III}(H_2O)GeW_{11}O_{39}]+3NaCl$

The synthesis of POM 30, $K_6[SiV^{IV}W_{11}O_{40}]\cdot 12H_2O$

$K_6[SiV^{IV}W_{11}O_{40}]\cdot 12H_2O$ was synthesized by adding vanadyl sulfate to a sodium salt solution of the trilacunary α-Keggin anion.

5.5 g (2,070 mmol) of $Na_{10}[SiW_9O_{34}]\cdot 12H_2O$ (was obtained after Finke method [1]) were

dissolved in 20 mL of distilled water. 0.25 g (1 mmol) of $VOSO_4 \cdot 5H_2O$ dissolved in 10 mL of distilled water were then slowly added under stirring, while the pH was adjusted to 5 with a 0.5 M H_2SO_4 solution. The mixture was heated at 50 °C for 20 minutes, then cooled to room temperature and 5.96 g (80 mmol) of finely ground KCl were added under stirring to precipitate the compound. The solution was then left at rest for 2 hours. The brown powder obtained following vacuum filtration was washed with 2 M KCl. After five days at room temperature, the brown parallelepiped crystals of $K_6[SiV^{IV}W_{11}O_{40}] \cdot 12H_2O$ sedimented at the bottom of the flask and were collected.

The synthesis of the $K_6[SiV^{IV}W_{11}O_{40}] \cdot 12H_2O$ complex started from the trilacunary ligand B-α-$[SiW_9O_{34}]^{9-}$ and the $(VO)^{2+}$ ion combined in a 2:1 molar ratio, according to the following reactions:

$VOSO_4+2Na_{10}[SiW_9O_{34}]+6H_2SO_4 \rightarrow Na_6[SiV^{IV}W_{11}O_{40}]+SiO_2+7WO_3+6H_2O+7Na_2SO_4$

$Na_6[SiV^{IV}W_{11}O_{40}]+6KCl \rightarrow K_6[SiV^{IV}W_{11}O_{40}]+6NaCl$

The mechanism of the complex formation involves the $V(^{IV})$ cation inserted while transforming the trilacunary Keggin fragment $[\alpha\text{-}SiW_9O_{34}]^{10-}$ into the monolacunary Keggin fragment $[\alpha\text{-}SiW_{11}O_{39}]^{8-}$. Physico-chemical investigations confirmed the substitution of $V(^{IV})$ in the α-Keggin structure and the obtaining of POM 30.

The synthesis of POM 21, $K_6[Co(H_2O)SiMo_2W_9O_{39}] \cdot 14H_2O$, mono-lacunary Keggin with mixed addenda atoms

15.32 g (5 mmol) of $K_8[SiMo_2W_9O_{39}] \cdot 14H_2O$ were dissolved under stirring in a minimum amount of distilled water heated at 70 ºC and the solution was brought to a pH of 4 with 2 M glacial acetic acid. 1.25 g (5 mmol) of $Co(CH_3COO)_2 \cdot 4H_2O$ were dissolved under stirring in 10 mL of distilled water and added drop by drop, turning to red the previous solution. The mixture was cooled to room temperature, filtered under vacuum, then cooled at 2 °C and left to rest. The red crystals of $K_6[Co(H_2O)SiMo_2W_9O_{39}] \cdot 14H_2O$ were separated after 5 days. 10.26 g (3.35 mmol) of the compound were obtained with a 67% yield.

$K_8[SiMo_2W_9O_{39}]+Co(CH_3COO)_2 \rightarrow K_6[Co(H_2O)SiMo_2W_9O_{39}]+2CH_3COOK$

Syntheses of nanoPOMs with tri-lacunary Keggin structures

The synthesis of POM 5, $K_3[(VO)_3PMo_9O_{34}] \cdot 14H_2O$

The synthesis of the $Na_3[H_6PMo_9O_{34}] \cdot 13H_2O$ (ligand) was conducted according to the recipe given by Massart and coworkers [2]. 8.95 g (25 mmol) of $Na_2HPO_4 \cdot 12H_2O$ were dissolved in 35.5 mL of 70% $HClO_4$ (ρ = 1.67 g/cm^3) and 10 mL of distilled water, then cooled to -10 °C and kept for one hour under gentle stirring. 54.44 g (225 mmol) of $Na_2MoO_4 \cdot 2H_2O$ were dissolved in 100 mL of distilled water and added dropwise to the initial solution, then left overnight at 0 °C. Next day the microcrystalline precipitate of $Na_3[H_6PMo_9O_{34}] \cdot 13H_2O$ was filtered, washed with

distilled water and dried. 28.40 g (16.25 mmol) of the compound were formed with a yield of 65%.

$Na_2HPO_4 + 17HClO_4 + 9Na_2MoO_4 \rightarrow Na_3H_6[PMo_9O_{34}] + 17NaClO_4 + 6H_2O$

Synthesis of the $K_3[(VO)_3PMo_9O_{34}]\cdot 14H_2O$ complex: 7.50 g (4 mmol) $Na_9[PMo_9O_{34}]\cdot 13H_2O$ were dissolved in 30 mL of distilled water and added to a previously prepared solution of 3.04 g (12 mmol) of $VOSO_4\cdot 5H_2O$ dissolved at room temperature in 70 mL acetic buffer (CH_3COONa/CH_3COOH) to a pH value of 4.8. The resulting solution was heated at 60 °C for one hour and then filtered. The filtrate was cooled to 50 °C and 2.68 g (36 mmol) of finely ground KCl were added to form a brown precipitate, which was recrystallized from hot distilled water after filtration. 5.79 g (2.88 mmol) of flat brown crystals were formed after 3 days with a synthesis yield of 72%, in regard to the Mo content.

$Na_9[PMo_9O_{34}] + 3VOSO_4 + 9KCl \rightarrow K_3[(VO)_3PMo_9O_{34}] + 9NaCl + 3K_2SO_4$

The synthesis of POM 16a,b, $Na_{10}[SiW_9O_{34}]\cdot 24H_2O$

The 9-silicontungstate (IV) was obtained from components by modifying the recipe presented by Hervé [3]. 18.2 g (55.15 mmol) $Na_2WO_4\cdot 2H_2O$ and 1.1g (9 mmol) Na_2SiO_3 were dissolved under stirring in 20 mL of hot distilled water at 80-100 °C. The resulting solution was acidified to a pH of 9.4 with 13 mL of 6 M HCl. The mixture was boiled for one hour under continuous stirring until one-third of the volume was evaporated, then filtered to remove unreacted reagent traces.

5 g of sodium carbonate dissolved in 15 mL of distilled water were then added under gentle stirring, the resulting precipitate being filtered after one hour, resuspended in 100 mL of 4 M NaCl and refiltered. The compound was washed with 100 mL ethanol and then with 100 mL diethyl ether. 11 g of compound were obtained after drying with a yield of 85%.

$9WO_4^{2-} + SiO_3^{2-} + 10H^+ \rightarrow [\alpha\text{-}SiW_9O_{34}]^{10-} + 5H_2O$.

The synthesis of POM 18, $Na_8H[PW_9O_{34}]\cdot 20H_2O$

The synthesis was conducted according to a recipe given by Massart in 1977 [2].

2.97 g (9 mmol) of $Na_2WO_4\cdot 2H_2O$ were dissolved in 6.25 mL of distilled water, then 0.139 mL (2 mmol) of 85% H_3PO_4 and 0.57 mL (9 mmol) of glacial acetic acid were added dropwise. The resulting white precipitate containing fine crystals was filtered under vacuum and 2.00 g (0.72 mmol) of compound were obtained for a yield of 72%.

$9Na_2WO_4 + 2H_3PO_4 + 9CH_3COOH \rightarrow Na_8H[PW_9O_{34}] + 9CH_3COONa + NaH_2PO_4 + 6H_2O$

The synthesis of nanoPOM with tri-lacunary *pseudo*-Keggin structures

The synthesis of POM 15, $Na_9[SbW_9O_{33}]\cdot 19{,}5H_2O$

The trilacunary ligand $Na_9[SbW_9O_{33}]\cdot 19.5H_2O$ was prepared according to the procedure previously described by Bossing *et al*. [4].
6.60 g (20 mmol) of $Na_2WO_4\cdot 2H_2O$ were dissolved in 20 mL of warm distilled water, then 0.32 g (1.11 mmol) of Sb_2O_3 dissolved in 3.30 mL 6 M HCl were added under continuous stirring at 50 °C until the resulting solution reached a final pH of 7.5. The reaction mixture was refluxed for one hour, after which the resulting colorless solution was cooled to room temperature. The microcrystals formed after the evaporation of one third of the solution volume were filtered on a medium porosity glass frit and dried.
4.58 g (1.60 mmol) of compound were formed at a yield of 72%.
$18Na_2WO_4+Sb_2O_3+18HCl \rightarrow 2Na_9[SbW_9O_{33}]+18NaCl+9H_2O$

Syntheses of nanoPOMs with tri-lacunary Keggin/sandwich type structures

The synthesis of POM 2, $Na_9[Fe_3(H_2O)_3(PMo_9O_{34})_2]$

0.81 g (3 mmol) of $FeCl_3\cdot 6H_2O$ were dissolved in 30 mL of hot 2 M NaCl under stirring, then 3.75 g (2 mmol) of $Na_9[PMo_9O_{34}]\cdot 13H_2O$ were added to the solution and stirred for 5 minutes at 60 ºC and the solution pH was adjusted to 3.5 with 6 M HCl. The precipitate traces were filtered off and the filtrate was left at room temperature in a crystallizer. After 5 days, the yellow sediment was filtered and washed with 5 mL of methanol, then dried for two hours under vacuum and 24 hours in the desiccator. The compound was recrystallized from a minimum amount of hot distilled water and 1.79 g (0.48 mmol) were obtained for a yield of 48%.
$2Na_9[PMo_9O_{34}]+3FeCl_3+3H_2O \rightarrow Na_9[Fe_3(H_2O)_3(PMo_9O_{34})_2]+9NaCl$

The synthesis of POM 4, $Na_{11}[Fe_3(H_2O)_3(SiW_9O_{34})_2]\cdot 25H_2O$

0.81 g (3 mmol) of $FeCl_3\cdot 6H_2O$ were dissolved in 10 mL warm distilled water under stirring, then 5.78 g (2 mmol) of the $Na_{10}[SiW_9O_{34}]\cdot 24H_2O$ ligand were added to the solution under gentle stirring at 50 ºC and the solution pH was adjusted to 3.5 with 6 M HCl. The yellow precipitate was filtered on medium pores filter paper. The precipitate was washed with distilled water cooled to 5 °C, then recrystallized from 15 mL of hot water at 40-50 °C. The filtrate was left to cool at room temperature and yellow-orange crystals sedimented after a week. The precipitate was filtered under vacuum and washed with cold distilled water, then dryed.
2.80 g (0.52 mmol) of α-$Na_{11}[Fe_3(H_2O)_3(SiW_9O_{34})_2]\cdot 25H_2O$ were obtained with a 52% yield.
$2\alpha\text{-}Na_{10}[SiW_9O_{34}]+3FeCl_3+3H_2O \rightarrow \alpha\text{-}Na_{11}[Fe_3(H_2O)_3(SiW_9O_{34})_2]+9NaCl$

The synthesis of POM 8, $K_{10}[(VO)_4(PW_9O_{34})_2]\cdot 26H_2O$

2.02 g (8 mmol) of $VOSO_4\cdot 5H_2O$ were dissolved in 10 mL of water at 50 °C, then dropwise added under stirring to a warm solution of 11.19 g (4 mmol) of $Na_9[PW_9O_{34}]\cdot 20\ H_2O$ disolved in 30 mL of distilled water. The resulting solution was stirred at 50 °C for one hour and its pH was corrected to 4.5 with 1 M H_2SO_4, then filtered under vacuum without cooling to remove unreacted reagent traces before 1.49 g (20 mmol) of finely ground KCl were added to the filtrate and left at room temperature. After three days, the very small light brown crystals that sedimented were recrystallized from a minimum amount of hot distilled water.

6.98 g (1.25 mmol) of $K_{10}[(VO)_4(PW_9O_{34})_2]\cdot 26H_2O$ were obtained with a yield of 62.50%.

$2Na_9[PW_9O_{34}]+4VOSO_4+10KCl\rightarrow K_{10}[(VO)_4(PW_9O_{34})_2]+4Na_2SO_4+10NaCl$

The synthesis of POM 27, $Na_{14}[Mn_3(H_2O)_3(SiW_9O_{34})_2]\cdot 28H_2O$

5.78 g (2 mmol) of the $Na_{10}[SiW_9O_{34}]\cdot 24H_2O$ ligand [3] were slowly added under stirring to a solution obtained by dissolving 0.59 g (3 mmol) of $MnCl_2\cdot 4H_2O$ in 10 mL of distilled water, brought to 50 °C and a pH of 4.5. The mixture was maintained 15 minutes under gentle stirring at 50 °C and the resulting orange-yellow precipitate was filtered under vacuum on medium pore filter paper, washed with distilled water cooled to 5 °C, and was recrystallized in 15 mL of warm water at a temperature of 40-50 °C. The solution was left to rest at room temperature and yellow-orange crystals deposited after a week. The recrystalized compound was filtered under vacuum, washed with a little cold distilled water, dried at room temperature and weighed to give 3.19 g (0.58 mmol) of $Na_{14}[Mn_3(H_2O)_3(SiW_9O_{34})_2]\cdot 28H_2O$ at a yield of 58%, relative to the manganese content.

$2\alpha\text{-}Na_{10}[SiW_9O_{34}]+3MnCl_2+3H_2O\rightarrow \alpha\text{-}Na_{14}[Mn_3(H_2O)_3(SiW_9O_{34})_2]+6NaCl$

<u>Syntheses of nanoPOMs with tri-lacunary *pseudo*-Keggin/sandwich type structures</u>

The synthesis of POM 10, $K_{11}H[(VO)_3(Sb^{III}W_9O_{33})_2]\cdot 27H_2O$

$K_{11}H[(VO)_3(SbW_9O_{33})_2]\cdot 27H_2O$, the potassium salt of the tris-(vanadyl)-18-tungsten-2-stibiatum anion (III), also known as PM-1002 (POM 1 diabetes in our previous paper [22]), was synthesized according to the method of Yamase published in 2001 [5].

11.45 g (4 mmol) of the $Na_9[Sb^{III}W_9O_{33}]\cdot 19.5H_2O$ ligand (obtained according to literature data) were added to a pre-prepared solution of 1.52 g (6 mmol) $VOSO_4\cdot 5H_2O$ in 35 mL acetic buffer (CH_3COONa/CH_3COOH), brought up to a pH value of 4.8. The resulting dark brown solution was heated at 60-70 °C for one hour and then filtered. The filtrate was cooled to room temperature, then 1.64 g (22 mmol) of finely ground KCl was added to form a brown precipitate,

which was then recrystallized from hot distilled water.
The flat brown crystals of 7.42 g (1.30 mmol) were formed after 3 days with a synthesis yield of 65% relative to the W content, lower than the yield obtained by Yamase.

$2Na_9[SbW_9O_{33}]+3VOSO_4+CH_3COOH+11KCl \rightarrow K_{11}H[(VO)_3(SbW_9O_{33})_2]+11NaCl+3Na_2SO_4+CH_3COONa$

The synthesis of POM 25, $Na_{10}[Mn_4(H_2O)_2(AsW_9O_{34})_2]\cdot 27H_2O$

After complete dissolution at 70 °C of 2.68 g (1 mmol) $Na_9[AsW_9O_{34}]\cdot 11\ H_2O$ in 7.5 mL of distilled water, a pre-prepared solution of 0.49 g (2 mmol) of $Mn(CH_3COO)_2\cdot 4H_2O$ in 5 mL distilled water was gradually added under stirring. The mixture was kept at 70 °C another 15 minutes, the resulting yellow-orange solution (pH 6.8) being then filtered through a medium porosity glass fryer and cooled to room temperature. After nine days, the sedimented yellow-orange microcrystals were filtered and washed with a solution of 2 M NaCl, ethanol and ether. 2.04 g (0.37 mmol) of the $Na_{10}[Mn_4(H_2O)_2(AsW_9O_{34})_2]\cdot 27H_2O$ nanocompound were obtained with a yield of 74% relative to the manganese content. The compound was recrystallized from a minimum amount of hot distilled water.

$2Na_9[AsW_9O_{34}]+ 4(CH_3COO)_2Mn+2H_2O \rightarrow Na_{10}[Mn_4 (H_2O)_2(AsW_9O_{34})_2]+8CH_3COONa$

The synthesis of POM 26, $Na_{12}[Co_3(H_2O)_3(BiW_9O_{33})_2]\cdot 37H_2O$

The synthesis was performed using the "all in one pot" method, by modifying the methods mentioned above in the literature [4, 6].
0.97 g (2 mmol) of $Bi(NO_3)_3\cdot 5H_2O$ dissolved in 1.5 mL 6 M HCl were gradually added, in small portions, to a hot solution (brought to 80 ºC) of $Na_2WO_4\cdot 2H_2O$ (5.94 g, 18 mmol, dissolved in 48 mL bidistilled water), at a pH of 7.50-8.00, under vigorous stirring. After one hour the mixture was cooled to 50 °C and the $CoCl_2\cdot 6H_2O$ salt solution (3 mmol in 40 mL of bidistilled water) was added gradually to finally reach a molar ratio of Bi:Co = 2:3. The resulting solution was further stirred for 15 minutes at 50 °C, then cooled to room temperature and filtered under vacuum using a medium porosity fryer. After a few days at room temperature, the deposited nanocompound precipitate was filtered and washed with a 2 M NaCl solution, then with ethanol and ether. For “all in one pot” POM a synthesis yield of ~ 70% relative to the transition metal content was reached.

$18WO_4^{2-}+2Bi^{3+}+12H^+ +3MT^{n+} \rightarrow [(MT)_3^{n+}(H_2O)_a(BiW_9O_{33})_2]^{(18-3n)-} +3H_2O$, n=3, a=3.

The synthesis of POM 9, $K_{10}[(VO)_4(AsW_9O_{34})_2]\cdot 21H_2O$

The synthesis of B-α-$Na_9[AsW_9O_{34}]\cdot 11H_2O$ was conducted using a modified recipe of that given by Finke [1]. 32.98 g (100 mmoli) $Na_2WO_4\cdot 2H_2O$ were dissolved in 40 mL of distilled water, then 3.67 g (11 mmol) of $Na_3AsO_4\cdot 7H_2O$ were added under continuous stirring, followed by the addition of 14.00 mL of glacial acetic acid. The reaction mixture (pH of 7.5) was refluxed for two hours, the resulting colorless solution being then filtered under vacuum and left to cool at

room temperature. After three days the white precipitate formed was collected on a glass fryer and dried under vacuum.

$9Na_2WO_4+Na_3AsO_4+12CH_3COOH \rightarrow Na_9[AsW_9O_{34}]+12CH_3COONa+6H_2O$

After complete dissolution, at 70 °C, of 5.36 g (2 mmol) $Na_9[AsW_9O_{34}]\cdot 11H_2O$ in 16 mL of distilled water, 1.01 g (4 mmol) of $VOSO_4\cdot 5H_2O$ soluted in 10 mL of distilled water were dropwise added under stirring and the mixture was maintained at 70 °C for another 15 minutes. The resulting dark brown solution with a pH of 6.7 was filtered, then 0.76 g (10 mmol) of finely ground KCl were added until the dark brown solution above the precipitate remained clear. After one week, the brown microcrystals were filtered off and successively washed with a 2 M KCl solution, ethanol and ether.

4.02 g (0.72 mmol) of $K_{10}[(VO)_4(AsW_9O_{34})_2]\cdot 21H_2O$ were obtained with a yield of 72% (relative to the vanadium content). The compound was recrystallized from a minimum amount of hot water (550 mg/5 mL).

$2Na_9[AsW_9O_{34}]+4VOSO_4+10KCl \rightarrow K_{10}[(VO)_4(AsW_9O_{34})_2]+10NaCl+4Na_2SO_4$

Syntheses of nanoPOMs with cluster structures

The synthesis of POM 11, $Na_{12}[Sb_2W_{22}O_{74}(OH)_2]\cdot 38H_2O$

Using the method given by Krebs for the synthesis of the compounds that bear his name, we synthesized this cluster.

10.85 g (3.79 mmol) of $Na_9[SbW_9O_{33}]\cdot 19.5H_2O$ and 2.5 (7.58 mmol) of $Na_2WO_4\cdot 2H_2O$ were dissolved in distilled water (10 mL) under stirring and gentle heating. The pH of the resulting solution was adjusted to 4.8 by adding 4.42 mL of 6 M HNO_3. The solution was boiled until 2/3 of the volume evaporatated. After cooling to room temperature, the solution was filtered under vacuum and left to rest. After two days the microcrystalline precipitate formed was collected on a glass fryer and dried under vacuum. 7.57 g (1.17 mmol) of the compound were obtained with a yield of 62%. It was recrystallized from a minimum amount of hot distilled water.

$2Na_9[SbW_9O_{33}] + 4Na_2WO_4 + 14HNO_3 \rightarrow Na_{12}[Sb_2W_{22}O_{74}(OH)_2] + 6H_2O + 14NaNO_3$

The synthesis of POM 17, $Na_{27}[NaAs_4W_{40}O_{140}]\cdot 42H_2O$

33 g (100 mmol) $Na_2WO_4\cdot 2H_2O$ were dissolved in 100 mL of distilled water, adjusted with glacial acetic acid to a pH of 4.5, and brought to boiling temperature, when a solution obtained by dissolving 1 g (5 mmol) of As_2O_3 in 13 mL of 11 M HNO_3 at 90 °C was dropwise added under continuous stirring. 0.2 g (2.68 mmol) of NaCl were then added and the resulting clear solution was maintained at 90 ° C for one hour, any impurities being filtered while the solution was hot. The filtrate was allowed to crystallize slowly at room temperature. After 4 days, the resulting colorless crystals were filtered and purified by recrystallization from distilled water

brought to a pH of 4.5 with glacial acetic acid. Finally, 20.44 g (1.81 mmol) of the compound were obtained with a yield of 72.40%.
$40Na_2WO_4 + 2As_2O_3 + 52HCl \rightarrow Na_{27}[NaAs_4W_{40}O_{140}] + 52NaCl + 26H_2O$

The synthesis of POM 19, $(NBu_4)_{27}[NaAs_4Mo_{40}O_{140}]\cdot 12H_2O$

1 mol of $Na_2MoO_4\cdot 2H_2O$ (241.95 g) was dissolved in 500 mL of distilled water at 70ºC, then 13 g (0.1 mol) of sodium arsenite $NaAsO_2$ were added dropwise under strong stirring and the solution pH was adjusted to 4 by adding 250 mL of 6 M HCl. The solution was boiled until a final volume of 500 mL was reached, then cooled to 5 ºC to sediment. The dirty white precipitate was filtered, washed with 2 M NaCl solution and left at room temperature. After three days the precipitate color shifted to yellowish-green.
16.68 g (0.0021 mol) of $Na_{27}[NaAs_4Mo_{40}O_{140}]\cdot 46H_2O$ were obtained with a final synthesis yield of 85%.
$40Na_2MoO_4 + 4NaAsO_2 + 56HCl \rightarrow Na_{27}[NaAs_4Mo_{40}O_{140}] + 56NaCl + 28H_2O$
9.23 g (28.62 mmol) of $(NBu_4)Br$ were added to a solution formed by dissolving 8.34g (1.06 mmol) of $Na_{27}[NaAs_4Mo_{40}O_{140}]\cdot 46H_2O$ in hot distilled water. The resulting solution was further boiled at 70 °C for 90 minutes, then cooled to 50 °C, filtered under vacuum and washed with a saturated solution of $(NBu_4)Br$. The filtrate was left to rest at room temperature. After one week, the sedimented microcrystals were filtered, washed with distilled water and dried under vacuum. 6.19 g (0.47 mmol) of the compound were obtained for a yield of 44%.
$Na_{27}[NaAs_4Mo_{40}O_{140}] + 27(NBu_4)Br \rightarrow (NBu_4)_{27}[NaAs_4Mo_{40}O_{140}] + 27NaBr$

The synthesis of POM 20, $(Bu_3Sn)_{18}[NaSb_9W_{21}O_{86}]$

6.94 g (1 mmol) $(NH_4)_{17}Na[NaSb_9W_{21}O_{86}]\cdot 14H_2O$ (ligand) were rapidly added (under continuous stirring at 50-60 °C) to a solution which contains 18 mmol (3.60 mL) of $(C_4H_9)_3SnCl$ ($\rho = 1.2$ g/cm^3) in 50 mL bidistilled and deionized water.
$(NH_4)_{17}Na[NaSb_9W_{21}O_{86}] + 18Bu_3SnCl \rightarrow (Bu_3Sn)_{18}[NaSb_9W_{21}O_{86}] + 17NH_4Cl + NaCl$

The synthesis of POM 28, $(NH_4)_4(NBu_4)_5[Na(BuSn)_3Sb_9W_{21}O_{86}]\cdot 17H_2O$

Published in our previous paper, cited in this paper as reference 22.
The $(NH_4)_{17}Na[NaSb_9W_{21}O_{86}]\cdot 14H_2O$, also known as HPA-23, was prepared according to literature data [7].
9.58 g (42 mmol) of $SbCl_3$ were dissolved in 50 mL of NH_4Cl saturated solution and dropwise added to 120 mL of 0.15 M $Na_2WO_4\cdot 2H_2O$ pre-heated at 80 °C, then concentrated NH_4OH was added until the solution became colorless. The solution was left to rest several days at room temperature, then the microcrystalline precipitate was filtered under vacuum and subsequently

washed with a diluted NH_4Cl solution. The crystals were purified by recrystallization from a minimum amount of hot distilled water. 8.05 g (1.16 mmol) of the compound were obtained for a reaction yield of 58%.

$21Na_2WO_4 + 9SbCl_3 + 13NH_4Cl + 4NH_4OH \rightarrow (NH_4)_{17}Na[NaSb_9W_{21}O_{86}] + 40NaCl + 2H_2O$

6.94 g (1 mmol) of $(NH_4)_{17}Na[NaSb_9W_{21}O_{86}]\cdot 14H_2O$ ligand powder were rapidly added under continuous stirring at 50-60 °C to a solution of 0.66 mL (3.3 mmol) of n-$C_4H_9SnCl_3$ in 50 mL of double-distilled and deionized water. Within seconds, most of the Sb_9W_{21} cryptate polyanion was dissolved and the solution became clear at a final pH of 5.0. Traces of unreacted ligand were filtered off after 10 minutes, then 2.7 mmol (1 g) of $(NBu_4)Br$ were added to the filtrate to produce a white precipitate which was then collected on a fine glass frit, dried under vacuum for 1 h, and subsequently washed with a saturated solution of $(NBu_4)Br$. The precipitate was left to dry under vacuum overnight and 3.60 g were obtained for a yield of 39%. The white powder was washed with ethanol and 2.94 g of high purity compound were obtained with a final yield of 31%.

The $BuSn^{3+}$ ions reacted in aqueous solution with $[NaSb_9W_{21}O_{86}]^{18-}$ ions according to the reaction:

$(NH_4)_{17}Na[NaSb_9W_{21}O_{86}] + 3BuSnCl_3 \rightarrow (NH_4)_9[Na(BuSn)_3Sb_9W_{21}O_{86}] + 8(NH_4)Cl + NaCl$

The final product was isolated as a mixed ammonium and tetrabutyl-ammonium salt with a good yield according to the following equation:

$(NH_4)_9[Na(BuSn)_3Sb_9W_{21}O_{86}] + 5(NBu_4)Br \rightarrow (NH_4)_4(NBu_4)_5[Na(BuSn)_3Sb_9W_{21}O_{86}] + 5(NH_4)Br$

The synthesis of POM 29, $K_{27}[NaAs_4W_{40}O_{140}]\cdot 52H_2O$

3.02 g (40.50 mmol) of finely ground KCl were added to a solution formed by dissolving 16.94 g (1.50 mmol) of $Na_{27}[NaAs_4W_{40}O_{140}]\cdot 42H_2O$ in hot distilled water and the mixture was boil one hour at 70 °C, then filtered under vacuum and washed with a solution of 2 M KCl, the filtrate being left to cool to room temperature. The compound crystals sedimented after 3 days and were recrystallized from a minimum amount of hot distilled water.

9.29 g (0.78 mmol) of the compound were obtained for a yield of 52%.

$Na_{27}[NaAs_4W_{40}O_{140}] + 27KCl \rightarrow K_{27}[NaAs_4W_{40}O_{140}] + 27NaCl$

<u>The synthesis of nanoPOM, mono-lacunary Wells-Dawson with mixed addenda atoms</u>

The synthesis of POM 22, $K_{10}[Co(H_2O)Si_2MoW_{16}O_{61}]\cdot 18H_2O$

1.65 g (5 mmol) $Na_2WO_4\cdot 2H_2O$ were dissolved in hot water and treated with excessive Na_2SiO_3, then brought to a pH of 4 by adding 0.5 M H_2SO_4. The solution was boiled for 3 hours and 5 drops of HNO_3 were added. Finely ground KCl was stoichiometrically added to precipitate the $K_8[Si_2W_{18}O_{62}]$. 1.50 g of this compound were added to a solution formed by dissolving the stoichiometric amount required to obtain 3 mL 1 M $Na_2MoO_4\cdot 2H_2O$ and 6 mL 6 M HCl and the resulting solution was boiled for 3 hours. The addition of finely ground KCl produced a

yellowish $K_8[Si_2MoW_{17}O_{62}]$ precipitate. 1.00 g of $K_8[Si_2MoW_{17}O_{62}]$ was dissolved in 4.80 mL of distilled water and 3 mL of 0.5M $KHCO_3$ were added to a final pH of 6.5. The light blue solution was filtered under vacuum and washed with a cold 1 M KCl solution. The white crystals of the $K_{12}[Si_2MoW_{16}O_{61}]\cdot 19H_2O$ ligand sedimented.

$18WO_4^{2-}+MoO_4^{2-}+2SiO_3^{2-}+36H^+\rightarrow[Si_2MoW_{17}O_{62}]^{8-}+18H_2O$ (at pH=4);

$[Si_2MoW_{17}O_{62}]^{8-}+3HCO_3^- \rightarrow[Si_2MoW_{16}O_{61}]^{12-}+WO_4^{2-}+3H^++3CO_2$ (pH of 6-6.5).

0.80 g (0.16 mmol) of the $K_{12}[Si_2MoW_{16}O_{61}]\cdot 19H_2O$ ligand were dissolved under continuous stirring in a minimum volume of distilled water heated at 60 °C, and 6 M HCl was added to a final pH value of 4.

0.04 g (0.16 mmol) of $Co(CH_3COO)_2\cdot 4H_2O$ dissolved in 6.50 mL distilled water were added dropwise to this solution, maintained at 60 °C for one hour. The resulting dark red solution was cooled to room temperature and filter under vacuum. $K_{10}[Co(H_2O)Si_2MoW_{16}O_{61}]\cdot 18H_2O$ the red crystals sedimented after 2 days and were purified by recrystallization from the minimum amount of acidified distilled water.

0.5 g (0.103 mmol) of purified compound were obtained for a final yield of 64%.

$K_{12}[Si_2MoW_{16}O_{61}]+Co(CH_3COO)_2+2HCl+H_2O\rightarrow K_{10}[Co(H_2O)Si_2MoW_{16}O_{61}]+2CH_3COOH+2KCl$

References

1. Finke, R.G.; Droege, M.W. Trivacant Heteropolytungstate Derivatives. 2. Synthesis, Characterization, And Tungsten-183 NMR of $P_4W_{30}M_4(H_2O)_2O_{112}^{16-}$ (M = Co, Cu, Zn). *Inorg. Chem.*, **1983**, *22*, 1006–1008, https://doi.org/10.1021/ic00148a038.

2. Massart, R.; Contant, R.; Fruchart, J.M.; Ciabrini, J.F.; Fournier, M. Phosphorus-31 NMR Studies on Molybdic and Tungstic Heteropolyanions. Correlation between Structure and Chemical Shift. *Inorg. Chem.,* **1977,** *16,* 2916–2921, https://doi.org/10.1021/ic50177a049.

3. Hervé, G.; Tézé, A. Study of .Alpha.- and .Beta.-Enneatungstosilicates and –Germanates. *Inorg. Chem.*, **1977**, *16*, 2115–2117, https://doi.org/10.1021/ic50174a060.

4. Bösing, M.; Loose, I.; Pohlmann, H.; Krebs, B. New Strategies for the Generation of Large Heteropolymetalate Clusters: The β-B-SbW_9 Fragment as a Multifunctional Unit. *Chem. Eur. J.*, **1997**, *3*, 1232–1237, https://doi.org/10.1002/chem.19970030810.

5. Botar, B.; Yamase, T.; Ishikawa, E. A Highly Nuclear Vanadium-Containing Tungstobismutate: Synthesis and Crystal Structure of $K_{11}H[(BiW_9O_{33})_3Bi_6(OH)_3(H2O)_3V_4O_{10}]$ $\cdot 25H_2O$. *Inorg. Chem. Commun.*, **2000**, *3*, 579–584, https://doi.org/10.1016/S1387-7003(00)00 146-5.

6. Loose, I.; Droste, E.; Bösing, M.; Pohlmann, H.; Dickman, M.H.; Roşu, C.; Pope, M.T.; Krebs, B. Heteropolymetalate Clusters of the Subvalent Main Group Elements Bi^{III} and Sb^{III} II. *Inorg. Chem.*, **1999**, *38*, 2688–2694, https://doi.org/10.1021/ic981313m.

7. Jasmin, C.; Chermann, J. C.; Hervè, G.; Tézè, A.; Souchay, P.; Boy-Loustau, C.; Raybaud, N.; Sinoussi, F.; Raynaud, M. *In Vivo* Inhibition of Murine Leukemia and Sarcoma Viruses by the Heteropolyanion 5-Tungsto-2- Antimonate. *J. Natl. Cancer Inst.* **1974**, *53*, 469–474, https://doi.org/10.1093/jnci/53.2.469.
